# Total Synthesis of Pagoamide A

**DOI:** 10.3390/molecules26144224

**Published:** 2021-07-12

**Authors:** Fusong Wu, Jie Yu, Jiawei Meng, Yian Guo, Tao Ye

**Affiliations:** 1 State Key Laboratory of Chemical Oncogenomics, Peking University Shenzhen Graduate School, Shenzhen 518055, China; wufusong@pku.edu.cn (F.W.); yujie0701@pku.edu.cn (J.Y.); 1997MJW@pku.edu.cn (J.M.); 2 School of Biotechnology and Health Sciences, Wuyi University, Jiangmen 529020, China

**Keywords:** pagoamide A, total synthesis, cyclic depsipeptides

## Abstract

The first total synthesis of the thiazole-containing cyclic depsipeptide pagoamide A, is detailed. The longest linear sequence of the liquid-phase synthesis comprises 9 long linear steps from simple known starting materials, which led to the unambiguous structural confirmation of pagoamide A.

## 1. Introduction

**Figure 1:**
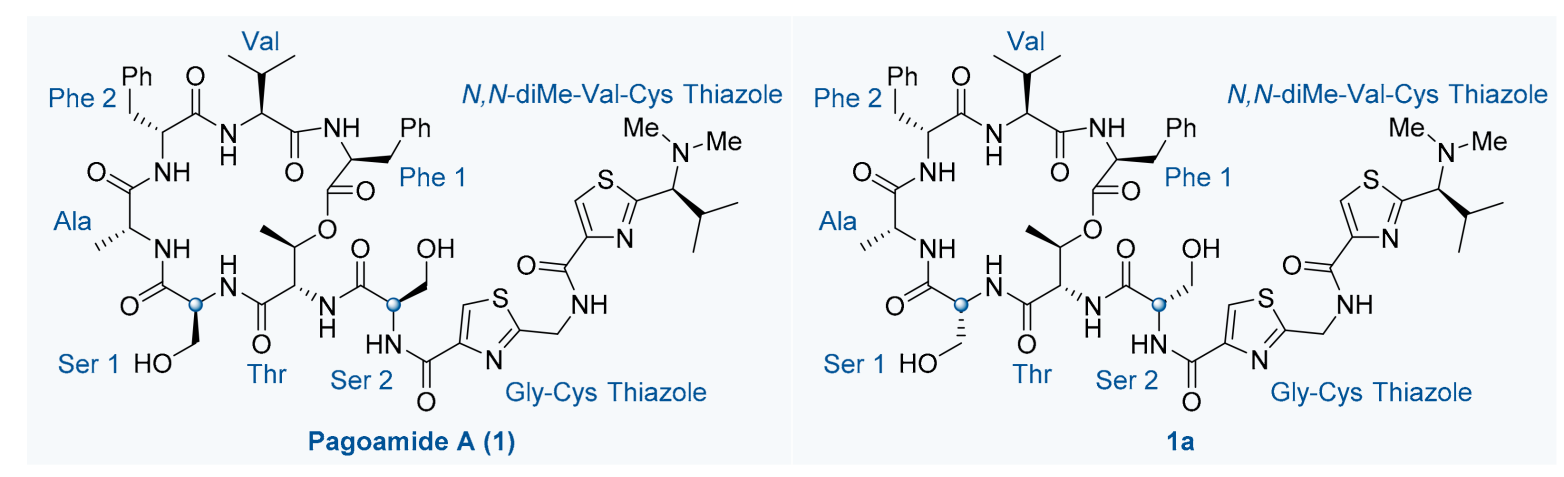
Structure of Pagoamide A (**1**) and its diastereomer (**1a**).

Very recently, Gerwick and co-workers described the structure of pagoamide A [1], a thiazole-containing cyclic depsipeptide isolated from the metabolites of a marine Chlorphyte, *Derbesia* sp., which was collected from the shallow coastal waters near American Samoa and cultured in the laboratory. Preliminary biological tests revealed that pagoamide A had no cytotoxicity against H-460 human lung cancer cells. The isolation of this novel compound was guided by MS/MS-based molecular networking and the structure of pagoamide A (**1**) was elucidated through a combination of detailed 1D and 2D NMR analyses, UV, high-resolution mass spectrometry and advanced Marfey’s method. Noticeably, two phenylalanine residues with opposite stereochemistry were carefully established by chemical methods, whereas the absolute configuration of the serine in the macrocyclic ring and side-chain had only been tentatively assigned by density functional theory (DFT) calculations in combination with ROESY results. As depicted in Figure 1, its unique structure comprises a 19-membered macrolactam and a pendant thiazole-bearing side chain. In total 11 amino acid moieties are incorporated in this depsipeptide with three of them possessing d configurations (d-Val, d-Ala, d-Ser). Motivated by our previous studies of 5-membered heterocycle-containing marine natural products [2,3,4,5,6,7,8,9], we hoped to accomplish the first total synthesis of pagoamide A and in turn, elucidate its relative and absolute stereochemistry. 

**Scheme 1:**
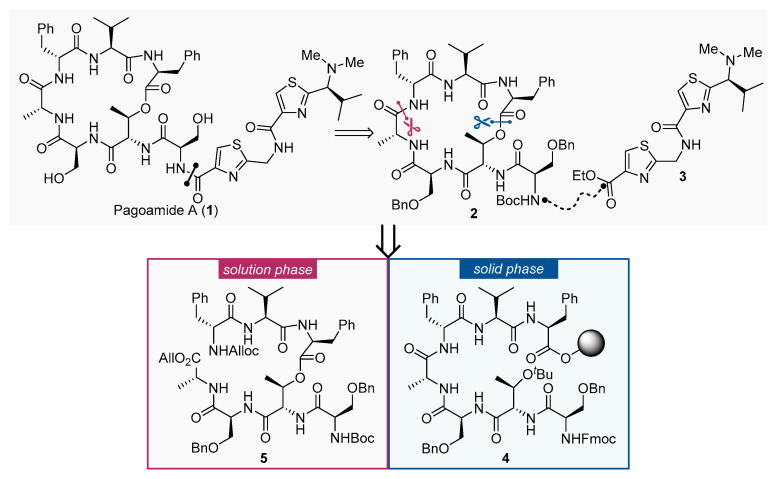
Retrosynthetic analysis of pagoamide A (**1**) and key intermediates **2**–**5**.

As DFT calculations for structural assignments can sometimes be error-prone [10,11,12,13], at the outset of this project, we opted for the synthesis of structure of pagoamide A (**1**) and one of its diastereomers **1a**, where the two serine residues switched positions. The success of this investigation would allow us to further resolve the structure of this natural product. Our retrosynthetic analysis for pagoamide A (**1**) is presented in Scheme 1. The final step involves the connection of both the side chain **3** and the macrocycle **2**. Literature precedents suggested that the ring disconnection of macrocycles, especially macrocyclic peptides, is so strategically important that it can ultimately determine the success of a synthesis [14]. We thus elected to conduct the synthesis of macrocycle **2** via two distinct routes which included macrocyclization performed by lactamization of the amino acid derived from **5** or lactonization of a seco-acid derived from **4**, respectively. We envisioned the precursor **4** would be rapidly assembled through solid phase peptide synthesis (SPPS) and the linear depsipeptide **5** should be available through a more convergent solution-phase synthesis. 

## 2. Results

**Scheme 2:**
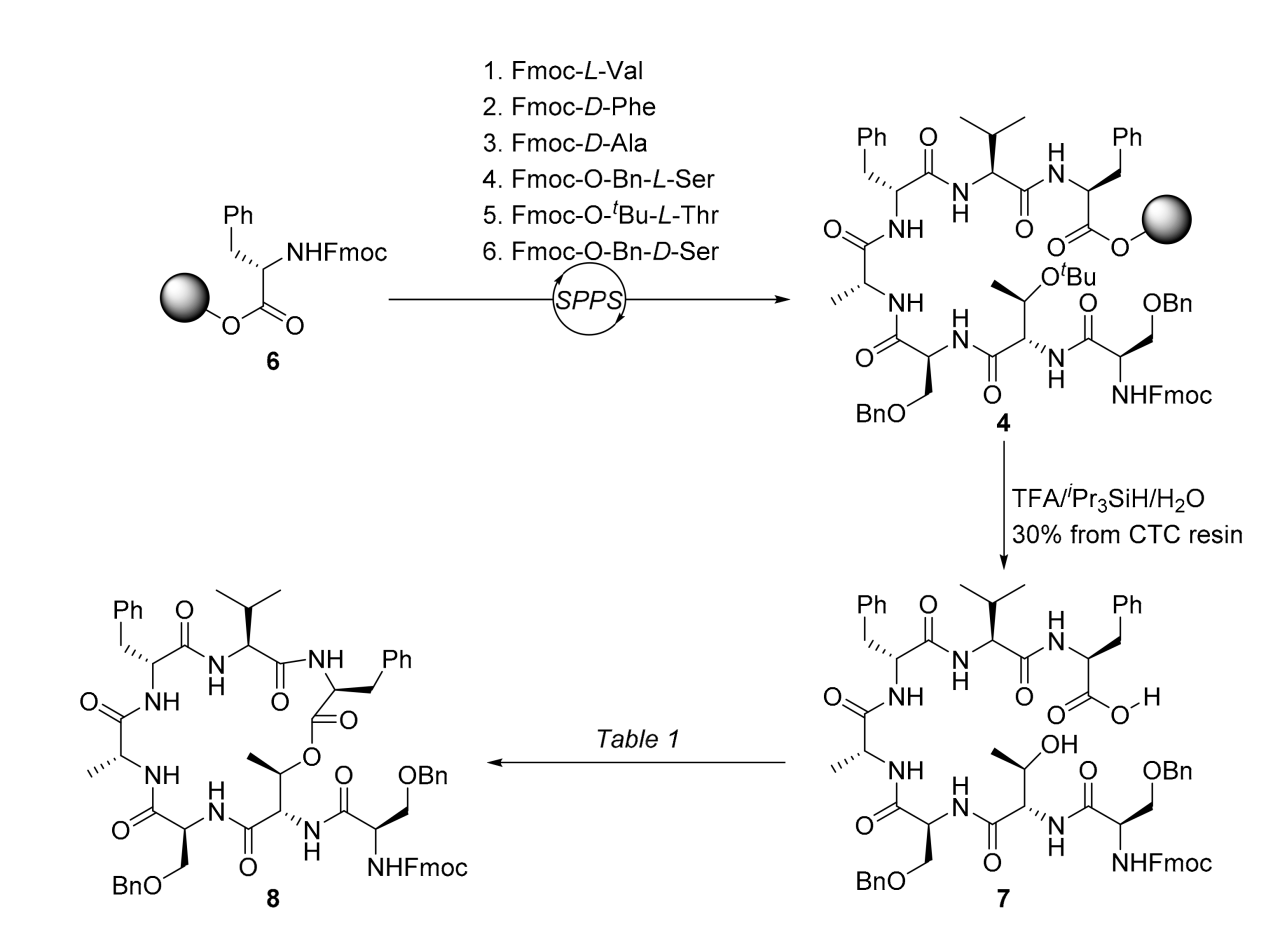
Solid-phase synthesis of intermediates **4**, **7** and **8** from resin-bound Fmoc-l-phenylalanine (**6**).

The synthetic campaign towards pagoamide A was initiated with 2-chlorotrityl chloride (CTC) resin-bound Fmoc-l-phenylalanine (**6**). Iterative peptide elongation was carried out under solid phase peptide synthesis conditions (Scheme 2). Fmoc-d-Val, Fmoc-d-Phe, Fmoc-d-Ala, Fmoc-*O*-Bn-l-Ser, Fmoc-*O*-*^t^*Bu-l-Thr and Fmoc-*O*-Bn-d-Ser were consecutively loaded onto the resin to provide the resin-immobilized heptapeptide **4**. Cleavage of the resin with concomitant deprotection of the *tert*-butyl ether was achieved in trifluoroacetic acid−triisopropylsilane−water (95:2.5:2.5). Thus, the seco-acid was obtained in an overall 30% yield starting from CTC resin and we were thus poised to investigate the pivotal macrolactonization.

As summarized in Table 1, an exhaustive screening of conditions was conducted. Thus, seco-acid **7** was treated with the Shiina reagent (2-methyl-6-nitrobenzoic anhydride, MNBA) [15] to effect the intramolecular esterification, giving rise to the macrocycle **8** but in a very low yield (20%, entry 1).

Disappointedly, macrolactonization with MNBA in different solvent systems (entries 2–3) only resulted in diminished yields. Other conditions (entries 4–10) employing 2,4,6-trichlorobenzoyl chloride (TCBC, Yamaguchi Reagent) [16], 2-bromo-1-ethylpyridinium tetrafluoroborate (BEP) [17], *N*-ethyl-*N’*-dimethylaminopropylcarbodiimide (EDCI), *N*,*N’*-dicyclohexylcarbodiimide (DCC) [18], and *N*,*N*,*N’*,*N’*-tetramethylchloroformamidinium hexafluorophosphate (TCFH) [19] all provided intractable mixtures with little or no desired products. These unsatisfactory results prompted us to explore a new approach wherein the difficult ester bond is installed at an earlier stage, and macrocyclization is performed by lactamization rather than lactonization. 

**Scheme 3:**
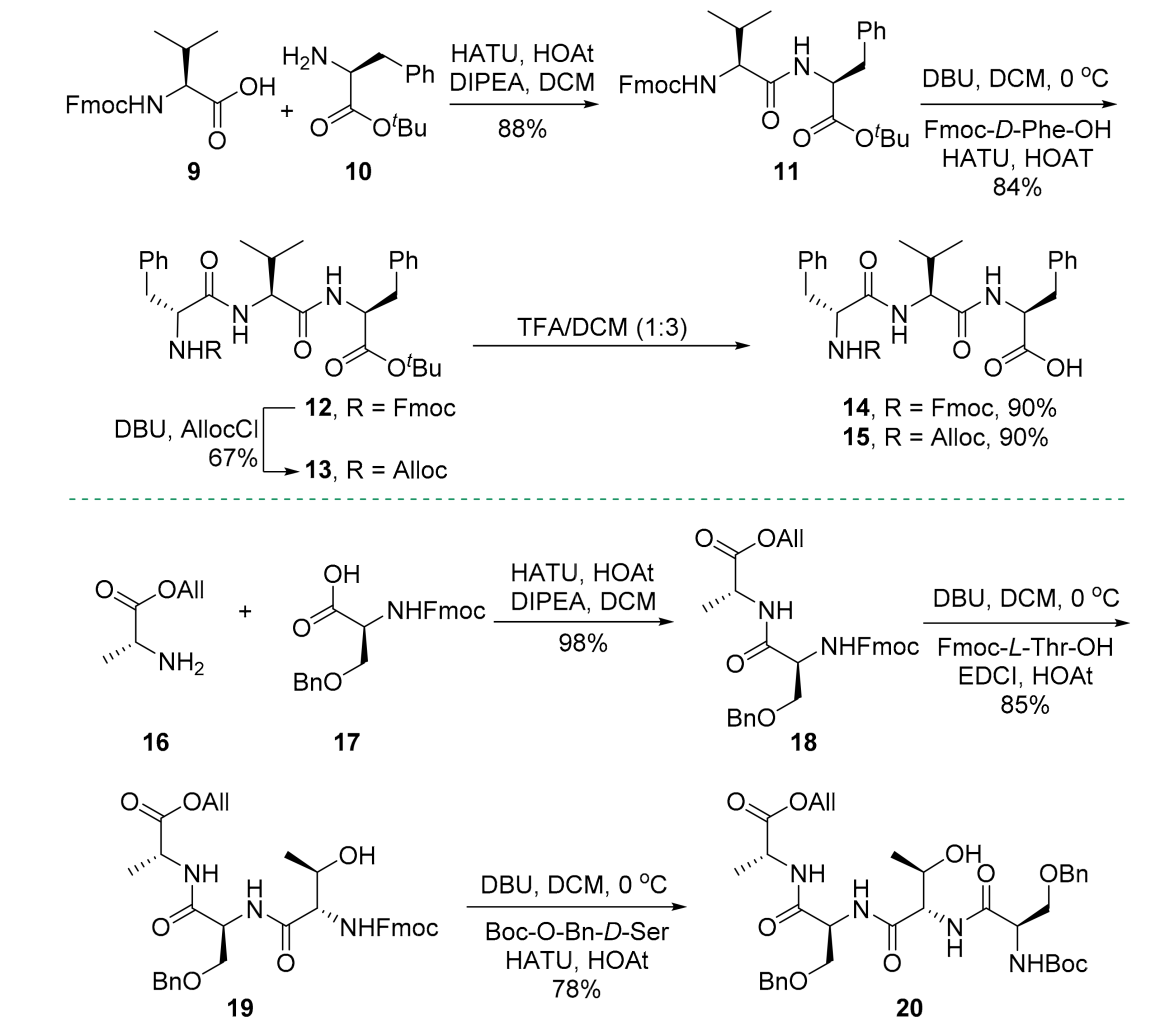
Synthesis of intermediates **11**–**15** and **18**–**20** from amino acids **9**, **10**, **16** and **17**.

Thus, condensation of Fmoc-l-Val-OH **9** and *O*-*^t^*Bu-l-Phe **10**, mediated by (1-[bis (dimethylamino)-methylene]-1H-1,2,3-triazolo[4,5-b]pyridinium 3-oxide hexafluorophosphate) (HATU), 1-hydroxy-7-azabenzotriazole (HOAt) and *N*,*N*-diisopropylethylamine (DIPEA) proceeded smoothly to afford dipeptide **11** in 88% yield (Scheme 3). Removal of Fmoc protecting group from the dipeptide **11** with 1,8-diazabicyclo [5.4.0]undec-7-ene (DBU) followed by coupling of the resulting amines with Fmoc-d-Phe-OH under HATU/HOAt conditions furnished tripeptide **12** in 84% yield [20]. Subsequently, functional group manipulations afforded the two desired tripeptidic acids **14** and **15** without incident. In parallel, coupling of *O*-All-l-Ala **16** with Fmoc-*O*-Bn-l-Ser-OH **17** was achieved through carboxyl activation with HATU/HOAt and provided dipeptide **18** in 98% yield. Then, applying the same sequence that was used for the synthesis of dipeptide **18**, tetrapeptide **20** was prepared uneventfully from **18** through incorporation of Fmoc-*O*-*^t^*Bu-l-Thr and Fmoc-*O*-Bn-d-Ser (Scheme 3).

**Scheme 4:**
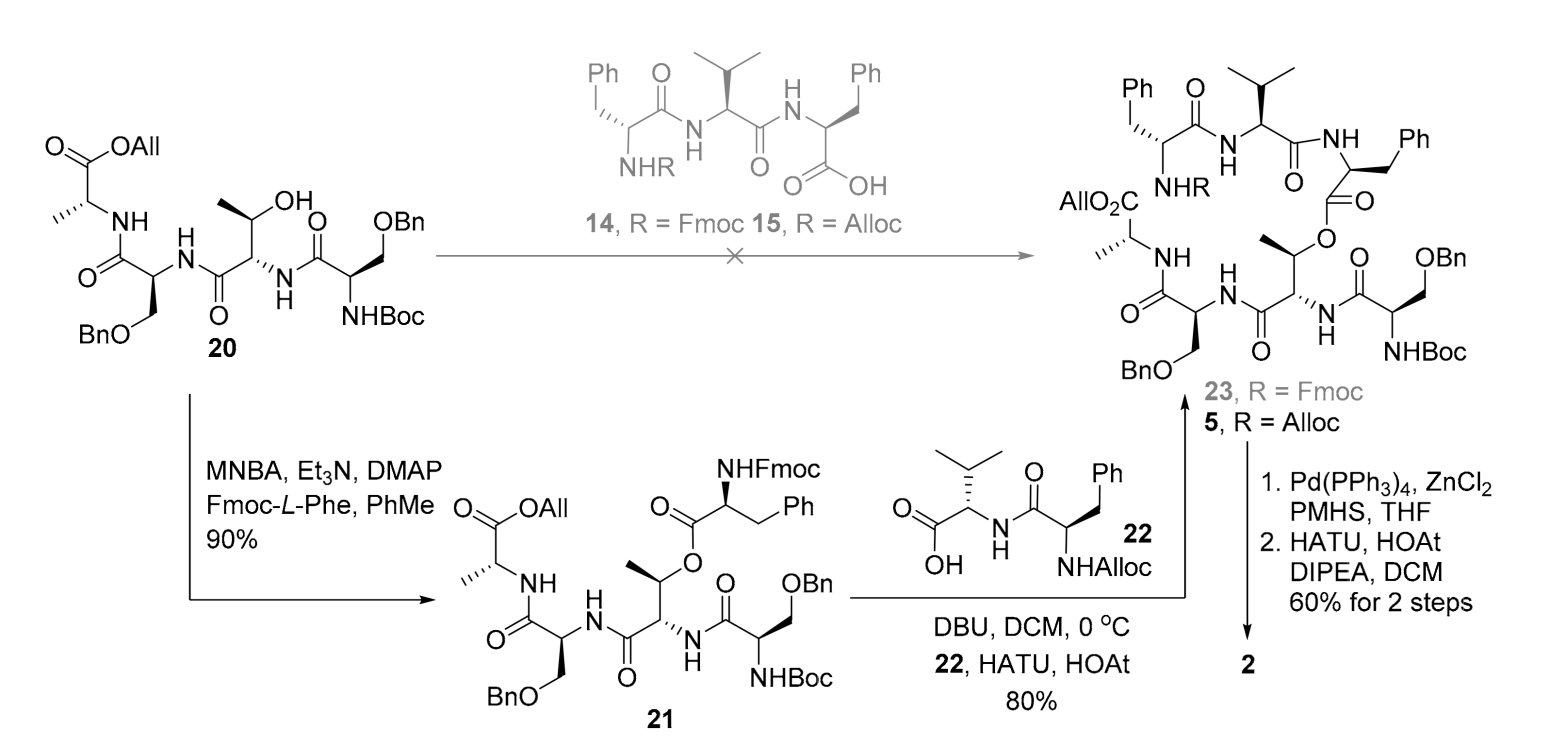
Synthesis of intermediates **21** and **2** from tetrapeptide **20**.

With the tetrapeptide **20** in hand, the stage was set for the assembly of the macrocycle (Scheme 4). Esterification of the alcohol **20** with acid **14** or **15** turned out to be much more challenging than expected. A plethora of reagents was examined to mediate the coupling between alcohol **20** and acids **14** or **15**. As monitored by TLC and LCMS, tetrapeptidic alcohol **20** remained intact and the acid **14/15** readily decomposed under the reaction conditions. We suspected that the formation of oxazolone upon activation of the acid might be one of the detrimental pathways. Therefore, esterification of alcohol **20** with Fmoc-l-Phe was attempted. To our delight, under Shiina conditions, ester **21** was obtained in 90% yield. Further condensation of dipeptide **22** with the free amine derived from fragment **21**, mediated by HATU/HOAt gave rise to macrocyclization precursor **5** in 80% yield. Simultaneous deprotection of the allyl ester and alloc protecting groups by using Pd(PPh_3_)_4_/ZnCl_2_/polymethylhydrosiloxane (PMHS) [21], and subsequent macrocyclization of the resulting amino acid produced macrocycle **2** in 60% yield over two steps.

**Scheme 5:**
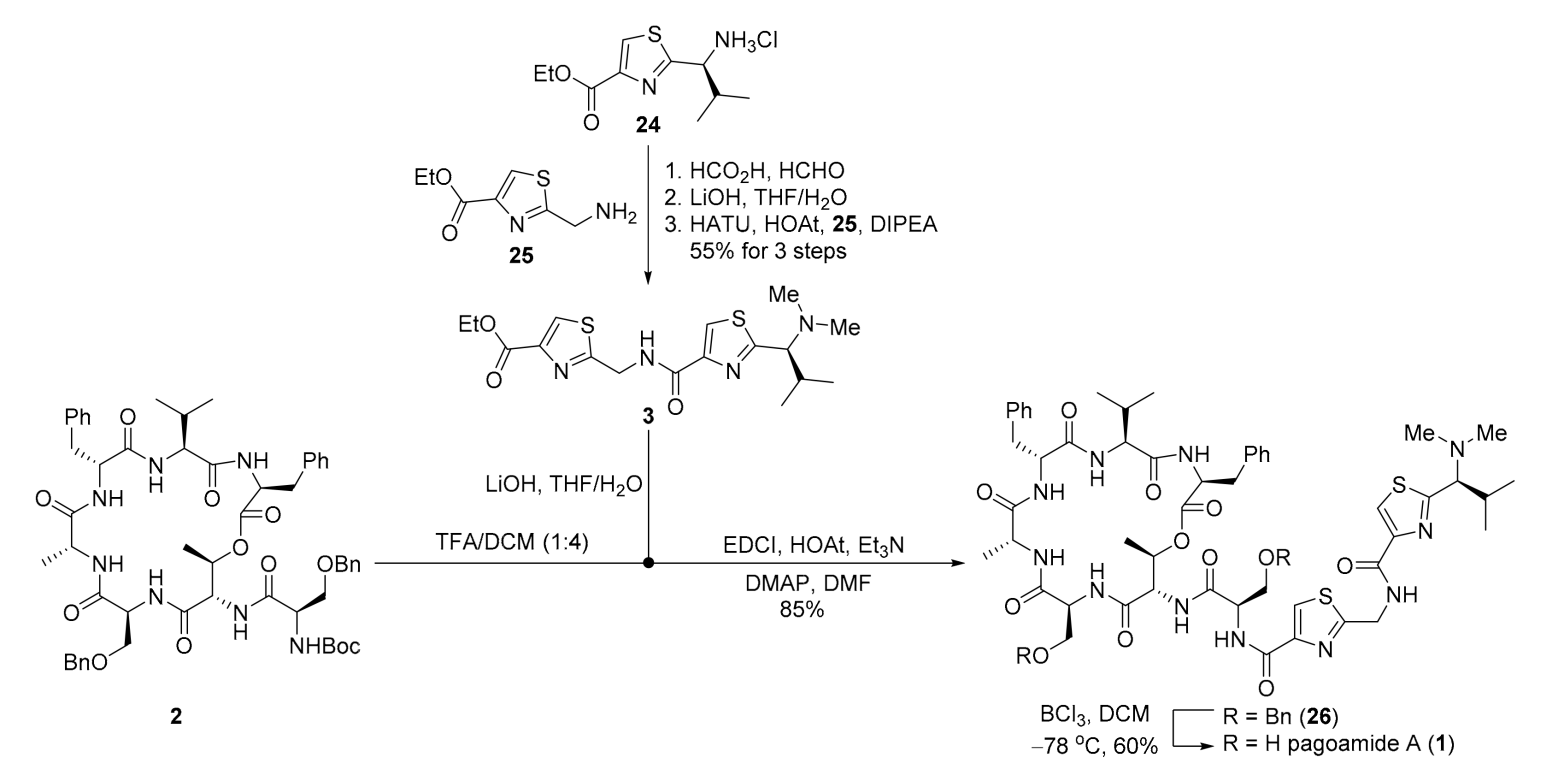
Total synthesis of pagoamide A (**1**).

The final stage was set for the incorporation of *bis*-thiazole fragment **3** (Scheme 5), which was prepared from the known valine-derived thiazole **24** [22] via a three-step sequence including: (1) reductive amination to install the *N*,*N*-dimethyl moiety; (2) saponification in THF/H_2_O with lithium hydroxide to release the corresponding acid; (3) amidation with the other thiazole subunit **25** [23]. Trifluoroacetic acid-promoted removal of the Boc group in **2** followed by coupling of the resulting amine with the acid derived from ethyl ester **3** in the presence of EDCI/HOAt/TEA/DMAP in DMF delivered the corresponding depsipeptide **26** in 85% yield. Global deprotection was achieved by employing boron trichloride in DCM at −78 °C and furnished pagoamide A (**1**) in 60% yield. With many building blocks already in hand, the next step was to prepare the diastereoisomer **1a**, and this was readily achieved by following the same synthetic procedure as for pagoamide A (**1**). The synthesis proceeded smoothly under the previous conditions through cyclization and attachment of the thiazole-containing side chain (See Supplementary Materials for details). The optical rotation of the synthetic product **1** $[{\alpha ]\;}_{D}^{25}\;$= +8.0 (*c* 0.1, MeOH), was in close agreement with the value reported in the literature for natural pagoamide A $[{\alpha ]\;}_{D}^{25}\;$= +5.5 (*c* 0.1, MeOH). Spectral data, including ^1^H-NMR, ^13^C-NMR, were collected for both the natural and synthetic sample (**1**) and found to be in full agreement (Table 2) [1], while the synthetic **1a** showed remarkable discrepancies in spectral data as compared to natural pagoamide A, which unambiguously confirmed the original assignments of natural pagoamide A.

## 3. Materials and Methods

### 3.1. General Information

All reactions were conducted in flame-dried or oven-dried glassware under an atmosphere of dry nitrogen or argon. Oxygen and/or moisture-sensitive solids and liquids were transferred appropriately. The concentration of solutions in vacuo was accomplished using a rotary evaporator fitted with a water aspirator. Residual solvents were removed under a high vacuum (0.1–0.2 mm Hg). All reaction solvents were purified before use: tetrahydrofuran (THF) was distilled from Na/benzophenone. Toluene was distilled over molten sodium metal. Dichloromethane (DCM), 1,2-dichloroethane (DCE) and trimethylamine (Et_3_N) were distilled from CaH_2_. Methanol (MeOH) was distilled from Mg/I_2_. The reagents were purchased at the highest commercial quality and used without further purification unless otherwise stated. Flash column chromatography was performed using the indicated solvents on silica gel 60 (230–400 mesh ASTM E. Qingdao, Tsingtao, China). Reactions were monitored using thin-layer chromatography (TLC), which was carried out using pre-coated sheets (Qingdao silica gel 60-F250, 0.2 mm). Compounds were visualised with UV light, iodine and ceric ammonium molybdate stainer phosphomolybdic acid in EtOH. The ^1^H NMR spectra were recorded on Avance 300 MHz, Avance 400 MHz or Avance 500 MHz spectrometers (Bruker, Karlsruhe, Germany). Chemical shifts were reported in parts per million (ppm), relative to either a tetramethylsilane (TMS) internal standard or the signals due to the solvent. The following abbreviations are used to describe the spin multiplicity: s = singlet, d = doublet, t = triplet, q = quartet, qn = quintet, m = multiplet, br = broad, dd = doublet of doublets, dt = doublet of triplets, dq = doublet of quartets, ddd = doublet of doublet of doublets; other combinations are derived from those listed above. Coupling constants (*J*) are reported in Hertz (Hz) for corresponding solutions, and chemical shifts are reported as parts per million (ppm) relative to residual CHCl_3_ δH (7.26 ppm). ^13^C-NMR nuclear magnetic resonance spectra were recorded at 75 MHz, 100 MHz or 125 MHz for corresponding solutions, and chemical shifts are reported as parts per million (ppm) relative to residual CDCl_3_ δC (77.16 ppm). High-resolution mass spectra were measured on an ABI Q-star Elite (Beijing, China). Optical rotations were recorded on a Rudolph AutoPol-I polarimeter (Shanghai, China) at 589 nm with a 50 mm cell. Data are reported as follows: specific rotation (*c* (g/100 mL), solvent).

### 3.2. General Experimental Procedures

#### 3.2.1. *N*-*N*-(((9H-fluoren-9-yl)methoxy)carbonyl)-*O*-benzyl-d-seryl-l-threonyl-*O*-benzyl-l-seryl-d-alanyl-d-phenylalanyl-l-valyl-l-phenylalanine (**7**)

(A) Fmoc-based Solid-phase Peptide Synthesis (SPPS)

The linear peptides were prepared by manual SPPS using 2-chlorotrityl resin (GL Biochem, loading: ca. 1.0 mmol/g) and the general procedure was stated as follows: Anhydrous DCM was employed to swell the resin in a suitable syringe for half an hour. Subsequently, the swollen resin was washed with DCM (3 × 30 mL), and a solution of Fmoc-l-Phe (5.0 equiv. to the resin capacity) and DIPEA (10.0 equiv. to the resin capacity) in DMF was added to this reaction vessel. The reaction mixture was shaken at room temperature for 8 h. After that, the resin was washed with DCM (3 × 30 mL) and DMF (3 × 30 mL), respectively. Next, the resin underwent iterative peptide assembly (Fmoc-SPPS). A solution of Fmoc-protected amino acid (4.0 equiv. according to the resin capacity), HATU (4.0 equiv.) and DIPEA (8.0 equiv.) in DMF was gently agitated with the resin at room temperature for 2 h. Subsequently, the resin was washed with DCM (3 × 30 mL) and DMF (3 × 30 mL). After Kaiser test showed complete coupling, the deprotection of Fmoc group at *N*-terminus was achieved by 30 min treatment of 50% morpholine in DMF at room temperature. As followed, the resin was washed thoroughly with DCM (3 × 30 mL) and DMF (3 × 30 mL). The coupling and deprotecting procedures were repeated for coupling each amino acid until the desired linear peptide sequence **4** was obtained.

(B) Removal of the C-terminal protecting group

The on-resin fully protected peptidyl acid **4**, obtained as described in the previous procedure A, was subjected to TFA/TIPSH/H_2_O (95/2.5/2.5, *v*/*v*/*v*) for 2 h. Following filtration, the resulting solutions were combined and concentrated to give the crude protected peptide bearing the free carboxylic acid at the C-terminus. The crude acid was purified by flash column chromatography on silica gel (MeOH/DCM = 1/20) to afford **7** as a white solid. TLC: R*_f_* = 0.6 (MeOH/DCM = 1/10), UV & PMA stain, $\alpha_{D}^{23}$ = +2.0 (c 0.7, DMSO). ^1^H- NMR (300 MHz, DMSO-*d*_6_) δ 8.31 (d, *J* = 7.6 Hz, ^1^H), 8.14 (d, *J* = 7.6 Hz, ^1^H), 8.05 (d, *J* = 8.1 Hz, ^1^H), 8.03–7.93 (m, ^2^H), 7.87 (d, *J* = 7.6 Hz, ^2^H), 7.81 (d, *J* = 8.5 Hz, ^1^H), 7.71 (d, *J* = 7.7 Hz, ^2^H), 7.66 (d, *J* = 4.5 Hz, ^1^H), 7.39 (t, *J* = 7.5 Hz, ^2^H), 7.32–7.25 (m, ^10^H), 7.25–7.21 (m, ^6^H), 7.20–7.16 (m, ^4^H), 7.16–7.10 (m, ^2^H), 4.62–4.48 (m, ^2^H), 4.51–4.33 (m, ^10^H), 4.30–4.20 (m, ^3^H), 4.24–4.10 (m, ^4^H), 3.03 (dd, *J* = 13.9, 5.5 Hz, ^1^H), 2.98–2.92 (m, ^1^H), 2.92–2.84 (m, ^1^H), 2.82–2.67 (m, ^1^H), 2.09–1.78 (m, ^1^H), 1.09 (d, *J* = 7.0 Hz, ^3^H), 0.98 (d, *J* = 6.4 Hz, ^3^H), 0.70 (d, *J* = 6.8 Hz, ^3^H), 0.66 (d, *J* = 6.6 Hz, ^3^H). ^13^C-NMR (75 MHz, DMSO-*d*_6_) δ 173.0, 172.0, 171.2, 170.9, 170.2, 170.0, 169.2, 158.9, 158.4, 156.3, 144.1, 143.9, 140.9, 138.3, 138.3, 137.7, 129.4, 129.3, 128.4, 128.3, 127.9, 127.8, 127.7, 127.4, 126.7, 126.5, 125.5, 120.3, 72.3, 69.7, 66.8, 66.2, 57.8, 57.4, 55.1, 54.9, 54.4, 53.8, 53.2, 48.5, 46.8, 36.8, 30.9, 19.4, 19.3, 18.4, 17.8. HRMS (ESI) calculated for C_65_H_73_N_7_O_13_Na^+^ [M + Na]^+^ 1182.5159, found 1182.5153.

#### 3.2.2. (9*H*-Fluoren-9-yl)methyl ((*R*)-3-(benzyloxy)-1-(((3S,6S,9R,12R,15S,18S,19R)-3,9-dibenzyl-15-((benzyloxy)methyl)-6-isopropyl-12,19-dimethyl-2,5,8,11,14,17-hexaoxo-1-oxa-4,7,10,13,16-pentaazacyclononadecan-18-yl)amino)-1-oxopropan-2-yl)carbamate (**8**)

Compound **7** was treated with different macrolactonization conditions, as showed in Supplementary Materials. The experimental procedure (entry 1) was as follows: To a solution of **7** (50 mg, 0.043 mmol, 1.0 eq.) and DMAP (27 mg, 0.22 mmol, 5.0 eq.) in anhydrous DCM (50 mL, ca. 0.001 M) was added MNBA (45 mg, 0.13 mmol, 3.0 eq.) under an argon atmosphere at 0 ^°^C. The mixture was allowed to stir at room temperature for 12 h and then concentrated in vacuo furnishing a solid residue which was redissolved in EtOAc (5 mL) and quenched with saturated aqueous solution of NH_4_Cl (2 mL). The aqueous layer was extracted with EtOAc (3 × 10 mL). The combined organic layers were washed with brine (5 mL), dried over anhydrous Na_2_SO_4_, filtered, and concentrated under reduced pressure. Purification of the crude product was performed by flash chromatography on silica gel (hexanes/EtOAc = 2/1-1/1) to afford 8 (10 mg, 20%) as a white solid. TLC: R*_f_* = 0.4 (hexanes/EtOAc = 1/1), UV & PMA stain. $\alpha_{D}^{24}$= +18.2 (*c* 8.4, CHCl_3_). ^1^H-NMR (400 MHz, CDCl_3_) δ 7.87 (d, *J* = 9.5 Hz, ^1^H), 7.82 (d, *J* = 8.7 Hz, ^1^H), 7.73 (d, *J* = 7.6 Hz, ^2^H), 7.52 (d, *J* = 7.5 Hz, ^2^H), 7.39–7.31 (m, ^4^H), 7.33–7.25 (m, ^7^H), 7.28–7.20 (m, ^6^H), 7.20–7.16 (m, ^4^H), 7.15–7.11 (m, ^4^H), 7.07 (d, *J* = 7.2 Hz, ^1^H), 6.82 (d, J = 10.0 Hz, ^1^H), 6.66–6.54 (m, ^2^H), 6.11 (d, *J* = 8.3 Hz, ^1^H), 5.67–5.57 (m, ^1^H), 5.28 (td, *J* = 10.8, 4.5 Hz, ^1^H), 5.04 (ddd, *J* = 13.3, 8.9, 3.7 Hz, ^2^H), 4.75–4.55 (m, ^3^H), 4.52–4.47 (m, ^2^H), 4.29–4.16 (m, ^2^H), 4.20–4.11 (m, ^2^H), 4.13–4.03 (m, ^2^H), 3.97 (dd, *J* = 10.0, 4.4 Hz, ^1^H), 3.85–3.74 (m, ^1^H), 3.74–3.50 (m, ^2^H), 3.40 (dd, *J* = 14.2, 4.6 Hz, ^1^H), 3.32 (d, *J* = 8.0 Hz, ^2^H), 2.98 (dd, *J* = 14.2, 11.6 Hz, ^1^H), 2.12–2.01 (m, ^1^H), 1.17 (dd, *J* = 6.8, 4.3 Hz, ^6^H), 0.73 (d, *J* = 6.9 Hz, ^3^H), 0.32 (d, *J* = 6.9 Hz, ^3^H). ^13^C- NMR (100 MHz, CDCl_3_) δ 173.6, 173.4, 172.0, 171.5, 170.8, 170.7, 169.8, 156.0, 144.3, 143.8, 141.3, 141.3, 137.8, 137.6, 136.9, 129.5, 129.1, 128.8, 128.5, 128.5, 128.4, 128.4, 128.0, 128.0, 127.8, 127.7, 127.1, 126.5, 126.5, 125.4, 125.3, 120.0, 73.8, 73.2, 71.0, 70.2, 68.0, 67.2, 59.8, 55.9, 55.4, 54.8, 52.5, 52.3, 51.5, 47.2, 36.5, 33.9, 29.6, 19.7, 16.9, 16.7, 16.3. HRMS (ESI) calculated for C_65_H_72_N_7_O_12_^+^ [M + H]^+^ 1142.5233, found 1142.5228.

#### 3.2.3. *tert-*Butyl (((9*H*-fluoren-9-yl)methoxy)carbonyl)-l-valyl-l-phenylalaninate (**11**)

To a solution of amine 10 (3.0 g, 13.6 mmol, 1.0 eq.), Fmoc-l-Val 9 (6.9 g, 20.3 mmol, 1.5 eq.), HOAt (3.7 g, 27.2 mmol, 2.0 eq.) and DIPEA (9.0 mL, 54.2 mmol, 4.0 eq.) in dry DCM (100 mL, 0.14 M) under argon atmosphere, was added HATU (10.3 g, 27.2 mmol, 2.0 eq.) at 0 °C. The mixture was allowed to stir for 12 h at room temperature and then concentrated in *vacuo* furnishing a solid residue. The solid residue was redissolved in EtOAc (100 mL) and quenched with 4% citric acid aqueous solution. The aqueous layer was extracted with EtOAc (3 × 100 mL). The combined organic layers were washed with saturated aqueous solution of NaHCO_3_ (100 mL), brine (100 mL), dried over anhydrous Na_2_SO_4_, filtered and concentrated in vacuo. Purification of the crude product was performed by flash chromatography on silica (hexanes/EtOAc = 5/1) to afford 11 (6.5 g, 88%) as a white solid. TLC: R*_f_* = 0.4 (hexanes/EtOAc = 4/1), UV & PMA stain. $\alpha_{D}^{25}$ = +6.2 (*c* 2.0, CHCl_3_). ^1^H-NMR (400 MHz, CDCl_3_) δ 7.77 (dd, *J* = 7.5, 1.0 Hz, ^2^H), 7.60 (d, *J* = 7.5 Hz, ^2^H), 7.44–7.36 (m, ^2^H), 7.36–7.28 (m, ^2^H), 7.25–7.22 (m, ^2^H), 7.22–7.16 (m, ^1^H), 7.16–7.09 (m, ^2^H), 6.21 (d, *J* = 7.8 Hz, ^1^H), 5.36 (d, *J* = 8.9 Hz, ^1^H), 4.75 (dt, *J* = 7.8, 6.1 Hz, ^1^H), 4.44 (dd, *J* = 10.5, 7.1 Hz, ^1^H), 4.35 (dd, *J* = 10.6, 7.0 Hz, ^1^H), 4.23 (t, *J* = 7.0 Hz, ^1^H), 4.05–3.89 (m, ^1^H), 3.08 (d, *J* = 6.1 Hz, ^2^H), 2.31–2.03 (m, ^1^H), 0.94 (d, *J* = 6.7 Hz, ^3^H), 0.91 (d, *J* = 6.8 Hz, ^3^H). ^13^C-NMR (100 MHz, CDCl_3_) δ 170.7, 170.4, 156.4, 144.0, 144.0, 141.5, 136.0, 129.6, 128.6, 127.9, 127.2, 127.2, 125.3, 125.2, 120.1, 82.6, 67.2, 60.4, 53.7, 47.4, 38.3, 31.4, 28.1, 19.2, 17.9. HRMS (ESI) calculated for C_33_H_38_N_2_O_5_Na^+^ [M + Na]^+^ 565.2673, found 565.2671.

#### 3.2.4. *tert*-Butyl (((9*H*-fluoren-9-yl)methoxy)carbonyl)-d-phenylalanyl-l-valyl-l-phenylalaninate (**12**)

To a solution of 11 (1.5 g, 2.8 mmol, 1.0 eq.) in dry DCM (50 mL, 0.05 M) was added DBU (2.8 mL, 2.8 mmol, 1.0 eq., 1.0 M in DCM) at 0 ^°^C under argon atmosphere. The reaction mixture was stirred for 1.5 h at 0 °C. After full conversion of the starting material as determined by TLC, HOAt (750 mg, 5.5 mmol, 2.0 eq.) and Fmoc-d-Phe (1.1 g, 2.8 mmol, 1.0 eq.) were added at 0 °C, and the mixture was stirred for 5 min at the same temperature. Then, HATU (2.1 g, 5.5 mmol, 2.0 eq.) was added at 0 °C and the resulting mixture was allowed to stir for 12 h at room temperature. The reaction mixture was then quenched by the addition of H_2_O (20 mL). Layers were separated and the aqueous phase was extracted with EtOAc (3 × 50 mL). The combined organic layers were washed with brine (50 mL), dried over anhydrous Na_2_SO_4_, filtered and concentrated in vacuo. Purification of the crude product was performed by flash chromatography on silica gel (Hexanes/EtOAc = 3/1-2/1) to afford 12 (1.6 g, 84%) as a white solid. TLC: R*_f_* = 0.6 (hexanes/EtOAc = 2/1), UV & PMA stain. $\alpha_{D}^{26}$ = +10.0 (*c* 1.3, CHCl_3_). ^1^H-NMR (500 MHz, CDCl_3_) δ 7.75 (d, *J* = 7.3 Hz, ^2^H), 7.53 (dd, *J* = 13.4, 7.5 Hz, ^2^H), 7.39 (t, *J* = 7.5 Hz, ^2^H), 7.31–7.24 (m, ^5^H), 7.24–7.20 (m, ^4^H), 7.17 (t, *J* = 7.2 Hz, ^1^H), 7.14–7.10 (m, ^2^H), 6.50 (s, ^1^H), 6.43 (s, ^1^H), 5.52 (s, ^1^H), 4.70 (dt, *J* = 8.2, 6.5 Hz, ^1^H), 4.49–4.35 (m, ^2^H), 4.31–4.26 (m, ^1^H), 4.25–4.13 (m, ^2^H), 3.04 (dd, *J* = 6.5, 2.6 Hz, ^2^H), 2.03–1.96 (m, ^1^H), 1.36 (s, ^9^H), 0.74 (d, *J* = 6.7 Hz, ^3^H), 0.67 (d, *J* = 6.8 Hz, ^3^H). ^13^C-NMR (125 MHz, CDCl_3_) δ 170.9, 170.3, 170.2, 156.1, 143.8, 143.7, 141.3, 136.3, 136.1, 129.5, 129.3, 128.8, 128.4, 127.7, 127.2, 127.1, 127.0, 125.1, 125.1, 120.0, 82.4, 67.3, 58.5, 56.7, 53.7, 47.1, 38.6, 38.1, 30.7, 27.9, 18.9, 17.7. HRMS (ESI) calculated for C_42_H_47_N_3_O_6_Na^+^ [M + Na]^+^ 712.3357, found 712.3355.

#### 3.2.5. *tert*-Butyl ((allyloxy)carbonyl)-d-phenylalanyl-l-valyl-l-phenylalaninate (**13**)

To a solution of 12 (690 mg, 1.0 mmol, 1.0 eq.) in dry DCM (10 mL, 0.09 M) was added DBU (1.0 mL, 1.0 mmol, 1.0 eq., 1.0 M in DCM) at 0 °C under argon atmosphere. The reaction mixture was stirred for 1.5 h at 0 °C. After full conversion of the starting material as determined by TLC, AllocCl (0.1 mL 1.0 mmol, 1.0 eq.) was added at 0 °C, and the mixture was stirred for 2 h at the same temperature. The reaction mixture was quenched by the addition of H_2_O (10 mL). Layers were separated and the aqueous phase was extracted with EtOAc (3 × 10 mL). The combined organic layers were washed with brine (20 mL), dried over anhydrous Na_2_SO_4_, filtered and concentrated in vacuo. Purification of the crude product was performed by flash chromatography on silica gel (Hexanes/EtOAc = 4/1) to afford 13 (369 mg, 67%) as a white solid. TLC: R*_f_* = 0.6 (hexanes/EtOAc = 10/1), iodine and PMA stain. $\alpha_{D}^{25}\;$= +9.6 (*c* 1.0, CHCl_3_). ^1^H-NMR (500 MHz, CDCl_3_) δ 7.28–7.17 (m, 8H), 7.16–7.13 (m, ^2^H), 7.07–6.92 (m, ^1^H), 6.82–6.67 (m, ^1^H), 5.94–5.74 (m, ^1^H), 5.27–5.19 (m, ^1^H), 5.17–5.12 (m, ^1^H), 4.80–4.68 (m, ^1^H), 4.56–4.44 (m, ^3^H), 4.39–4.31 (m, ^1^H), 3.10 (dd, *J* = 13.8, 7.4 Hz, ^1^H), 3.02 (d, *J* = 6.7 Hz, ^3^H), 2.00–1.90 (m, ^1^H), 1.36 (s, ^9^H), 0.75 (d, *J* = 6.7 Hz, ^3^H), 0.68 (d, *J* = 6.8 Hz, ^3^H). ^13^C-NMR (125 MHz, CDCl_3_) δ 171.2, 170.5, 170.5, 156.0, 136.5, 136.3, 132.7, 129.5, 129.4, 128.7, 128.4, 127.1, 127.0, 117.9, 82.3, 66.0, 58.4, 56.7, 53.9, 38.9, 38.3, 30.9, 28.0, 19.0, 17.9. HRMS (ESI) calculated for C_31_H_41_N_3_O_6_Na^+^ [M + Na]^+^ 574.2888, found 574.2888.

#### 3.2.6. (((9*H*-Fluoren-9-yl)methoxy)carbonyl)-d-phenylalanyl-l-valyl-l-phenylalanine (**14**)

To a solution of 12 (1.0 g, 1.5 mmol, 1.0 eq.) in DCM (15 mL, 0.075 M) was added TFA (5 mL) dropwise at 0 °C. After being stirred at room temperature for 10 h, the reaction mixture was concentrated in vacuo. Purification of the crude product was performed by flash chromatography on silica gel (MeOH/DCM = 1/20) to afford 14 (855 mg, 90%) as a white solid. TLC: R*_f_* = 0.6 (MeOH/DCM = 1/10), UV & PMA stain. $\alpha_{D}^{26}\;$= +6.3 (*c* 1.0, DMSO). ^1^H-NMR (400 MHz, DMSO-*d*_6_) δ 8.17 (d, *J* = 7.8 Hz, ^1^H), 8.10 (d, *J* = 9.1 Hz, ^1^H), 7.86 (d, *J* = 7.7 Hz, ^2^H), 7.75 (d, *J* = 8.7 Hz, ^1^H), 7.64 (t, *J* = 7.2 Hz, ^2^H), 7.39 (t, *J* = 7.7 Hz, ^2^H), 7.33 (d, *J* = 7.9 Hz, ^2^H), 7.30–7.13 (m, ^9^H), 7.12–7.01 (m, ^1^H), 4.46–4.33 (m, ^2^H), 4.19 (t, *J* = 7.4 Hz, ^1^H), 4.12 (s, ^3^H), 3.07 (dd, *J* = 13.9, 5.1 Hz, ^1^H), 3.01–2.89 (m, ^2^H), 2.86–2.75 (m, ^1^H), 2.07–1.83 (m, ^1^H), 0.78–0.59 (m, ^6^H). ^13^C-NMR (100 MHz, DMSO-*d*_6_) δ 173.1, 171.5, 170.7, 155.9, 143.8, 143.7, 140.7, 138.0, 137.9, 129.4, 129.2, 128.1, 128.1, 127.7, 127.1, 126.3, 126.2, 125.4, 125.4, 120.1, 65.0, 57.5, 56.5, 54.0, 46.6, 37.9, 36.9, 30.7, 19.2, 17.8. HRMS (ESI) calculated for C_38_H_39_N_3_O_6_Na^+^ [M + Na]^+^ 656.2731, found 656.2730.

#### 3.2.7. ((Allyloxy)carbonyl)-d-phenylalanyl-l-valyl-l-phenylalanine (**15**)

Compound **15** was synthesized according to the procedures for the synthesis of **14** from **13** (369 mg, 0.67 mmol, 1.0 eq.). Purification of the crude product was performed by flash chromatography on silica gel (MeOH/DCM = 1/40) to afford 15 (298 mg, 90%) as a white solid. TLC: R*_f_* = 0.5 (MeOH/DCM = 1/20), UV & PMA stain. $\alpha_{D}^{27}$ = −3.7 (*c* 4.38, DMSO). ^1^H-NMR (400 MHz, DMSO-*d*_6_) δ 8.28 (d, *J* = 7.6 Hz, ^1^H), 7.96 (d, *J* = 8.9 Hz, ^1^H), 7.45 (d, *J* = 8.5 Hz, ^1^H), 7.34–7.28 (m, ^2^H), 7.30–7.20 (m, ^6^H), 7.20–7.13 (m, ^2^H), 5.82 (ddt, *J* = 16.0, 10.4, 5.2 Hz, ^1^H), 5.26–5.16 (m, ^1^H), 5.11 (d, *J* = 10.5 Hz, ^1^H), 4.46–4.39 (m, ^1^H), 4.39–4.31 (m, ^3^H), 4.29–4.17 (m, ^1^H), 3.05 (dd, *J* = 14.0, 5.5 Hz, ^1^H), 2.98–2.87 (m, ^2^H), 2.73 (dd, *J* = 13.6, 10.4 Hz, ^1^H), 2.06–1.86 (m, ^1^H), 0.76 (d, *J* = 6.7 Hz, ^3^H), 0.72 (d, *J* = 6.8 Hz, ^3^H). ^13^C- NMR (100 MHz, DMSO-*d*_6_) δ 172.8, 171.5, 170.9, 155.7, 137.9, 137.5, 133.5, 129.3, 129.1, 128.2, 128.0, 126.4, 126.3, 116.9, 64.4, 57.1, 56.2, 53.5, 37.8, 36.6, 30.8, 19.1, 17.6. HRMS (ESI) calculated for C_27_H_33_N_3_O_6_Na^+^ [M + Na]^+^ 518.2262, found 518.2258.

#### 3.2.8. Allyl *N*-(((9*H*-fluoren-9-yl)methoxy)carbonyl)-*O*-benzyl-l-seryl-d-alaninate (**18**)

To a solution of amine **16** (6.4 g, 49.4 mmol, 2.0 eq.), acid **17** (10.0 g, 24.7 mmol, 1.0 eq.), HOAt (6.8 g, 49.4 mmol, 2.0 eq.) and DIPEA (17.0 mL, 98.8 mmol, 4.0 eq.) in dry DCM (300 mL, 0.08 M) under argon atmosphere, was added HATU (18.8 g, 49.4 mmol, 2.0 eq.) at 0 °C. The mixture was allowed to stir for 12 h at room temperature and then concentrated in vacuo furnishing a solid residue. The solid residue was redissolved in EtOAc (300 mL) and quenched with 4% citric acid aqueous solution. The aqueous layer was extracted with EtOAc (3 × 300 mL). The combined organic layers were washed with saturated aqueous solution of NaHCO_3_ (300 mL), brine (300 mL), dried over anhydrous Na_2_SO_4_, filtered and concentrated in vacuo. Purification of the crude product was performed by flash chromatography on silica gel (hexanes/EtOAc = 4/1) to afford 18 (12.8 g, 98%) as a white solid. TLC: R*_f_* = 0.5 (hexanes/EtOAc = 3/1), UV & PMA stain. $\alpha_{D}^{24}$ = +6.7 (*c* 2.0, CHCl_3_). ^1^H-NMR (500 MHz, CDCl_3_) δ 7.77 (d, *J* = 7.5 Hz, 2H), 7.59 (d, *J* = 7.5 Hz, 2H), 7.40 (t, *J* = 7.5 Hz, 2H), 7.39–7.33 (m, 1H), 7.36–7.26 (m, 6H), 6.97 (d, *J* = 7.0 Hz, 1H), 5.89 (ddt, *J* = 16.5, 11.0, 5.8 Hz, 1H), 5.77–5.59 (m, 1H), 5.37–5.28 (m, 1H), 5.29–5.22 (m, 1H), 4.71–4.56 (m, 4H), 4.53 (d, *J* = 11.8 Hz, 1H), 4.46–4.36 (m, 3H), 4.22 (t, *J* = 7.0 Hz, 1H), 3.91 (s, 1H), 3.69–3.49 (m, 1H), 1.40 (d, *J* = 7.2 Hz, 3H). ^13^C-NMR (125 MHz, CDCl_3_) δ 172.3, 169.5, 156.3, 143.9, 143.8, 141.4, 137.4, 131.6, 128.6, 128.1, 128.0, 127.9, 127.9, 127.2, 125.2, 120.1, 118.9, 73.6, 69.7, 67.4, 66.1, 54.3, 48.5, 47.3, 18.4. HRMS (ESI) calculated for C_31_H_32_N_2_O_6_Na^+^ [M + Na]^+^ 551.2153, found 551.2150.

#### 3.2.9. Allyl *N*-((((9H-fluoren-9-yl)methoxy)carbonyl)-l-threonyl)-*O*-benzyl-l-seryl-d-alaninate (**19**)

To a solution of **18** (3.0 g, 5.8 mmol, 1.0 eq.) in dry DCM (100 mL, 0.05 M) was added DBU (5.8 mL, 5.8 mmol, 1.0 eq., 1.0 M in DCM) at 0 °C under argon atmosphere. The reaction mixture was stirred for 1.5 h at 0 °C. After full conversion of the starting material as determined by TLC, HOAt (1.6 g, 11.6 mmol, 2.0 eq.) and Fmoc-l-Thr (2.0 g, 11.6mmol, 1.0 eq.) were added at 0 °C, and the mixture was stirred for 5 min at the same temperature. Then, EDCI (2.2 g, 11.6 mmol, 2.0 eq.) was added at 0 °C and the resulting mixture was allowed to stir for 12 h at room temperature. The reaction mixture was then quenched by the addition of H_2_O (100 mL). Layers were separated and the aqueous phase was extracted with EtOAc (3 × 100 mL). The combined organic layers were washed with brine (100 mL), dried over anhydrous Na_2_SO_4_, filtered and concentrated in vacuo. Purification of the crude product was performed by flash chromatography on silica gel (MeOH/DCM = 1/60) to afford 19 (3.1 g, 85%) as a white solid. TLC: R_f_ = 0.7 (MeOH/DCM = 1/20), UV & PMA stain.${\;\alpha }_{D}^{26}\;$= +2.6 (c 1.3, DMSO). ^1^H-NMR (500 MHz, CDCl_3_) δ 7.76 (d, J = 7.5 Hz, ^2^H), 7.58 (dd, J = 7.6, 4.0 Hz, ^2^H), 7.43–7.35 (m, ^3^H), 7.32–7.29 (m, ^3^H), 7.29–7.27 (m, ^3^H), 7.26–7.24 (m, ^1^H), 7.22 (d, *J* = 8.9 Hz, ^1^H), 5.94–5.76 (m, ^2^H), 5.29 (dd, *J* = 17.2, 1.6 Hz, ^1^H), 5.22 (d, *J* = 10.4 Hz, ^1^H), 4.73–4.66 (m, ^1^H), 4.63–4.56 (m, ^3^H), 4.54 (d, *J* = 12.1 Hz, ^1^H), 4.48 (d, *J* = 12.0 Hz, ^1^H), 4.42 (dd, *J* = 10.6, 7.3 Hz, ^1^H), 4.36 (dd, *J* = 10.6, 6.8 Hz, ^1^H), 4.31 (t, *J* = 5.6 Hz, ^2^H), 4.20 (t, *J* = 7.1 Hz, ^1^H), 3.90 (dd, *J* = 9.6, 4.2 Hz, ^1^H), 3.63 (dd, *J* = 9.6, 5.7 Hz, ^1^H), 3.59 (s, ^1^H), 1.38 (d, *J* = 7.2 Hz, ^3^H), 1.17 (d, *J* = 6.1 Hz, ^3^H). ^13^C-NMR (125 MHz, CDCl_3_) δ 172.7, 170.4, 169.3, 156.7, 143.9, 143.7, 141.4, 137.4, 131.6, 128.6, 128.1, 127.9, 127.9, 127.2 , 125.2, 125.2, 120.1, 118.9, 73.6, 69.2, 67.5, 67.4, 66.1, 59.0, 53.0, 48.5, 47.2, 18.3, 18.3. HRMS (ESI) calculated for C_35_H_39_N_3_O_8_Na+ [M + Na]^+^ 652.2629, found 652.2633.

#### 3.2.10. Allyl *O*-benzyl-*N*-*O*-benzyl-N-(tert-butoxycarbonyl)-d-seryl-l-threonyl-l-seryl-d-alaninate (**20**)

To a solution of 19 (1.5 g, 2.4 mmol, 1.0 eq.) in dry DCM (24 mL, 0.09 M) was added DBU (2.4 mL, 2.4 mmol, 1.0 eq., 1.0 M in DCM) at 0 °C under argon atmosphere. The reaction mixture was stirred for 1.5 h at 0 °C. After full conversion of the starting material as determined by TLC, HOAt (500 mg, 3.6 mmol, 1.5 eq.) and Boc-O-Bn-d-Ser (370 mg, 3.36 mmol, 2.0 eq.) were added at 0 °C, and the mixture was stirred for 5 min at the same temperature. Then, HATU (1.4 g, 3.6mmol, 1.5 eq.) was added at 0 °C and the resulting mixture was allowed to stir for 12 h at room temperature. The reaction mixture was quenched by the addition of H_2_O (30 mL). Layers were separated and the aqueous phase was extracted with EtOAc (3 × 50 mL). The combined organic layers were washed with brine (50 mL), dried over anhydrous Na_2_SO_4_, filtered and concentrated in vacuo. Purification of the crude product was performed by flash chromatography on silica gel (hexanes/EtOAc = 3/1-1/1) to afford 20 (1.3 g, 78%) as a white solid. TLC: R_f_ = 0.3 (hexanes/EtOAc = 1/1), UV & PMA stain. $\alpha_{D}^{26}=$ +2.3 (c 2.3, CHCl_3_). ^1^H-NMR (500 MHz, CDCl_3_) δ 7.47 (d, J = 7.1 Hz, ^1^H), 7.36 (d, J = 7.4 Hz, ^2^H), 7.33–7.28 (m, ^4^H), 7.28–7.23 (m, ^6^H), 5.87 (ddt, *J* = 16.5, 11.0, 5.7 Hz, ^1^H), 5.51 (d, *J* = 6.9 Hz, ^1^H), 5.39–5.26 (m, ^1^H), 5.22 (d, *J* = 10.4 Hz, ^1^H), 4.68–4.61 (m, ^1^H), 4.61–4.57 (m, ^2^H), 4.56–4.48 (m, ^4^H), 4.47–4.44 (m, ^2^H), 4.35–4.26 (m, ^2^H), 3.85 (dd, *J* = 9.3, 4.3 Hz, ^1^H), 3.84–3.79 (m, ^1^H), 3.65 (dd, *J* = 9.9, 5.6 Hz, ^1^H), 3.59 (dd, *J* = 9.4, 5.3 Hz, ^1^H), 1.42 (s, ^9^H), 1.37 (d, *J* = 7.2 Hz, ^3^H), 1.11 (d, *J* = 6.4 Hz, ^3^H). ^13^C- NMR (125 MHz, CDCl_3_) δ 172.6, 171.3, 170.2, 169.4, 155.6, 137.5, 137.4, 131.7, 128.6, 128.6, 128.0, 128.0, 127.9, 127.9, 118.7, 80.6, 73.5, 73.4, 69.7, 69.3, 66.9, 66.0, 57.9, 54.9, 53.2, 48.5, 28.4, 18.3, 18.1. HRMS (ESI) calculated for C_35_H_48_N_4_O_10_Na^+^ [M + Na]^+^ 707.3263, found 707.3260.

#### 3.2.11. (2.*R*,3*S*)-4-(((*S*)-1-(((*R*)-1-(Allyloxy)-1-oxopropan-2-yl)amino)-3-(benzyloxy)-1-oxopropan-2-yl)amino)-3-((*R*)-3-(benzyloxy)-2-((tert-butoxycarbonyl)amino)propanamido)-4-oxobutan-2-yl (((9H-fluoren-9-yl)methoxy)carbonyl)-l-phenylalaninate (**21**)

To a solution of **20** (1.64 g, 2.4 mmol, 1.0 eq.), Fmoc-l-Phe (1.86 g, 4.8 mmol, 2.0 eq.), Et_3_N (1.7 mL, 12.0 mmol, 5.0 eq.) and DMAP (29 mg, 0.24 mmol, 0.1 eq.) in anhydrous PhMe (15 mL, 0.12 M) was added MNBA (2.0 g, 6 mmol, 2.5 eq.) in anhydrous PhMe (5 mL) at 0 °C under an argon atmosphere. The reaction mixture was allowed to stir at room temperature for 3 h. The reaction mixture was quenched with saturated aqueous solution of NH_4_Cl (10 mL) and extracted with EtOAc (3 × 20 mL). The combined organic layers were washed with saturated aqueous solution of brine (20 mL), dried over anhydrous Na_2_SO_4_, filtered and concentrated in vacuo. Purification of the crude product was performed by flash chromatography on silica gel (hexanes/EtOAc = 2/1) to afford **21** (2.3 g, 90%) as a white solid. TLC: R*_f_* = 0.5 (hexanes/EtOAc = 1/1), UV & PMA stain. $\alpha_{D}^{26}$ = +6.1 (*c* 2.1, CHCl_3_). ^1^H-NMR (400 MHz, CDCl_3_) δ 7.75 (d, *J* = 7.6 Hz, ^2^H), 7.52 (dd, *J* = 10.2, 7.5 Hz, ^2^H), 7.38 (td, *J* = 7.5, 2.9 Hz, ^3^H), 7.33–7.27 (m, ^3^H), 7.28 (s, ^2^H), 7.30–7.24 (m, ^4^H), 7.28–7.22 (m, ^4^H), 7.26–7.19 (m, ^3^H), 7.20–7.13 (m, ^2^H), 7.03 (d, *J* = 7.5 Hz, ^1^H), 5.82 (ddt, *J* = 16.5, 10.9, 5.7 Hz, ^1^H), 5.69 (d, *J* = 7.3 Hz, ^1^H), 5.47 (d, *J* = 7.0 Hz, ^1^H), 5.44–5.33 (m, ^1^H), 5.30–5.21 (m, ^1^H), 5.19 (d, *J* = 10.4 Hz, ^1^H), 4.62–4.54 (m, ^6^H), 4.52 (s, ^1^H), 4.48 (s, ^1^H), 4.49–4.40 (m, ^2^H), 4.42–4.33 (m, ^1^H), 4.34 (d, *J* = 7.2 Hz, ^1^H), 4.28 (dd, *J* = 10.6, 6.9 Hz, ^1^H), 4.15 (t, *J* = 7.1 Hz, ^1^H), 3.86 (dd, *J* = 9.4, 4.3 Hz, ^1^H), 3.78 (dd, *J* = 9.6, 5.1 Hz, ^1^H), 3.63–3.53 (m, ^2^H), 3.09 (dd, *J* = 13.7, 6.8 Hz, ^1^H), 2.99 (dd, *J* = 13.7, 7.1 Hz, ^1^H), 1.44 (s, ^9^H), 1.32 (d, *J* = 7.2 Hz, ^3^H), 1.07 (d, *J* = 6.6 Hz, ^3^H). ^13^C-NMR (100 MHz, CDCl_3_) δ 172.3, 170.7, 170.6, 169.0, 168.2, 156.1, 155.7, 143.9, 143.8, 141.4, 137.4, 135.8, 131.6, 129.5, 128.7, 128.6, 128.5, 128.0, 128.0, 127.9, 127.8, 127.8, 127.3, 127.2, 127.2, 125.2, 125.2, 120.1, 118.8, 80.6, 73.5, 73.4, 70.1, 69.7, 69.1, 67.3, 66.0, 56.1, 55.5, 54.7, 53.1, 48.4, 47.1, 37.8, 28.4, 18.3, 15.5. HRMS (ESI) calculated for C_59_H_67_N_5_O_13_Na^+^ [M + Na]^+^ 1076.4628, found 1076.4622.

#### 3.2.12. ((Allyloxy)carbonyl)-d-phenylalanyl-l-valine (**22**)

Step A: To a solution of Alloc-d-Phe (7.5 g, 30.3 mmol, 1.0 eq.), l-valine methyl ester hydrochloride (10.2 g, 60.6 mmol, 2.0 eq.), HOAt (8.2 g, 60.6 mmol, 2.0 eq.) and DIPEA (20 mL, 121.2 mmol, 4.0 eq.) in dry DCM (200 mL, 0.15 M) under an argon atmosphere, was added EDCI (11.6 mg, 60.6 mmol, 2.0 eq.) at 0 °C. The mixture was allowed to stir for 12 h at room temperature and then concentrated in vacuo furnishing a solid residue. The solid residue was redissolved in EtOAc (5 mL) and quenched with 4% citric acid aqueous solution. The aqueous layer was extracted with EtOAc (3 × 10 mL). The combined organic layers were washed with saturated aqueous solution of NaHCO_3_ (100 mL), brine (100 mL), dried over anhydrous Na_2_SO_4_, filtered and concentrated in vacuo. Purification of the crude product was performed by flash chromatography on silica gel (hexanes/EtOAc = 3/1) to afford **S1** (9.9 g, 90%) as a white solid. TLC: R*_f_* = 0.5 (Hexanes/EtOAc = 2/1), UV & PMA stain. $\alpha_{D}^{27}$ = +8.7 (*c* 2.2, CHCl_3_). ^1^H-NMR (300 MHz, CDCl_3_) δ 7.33–7.21 (m, 2H), 7.25–7.14 (m, ^3^H), 6.61 (d, *J* = 8.7 Hz, ^1^H), 5.95–5.72 (m, ^1^H), 5.62 (d, *J* = 8.1 Hz, ^1^H), 5.31–5.15 (m, ^1^H), 5.21–5.10 (m, ^1^H), 4.61–4.53 (m, ^1^H), 4.51 (t, *J* = 1.4 Hz, ^1^H), 4.49 (t, *J* = 1.5 Hz, ^1^H), 4.45 (dd, *J* = 8.6, 5.0 Hz, ^1^H), 3.67 (s, ^3^H), 3.21–2.94 (m, ^2^H), 2.19–1.85 (m, ^1^H), 0.74 (t, *J* = 6.8 Hz, ^6^H). ^13^C-NMR (75 MHz, CDCl_3_) δ 172.1, 171.1, 155.9, 136.5, 132.6, 129.3, 128.7, 127.0, 117.8, 65.9, 57.3, 56.2, 52.2, 38.8, 31.1, 18.8, 17.7. HRMS (ESI) calculated for C_19_H_26_N_2_O_5_Na^+^ [M + Na]^+^ 385.1734, found 385.1730.
Step B: To a solution of S1 (5.0 g, 13.8 mmol, 1.0 eq.) in THF/H_2_O (20 mL/10 mL, 0.46 M) was added LiOH⋅H_2_O (1.7 g, 41.4 mmol, 3.0 eq.) at 0 °C. after being stirred for 2 h at room temperature, the organic solvents were evaporated. The reaction mixture was diluted with water (10 mL), acidified to pH = 2 with HCl (1.0 M in water), and extracted with EtOAc (3 × 20 mL). The combined organic layers were washed with saturated aqueous solution brine (20 mL), dried over anhydrous Na_2_SO_4_, filtered and concentrated in vacuo. Purification of the crude product was performed by flash chromatography on silica gel (Hexanes/EtOAc = 3/1) to afford **22** (4.4 g, 91%) as a white solid. TLC: R*_f_* = 0.6 (hexanes/EtOAc = 1/1), UV & PMA stain. $\alpha_{D}^{26}\;$= +9.6 (*c* 7.3, CHCl_3_). ^1^H-NMR (500 MHz, CDCl_3_) δ 9.08 (s, ^1^H), 7.34–7.23 (m, ^2^H), 7.24–7.15 (m, ^3^H), 7.08 (t, *J* = 11.0 Hz, ^1^H), 6.12 (t, *J* = 9.1 Hz, ^1^H), 5.82 (ddt, *J* = 16.4, 10.9, 5.3 Hz, ^1^H), 5.26–5.18 (m, ^1^H), 5.16 (d, *J* = 10.4 Hz, ^1^H), 4.80–4.70 (m, ^1^H), 4.50–4.46 (m, ^3^H), 3.14–2.89 (m, ^2^H), 2.13–1.94 (m, ^1^H), 0.79 (d, *J* = 6.8 Hz, ^3^H), 0.74 (d, *J* = 6.7 Hz, ^3^H). ^13^C-NMR (125 MHz, CDCl_3_) δ 174.1, 171.8, 156.4, 136.4, 132.4, 129.4, 128.7, 127.0, 117.9, 66.1, 57.4, 56.1, 39.3, 31.1, 18.6, 17.7. HRMS (ESI) calculated for C_18_H_24_N_2_O_5_Na^+^ [M + Na]^+^ 371.1577, found 371.1577.

#### 3.2.13. (2*R*,3*S*)-4- (((*S*)-1- (((*R*)-1- (allyloxy)-1-oxopropan-2-yl)amino)-3- (benzyloxy)-1-oxopropan-2-yl)amino)-3- ((*R*)-3-(benzyloxy)-2- ((*tert*-butoxycarbonyl)amino)propanamido)-4-oxobutan-2-yl ((allyloxy)carbonyl)-d-phenylalanyl-l-valyl-l-phenylalaninate (**5**)

To a solution of **21** (1.5 g, 1.4 mmol, 1.0 eq.) in dry DCM (15 mL, 0.09 M) was added DBU (1.4 mL, 1.4 mmol, 1.0 eq., 1.0 M in DCM) at 0 °C under an argon atmosphere. The reaction mixture was stirred for 1.5 h at 0 °C. After full conversion of the starting material as determined by TLC, HOAt (380 mg, 2.8 mmol, 2.0 eq.) and 22 (550 mg, 1.6 mmol, 1.1 eq.) were added at 0 °C, and the mixture was stirred for 5 min at the same temperature. Then, HATU (1.1 g, 2.8 mmol, 2.0 eq.) was added at 0 °C and the resulting mixture was allowed to stir for 12 h at room temperature. The reaction mixture was quenched by the addition of H_2_O (30 mL). Layers were separated and the aqueous phase was extracted with EtOAc (3 × 20 mL). The combined organic layers were washed with brine (20 mL), dried over anhydrous Na_2_SO_4_, filtered and concentrated in *vacuo*. Purification of the crude product was performed by flash chromatography on silica gel (hexanes/EtOAc = 2/3) to afford **5** (1.3 g, 80%) as a white solid. TLC: R*_f_* = 0.3 (hexanes/EtOAc = 2/3), UV & PMA stain.${\;\alpha }_{D}^{27}\;$= −2.6 (*c* 1.0, CHCl_3_). ^1^H-NMR (400 MHz, CDCl_3_) δ 7.65 (s, 1H), 7.59 (d, *J* = 7.1 Hz, ^1^H), 7.31 (t, *J* = 1.6 Hz, ^1^H), 7.29 (s, ^4^H), 7.28 (s, ^4^H), 7.25–7.24 (m, 1.7 Hz, ^2^H), 7.22 (d, *J* = 1.6 Hz, ^2^H), 7.20 (d, *J* = 2.2 Hz, ^3^H), 7.19 (s, ^2^H), 7.18–7.15 (m, ^4^H), 7.04 (d, *J* = 8.0 Hz, ^1^H), 6.73 (s, ^1^H), 6.49 (s, ^1^H), 5.92–5.86 (m, ^1^H), 5.85–5.79 (m, ^1^H), 5.60 (d, *J* = 7.5 Hz, ^1^H), 5.35–5.25 (m, 1H), 5.28–5.20 (m, 1H), 5.24–5.18 (m, 1H), 5.19–5.10 (m, 1H), 4.82 (t, *J* = 6.3 Hz, 1H), 4.77–4.69 (m, ^1^H), 4.65–4.60 (m, ^3^H), 4.59–4.57 (m, ^2^H), 4.57–4.56 (m, ^01^H), 4.53 (s, ^3^H), 4.48 (d, *J* = 11.9 Hz, ^2^H), 4.41–4.30 (m, ^1^H), 4.09–3.99 (m, ^1^H), 3.95–3.88 (m, ^1^H), 3.83 (dd, *J* = 9.6, 4.7 Hz, ^1^H), 3.74–3.56 (m, ^1^H), 3.21 (dd, *J* = 14.1, 5.6 Hz, ^1^H), 3.06 (dd, *J* = 13.7, 8.0 Hz, ^1^H), 2.98 (dd, *J* = 14.1, 8.9 Hz, 2H), 1.95–1.85 (m, 1H), 1.43 (s, 9H), 1.38 (d, *J* = 7.2 Hz, 3H), 1.12 (d, *J* = 6.4 Hz, ^3^H), 0.60–0.51 (m, ^6^H). ^13^C-NMR (100 MHz, CDCl_3_) δ 172.6, 172.5, 171.2, 171.0, 170.2, 169.3, 168.6, 157.0, 155.8, 137.7, 137.6, 136.9, 136.6, 132.7, 131.7, 129.3, 129.3, 128.7, 128.5, 127.9, 127.8, 127.0, 126.9, 118.7, 117.8, 80.4, 73.4, 73.3, 70.7, 70.1, 69.3, 66.3, 66.0, 59.4, 57.2, 55.7, 54.4, 53.9, 48.5, 38.0, 36.7, 29.7, 28.4, 19.1, 18.2, 17.6, 15.6. HRMS (ESI) calculated for C_62_H_79_N_7_O_15_Na^+^ [M + Na]^+^ 1184.5526, found 1184.5530.

#### 3.2.14. Ethyl (*S*)-2-((2-(1-(dimethylamino)-2-methylpropyl)thiazole-4-carboxamido)methyl)thiazole-4-carboxylate (**3**)

Step A: A solution of **24** [22] (5.0 g, 18.9 mmol, 1.0 eq.) and formaldehyde solution (37% in H_2_O, 10.3 mL, 375 mmol) in formic acid (10 mL, 1.89 M) was stirred for 12 h at 80 °C. After cooling to room temperature, concentrated HCl (3 mL) was slowly added to the reaction mixture. All volatiles were removed in vacuo, and the resulting residue was extracted with Et_2_O (3 × 20 mL). The aqueous layer was added saturated aqueous solution of NaHCO_3_ (20 mL) and extracted with Et_2_O (3 × 20 mL). The combined organic layers were washed with brine (20 mL), dried over anhydrous Na_2_SO_4_, filtered and concentrated in vacuo providing **S2** (3.15 g, 65%) as a colorless oil. TLC: R*_f_* = 0.5 (hexanes/EtOAc = 1/1), UV & PMA stain. $\alpha_{D}^{22}\;$= +1.6 (*c* 1.0, MeOH). ^1^H-NMR (300 MHz, CDCl_3_) δ 8.03 (s, ^1^H), 5.19 (s, ^1^H), 4.28 (q, *J* = 7.1 Hz, ^2^H), 3.51 (d, *J* = 8.9 Hz, ^1^H), 2.11 (s, ^6^H), 1.26 (t, *J* = 7.1 Hz, ^3^H), 0.89 (d, *J* = 6.6 Hz, ^3^H), 0.69 (d, *J* = 6.6 Hz, ^3^H). ^13^C NMR (75 MHz, CDCl_3_) δ 169.6, 161.3, 146.2, 127.1, 72.9, 61.1, 41.6, 30.1, 20.0, 18.9, 14.2. HRMS (ESI) calculated for C_12_H_20_N_2_O_2_SNa^+^ [M + Na]^+^ 279.1138, found 279.1140.
Step B: To a solution of S2 (3.15 g, 12.3 mmol, 1.0 eq.) in THF/H_2_O (10 mL/5 mL, 0.8 M) was added LiOH⋅H_2_O (1.55 g, 36.9 mmol, 3.0 eq.) at 0 °C. After being stirred for 2 h at room temperature, the reaction mixture was quenched with concentrated HCl (5 mL) and all solvents were removed in *vacuo* providing crude product **S3** which was used directly in the next step without further purification.
  Step C: To a solution of the crude **S3** in DCM (20 mL, 0.6 M) at 0 °C was added DIPEA (10.1 mL, 61.5 mmol, 5.0 eq.), followed by consecutive addition of **25** [23] (4.6 g, 24.6 mmol, 2.0 eq.), HOAt (3.4 g, 24.6 mmol, 2.0 eq.) and HATU (9.4 g, 24.6 mmol, 2.0 eq.). The mixture was allowed to stir for 12 h at room temperature and then concentrated in *vacuo* furnishing a solid residue. Purification of the crude product was performed by flash chromatography on silica gel (MeOH/DCM = 1/40) to afford **3** (3.8 g, 84% for 2 steps) as a colorless oil. TLC: R*_f_* = 0.4 (MeOH/DCM = 1/20), UV & PMA stain. $\alpha_{D}^{27}\;$= +0.5 (*c* 0.75, CHCl_3_). ^1^H-NMR (400 MHz, CDCl_3_) δ 8.10 (s, ^1^H), 8.07 (s, ^1^H), 4.93 (d, *J* = 6.5 Hz, ^2^H), 4.37 (q, *J* = 7.1 Hz, ^2^H), 3.38 (d, *J* = 9.2 Hz, ^1^H), 2.75 (s, ^2^H), 2.17 (s, ^6^H), 1.35 (t, *J* = 7.1 Hz, ^3^H), 0.98 (d, *J* = 6.7 Hz, ^3^H), 0.76 (d, *J* = 6.6 Hz, ^3^H). ^13^C-NMR (100 MHz, CDCl_3_) δ 169.3, 168.5, 161.5, 161.3, 148.4, 146.8, 128.4, 123.7, 73.0, 61.6, 41.8, 40.8, 38.6, 30.1, 20.2, 19.3, 14.4. HRMS (ESI) calculated for C_17_H_24_N_4_O_3_S_2_Na^+^ [M + Na]^+^ 419.1182, found 419.1187.

#### 3.2.15. *tert*-Butyl ((*R*)-3-(benzyloxy)-1- (3*S*,6*S*,9*R*,12*R*,15*S*,18*S*,19*R*)-3,9-dibenzyl-15-((benzyloxy)methyl)-6-isopropyl-12,19-dimethyl-2,5,8,11,14,17-hexaoxo-1-oxa-4,7,10,13,16-pentaazacyclononadecan-18-yl)amino)-1-oxopropan-2-yl)carbamate (**2**))

To a stirred solution of **5** (750 mg, 0.65 mmol, 1.0 eq.) in THF (10 mL, 0.04 M) were added PMHS (1.5 mL, 6.5 mmol, 10.0 eq.), Pd(PPh_3_)_4_ (75 mg, 0.065 mmol, 0.1 eq.) and ZnCl_2_ (6.5 mL, 6.5 mmol, 10.0 eq., 1 M in THF) at room temperature. The reaction mixture was stirred for 3 h at the same temperature. The mixture was diluted with saturated aqueous solution of NaHCO_3_ (10 mL), brine (10 mL), extracted with EtOAc (3 × 50 mL), dried over anhydrous Na_2_SO_4_, filtered and concentrated in vacuo. Purification of the crude product was performed by flash chromatography on silica gel (MeOH/DCM = 1/40-1/20) to afford the crude amino acid as a white solid. To a solution of the above crude amino acid in dry DCM (700 mL, 0.001 M) were added HOAt (177 mg, 1.3 mmol, 2.0 eq.) and DIPEA (0.5 mL, 2.6 mmol, 4.0 eq.) and HATU (494 mg, 1.3 mmol, 2.0 eq.) under argon atmosphere at 0 °C. The mixture was allowed to stir for 12 h at room temperature and then concentrated in vacuo furnishing a solid residue. The solid residue was redissolved in EtOAc (20 mL) and quenched with 4% citric acid aqueous solution. The aqueous layer was extracted with EtOAc (3 × 20 mL). The combined organic layers were washed with saturated aqueous solution of NaHCO_3_ (20 mL), brine (20 mL), dried over anhydrous Na_2_SO_4_, filtered and concentrated in vacuo. Purification of the crude product was performed by flash chromatography on silica gel (hexanes/EtOAc = 2/1) to afford **2** (397 mg, 60% for 2 steps) as a white solid. TLC: R_f_ = 0.5 (hexanes/EtOAc = 1/1), UV & PMA stain. $\alpha_{D}^{27}\;$= +17.9 (c 7.9, CHCl_3_). ^1^H-NMR (500 MHz, CDCl_3_) δ 7.82 (d, *J* = 8.8 Hz, ^1^H), 7.67 (d, *J* = 9.6 Hz, ^1^H), 7.34 (d, *J* = 7.5 Hz, ^1^H), 7.31 (dd, *J* = 8.0, 6.2 Hz, ^4^H), 7.28–7.26 (m, ^2^H), 7.25 (s, ^3^H), 7.24–7.22 (m, ^2^H), 7.22–7.21 (m, ^2^H), 7.20–7.18 (m, ^3^H), 7.17–7.11 (m, ^5^H), 7.03–6.95 (m, ^1^H), 6.85 (dd, *J* = 10.1, 2.7 Hz, ^1^H), 6.79–6.72 (m, ^1^H), 6.02 (d, *J* = 8.1 Hz, ^1^H), 5.64–5.53 (m, ^1^H), 5.25 (ddd, *J* = 11.6, 9.9, 4.5 Hz, ^1^H), 5.02 (dd, *J* = 9.6, 2.7 Hz, ^1^H), 4.92–4.82 (m, ^1^H), 4.71–4.62 (m, ^1^H), 4.59 (d, *J* = 12.0 Hz, ^1^H), 4.55–4.48 (m, ^2^H), 4.46 (d, *J* = 12.1 Hz, ^1^H), 4.22 (dd, *J* = 7.9, 3.6 Hz, ^1^H), 4.17 (d, *J* = 7.6 Hz, ^1^H), 4.11 (qd, *J* = 7.2, 4.6 Hz, ^1^H), 3.90 (dd, *J* = 10.0, 5.6 Hz, ^1^H), 3.74 (dd, *J* = 10.0, 4.7 Hz, ^1^H), 3.64 (dd, *J* = 9.4, 6.3 Hz, ^1^H), 3.60 (dd, *J* = 9.5, 7.7 Hz, ^1^H), 3.38 (dd, *J* = 14.1, 4.5 Hz, ^1^H), 3.31–3.25 (m, ^2^H), 3.00 (dd, *J* = 14.2, 11.8 Hz, ^1^H), 2.20–2.09 (m, ^1^H), 1.35 (s, ^9^H), 1.18 (d, *J* = 7.3 Hz, ^3^H), 1.12 (d, *J* = 6.2 Hz, ^3^H), 0.77 (d, *J* = 7.0 Hz, ^3^H), 0.30 (d, *J* = 6.9 Hz, ^3^H). ^13^C-NMR (125 MHz, CDCl_3_) δ 173.7, 173.3, 172.0, 171.8, 170.8, 170.5, 169.7, 155.5, 137.8, 137.6, 136.9, 129.6, 129.4, 129.1, 128.7, 128.5, 128.4, 128.3, 127.9, 127.9, 127.7, 126.4, 126.1, 125.8, 79.7, 73.7, 73.1, 70.8, 70.3, 68.0, 59.4, 55.8, 55.2, 54.8, 52.6, 52.3, 51.4, 36.5, 34.1, 29.4, 28.2, 19.7, 16.9, 16.6, 16.0. HRMS (ESI) calculated for C_55_H_70_N_7_O_12_^+^ [M + H]^+^ 1020.5077, found 1020.5078.

#### 3.2.16. Pagoamide A (**1**)

Step A: To a solution of **2** (180 mg, 0.18 mmol, 1.0 eq.) in DCM (3 mL, 0.05 M) was added TFA (1 mL) dropwise at 0 °C. After being stirred at room temperature for 3 h, the reaction mixture was concentrated in vacuo to afford the crude amine **3**, which was used directly in the next step without further purification. To a solution of **3** (285.0 mg, 0.72 mmol, 4.0 eq.) in THF/H_2_O (4 mL/2 mL, 0.12 M) was added LiOH⋅H_2_O (92.0 mg, 2.2 mmol, 12.0 eq.) at 0 °C. After being stirred for 3 h at room temperature, the reaction mixture was quenched with concentrated HCl (1 mL), and all solvent was removed in vacuo providing the crude acid which was used directly in the next step without further purification. To a solution of the above crude amine (0.18 mmol, 1.0 eq.) and the crude acid (0.72 mmol, 4.0 eq.) in DMF (5 mL, 0.09 M) were added HOAt (98 mg, 0.72 mmol, 4.0 eq.), Et_3_N (0.2 mL, 1.4 mmol, 8.0 eq.), DMAP (11.0 mg, 0.04 mmol, 0.5 eq.) and EDCI (138.0 mg, 0.72 mmol, 4.0 eq.) under argon atmosphere at 0 °C. The mixture was allowed to stir for 36 h at room temperature and then concentrated in vacuo furnishing a solid residue. The solid residue was redissolved in EtOAc (10 mL) and quenched with saturated aqueous solution of NaHCO_3_ (10 mL). The aqueous layer was extracted with EtOAc (3 × 10 mL). The combined organic layers were washed with brine (10 mL), dried over anhydrous Na_2_SO_4_, filtered and concentrated in vacuo. Purification of the crude product was performed by flash chromatography on silica gel (MeOH/DCM = 1/40-1/20) to afford **26** (194 mg, 85%) as a white solid. TLC: R*_f_* = 0.4 (MeOH/DCM = 1/10), UV & PMA stain. $\alpha_{D}^{31}$ = +9.7 (*c* 1.0, CHCl_3_). ^1^H-NMR (500 MHz, CDCl_3_) δ 8.07 (dd, *J* = 7.6, 2.2 Hz, ^1^H), 8.05–8.02 (m, ^2^H), 7.98 (d, *J* = 9.4 Hz, ^1^H), 7.94 (d, *J* = 8.8 Hz, ^1^H), 7.92 (s, ^1^H), 7.33–7.27 (m, ^6^H), 7.27–7.23 (m, ^5^H), 7.23–7.19 (m, ^3^H), 7.18–7.13 (m, ^5^H), 7.10 (t, *J* = 7.5 Hz, ^2^H), 7.01 (t, *J* = 7.3 Hz, ^1^H), 6.89 (s, ^1^H), 6.85 (d, *J* = 10.0 Hz, ^1^H), 5.66 (qd, *J* = 6.3, 2.7 Hz, ^1^H), 5.41–5.33 (m, ^1^H), 5.25 (ddd, *J* = 11.7, 9.9, 4.5 Hz, ^1^H), 5.11 (dd, *J* = 9.6, 2.7 Hz, ^1^H), 4.82 (dd, *J* = 16.1, 6.4 Hz, ^1^H), 4.74 (ddd, *J* = 16.1, 8.0, 6.3 Hz, ^1^H), 4.69–4.58 (m, ^2^H), 4.52 (d, *J* = 12.4 Hz, ^2^H), 4.43 (d, *J* = 12.0 Hz, ^1^H), 4.16 (dd, *J* = 6.7, 3.8 Hz, ^1^H), 4.12 (dd, *J* = 7.3, 5.1 Hz, ^1^H), 4.09 (dd, *J* = 7.3, 3.9 Hz, ^1^H), 4.06 (ddd, *J* = 9.9, 4.3, 1.8 Hz, ^1^H), 3.86 (dd, *J* = 10.0, 5.0 Hz, ^1^H), 3.61 (d, *J* = 7.0 Hz, ^12^H), 3.46 (d, *J* = 9.0 Hz, 1H), 3.41–3.32 (m, 2H), 3.28–3.21 (m, 1H), 3.03 (dd, *J* = 14.2, 11.7 Hz, 1H), 2.29–2.21 (m, ^1^H), 2.22 (s, ^6^H), 2.13–2.02 (m, ^1^H), 1.20 (d, *J* = 7.3 Hz, ^3^H), 1.15 (d, *J* = 6.2 Hz, ^3^H), 1.02 (d, *J* = 6.6 Hz, ^3^H), 0.82 (d, *J* = 6.6 Hz, ^3^H), 0.75 (d, *J* = 6.9 Hz, ^3^H), 0.32 (d, *J* = 6.9 Hz, ^3^H). ^13^C-NMR (125 MHz, CDCl_3_) δ 173.9, 173.5, 172.0, 171.3, 171.1, 170.8, 169.8, 168.1, 161.7, 160.7, 149.5, 148.4, 137.9, 137.8, 137.8, 137.1, 129.5, 129.2, 128.7, 128.5, 128.5, 128.2, 128.0, 127.8, 127.7, 126.5, 126.2, 124.3, 124.0, 73.5, 73.2, 73.2, 70.6, 70.4, 68.1, 59.8, 55.8, 55.5, 53.3, 52.6, 52.4, 51.4, 41.8, 41.0, 36.5, 33.8, 30.2, 29.8, 29.6, 20.3, 19.7, 19.4, 16.9, 16.7, 16.3. HRMS (ESI) calculated for C_65_H_80_N_11_O_12_S_2_^+^ [M + Na]^+^ 1270.5424, found 1270.5429.
Step B: To a solution of 26 (32 mg, 0.025 mmol, 1.0 eq.) in anhydrous DCM (3 mL, 0.008 M) was added BCl_3_ (0.25 mL, 0.25 mmol, 10.0 eq.,1 M in DCM) at −78 °C under argon atmosphere. The reaction mixture was stirred at the same temperature for 4 h. Then, MeOH was added while the temperature was still at −78 °C, and the mixture was allowed to reach room temperature. The solvent was removed in *vacuo* furnishing a solid residue. The solid residue redissolved in DCM (5 mL) and saturated aqueous solution of NaHCO_3_ (10 mL) was added. The aqueous layer was extracted with DCM (3 × 10 mL). The combined organic layers were washed with brine (10 mL), dried over anhydrous Na_2_SO_4_, filtered and concentrated in vacuo. Purification of the crude product was performed by flash chromatography on silica gel (MeOH/DCM = 1/40-1/20) to afford pagoamide A (**1**) (16.3 mg, 60%) as a white solid. TLC: R*_f_* = 0.2 (MeOH/DCM = 1/10), UV & PMA stain. $\alpha_{D}^{26}\;$= +8.0 (*c* 0.1, MeOH). ^1^H-NMR (500 MHz, DMSO-*d*_6_) δ 9.24 (t, *J* = 6.3 Hz, ^1^H), 9.14–8.99 (m, ^1^H), 8.36 (d, *J* = 5.7 Hz, ^1^H), 8.31 (s, ^1^H), 8.20 (d, *J* = 9.0 Hz, ^1^H), 8.09 (s, ^1^H), 8.06 (d, *J* = 8.0 Hz, ^1^H), 7.74 (d, *J* = 9.2 Hz, ^1^H), 7.38 (d, *J* = 7.6 Hz, ^1^H), 7.22 (d, *J* = 4.4 Hz, ^4^H), 7.20 (d, *J* = 7.2 Hz, ^2^H), 7.19–7.13 (m, ^3^H), 7.16–7.10 (m, ^1^H), 6.85 (d, *J* = 9.5 Hz, ^1^H), 5.70–5.61 (m, ^1^H), 4.91–4.85 (m, ^1^H), 4.85 (dt, *J* = 10.2, 2.8 Hz, ^2^H), 4.74 (d, *J* = 6.0 Hz, ^2^H), 4.60 (td, *J* = 9.8, 4.9 Hz, ^1^H), 4.09–4.00 (m, ^1^H), 3.94 (dt, *J* = 10.4, 4.8 Hz, ^1^H), 3.87 (dd, *J* = 7.8, 4.5 Hz, ^1^H), 3.75 (dt, *J* = 10.2, 4.6 Hz, ^1^H), 3.64–3.54 (m, ^3^H), 3.27–3.17 (m, ^2^H), 3.17–3.10 (m, ^2^H), 2.31–2.22 (m, ^1^H), 2.17 (s, ^6^H), 1.93–1.81 (m, ^1^H), 1.15 (d, *J* = 7.3 Hz, ^3^H), 1.12 (d, *J* = 6.2 Hz, ^3^H), 1.00 (d, *J* = 6.6 Hz, ^3^H), 0.75 (d, *J* = 6.6 Hz, ^3^H), 0.59 (d, *J* = 6.9 Hz, ^3^H), 0.28 (d, *J* = 6.9 Hz, ^3^H). ^13^C-NMR (125 MHz, DMSO-*d*_6_) δ 173.5, 172.5, 171.3, 170.4, 170.2, 170.0, 169.9, 169.1, 168.4, 161.2, 159.7, 148.8, 148.5, 138.0, 137.8, 129.2, 129.1, 128.1, 128.0, 126.2, 126.1, 124.4, 124.3, 71.9, 71.3, 62.3, 60.7, 58.7, 57.3, 54.6, 54.1, 52.9, 52.4, 50.1, 41.3, 40.8, 36.1, 34.2, 29.5, 29.3, 20.1, 19.4, 19.0, 16.6, 16.4, 15.9. HRMS (ESI) calculated for C_51_H_67_N_11_O_12_S_2_Na^+^ [M + Na]^+^ 1112.4304, found 1112.4300.

## 4. Conclusions

In summary, the first total synthesis of the depsipeptide pagoamide A (**1**), was accomplished efficiently. Obstacles were circumvented by judicious synthetic design that featured a sequence-dependent esterification via Shiina conditions. The route entails a longest sequence of nine linear steps to access pagoamide A (**1**) starting from **17**, which served to support the unequivocal structural elucidation of this natural product.

## Figures and Tables

**Table 1:** Optimization of macrolactonization.

Entry	Conditions	Yield
1	MNBA, DMAP, DCM, r.t.	20%
2	MNBA, DMAP, DCM/THF (1:1), r.t.	10%
3	MNBA, DMAP, CH3CN, r.t.	5%
4	TCBC, Et3N, DCM; then DMAP, PhMe, r.t.	5%
5	TCBC, Et3N, PhMe; then DMAP, PhMe, r.t.	trace
6	BEP, DMAP, DCM/DMF (10:1), r.t. to 40 °C	5%
7	EDCI, DMAP, DCM/DMF (10:1), 60 °C	5%
8	DCC, DMAP, DMAP⋅HCl, CHCl3, 65 °C	5%
9	TCFH, NMI, CH3CN, r.t. to 40 °C	trace
10	TCFH, NMI, DMF, r.t. to 40 °C	trace

**Table 2:** Comparison of the ^13^C-NMR data of **1** with literature data [1].

No.	Natural (δ_1_)	Sample 1 (δ_2_)	δ = δ_1_−δ_2_	No.	Natural (δ_1_)	Sample 1 (δ_2_)	δ = δ_1_−δ_2_
1	170.4	170.3	0.1	29	60.7	60.7	0
2	52.4	52.4	0	30	169.1	169	0.1
3	36.1	36.1	0	31	54.1	54.1	0
4	137.9	137.8	0.1	32	71.3	71.4	−0.1
5,9	128.1	128.1	0	33	16	15.9	0.1
6,8	129.2	129.2	0	34	169.9	169.9	0
7	126.2	126.2	0	35	54.5	54.6	−0.1
10	170	170	0	36	62.4	62.3	0.1
11	58.7	58.7	0	37	159.7	159.8	−0.1
12	29.3	29.3	0	38	148.8	148.8	0
13	19.1	19	0.1	39	124.2	124.3	−0.1
14	16.4	16.3	0.1	40	170.3	170.2	0.1
15	172.5	172.5	0	41	40.8	40.8	0
16	53	53	0	42	161.1	161.2	−0.1
17	34.1	34.1	0	43	148.5	148.4	0.1
18	138.1	138	0.1	44	124.4	124.4	0
19,23	128	128	0	45	168.4	168.4	0
20,22	129.1	129.1	0	46	71.9	71.9	0
21	126.1	126.1	0	47	29.5	29.5	0
24	173.5	173.5	0	48	19.4	19.3	0.1
25	50.1	50.1	0	49	20.1	20.1	0
26	16.7	16.6	0.1	50	41.3	41.3	0
27	171.4	171.3	0.1	51	41.3	41.3	0
28	57.4	57.5	−0.1				

## Data Availability

Not applicable.

## Supplementary Materials

The following are available online. detailed procedures for the synthesis of 1a and ^1^H- and ^13^C-NMR charts of all the compounds.

## Sample Availability

Not available.
