# Supplementary material for: Total Synthesis of Pagoamide A
Source: Molecules. 2021 Jul 12;26(14):4224. doi: 10.3390/molecules26144224 (PMC8307129; doi:10.3390/molecules26144224)

Supporting Information

Total Synthesis of Pagoamide A

Fusong Wu[a,#], Jie Yu[a,#], Jiawei Meng,[a] Yian Guo*,[a,b] and Tao Ye*,[a]

Table of Contents

1. The esterification between alcohol **20** and acid **14** or **15** 2
2. Synthesis of pagoamide A diastereomer **1a** 2
3. Comparison of NMR spectra of natural and synthetic pagoamide A 8
4. NMR spectra 9

1. The esterification between alcohol 20 and acid 14 or 15

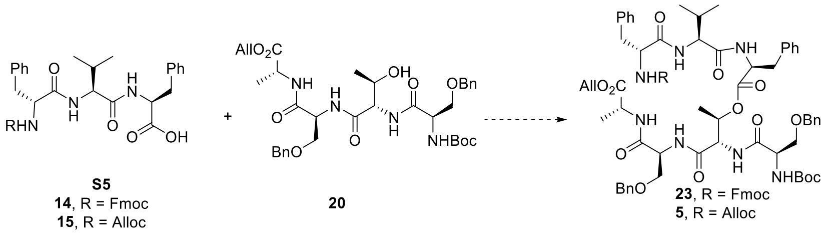

Table 1.The coupling between alcohol **20** and acid **14** or **15**

entry	S5	condition	yield
1	**14**	MNBA, DIPEA, DMAP, 4Å MS, toluene/THF	trace
2	**14**	TCBC, Et_3N, toluene; then DMAP	trace
3	**14**	DCC, DMAP, HOAt, DCM	trace
4	**14**	BEP, DMAP, DCM	trace
5	**14**	TCBC, Et_3N, DCM; then DMAP	trace
6	**14**	MNBA, DIPEA, DMAP, 4Å MS, DCM	trace
7	**15**	MNBA, DIPEA, DMAP, toluene	trace
8	**15**	TCBC, Et_3N, toluene; then DMAP	trace
9	**15**	DCC, DMAP, DMAP·HCl, DCM	trace
10	**15**	EDCI, Et_3N,DMAP, HOAt, DCM	trace
11	**15**	MNBA, DMAP, 4Å MS, toluene/THF	trace
12	**15**	PyBOP, DIPEA, DCM	trace
13	**15**	$SOCl_2$, then Et_3N, DMAP	trace

2. Synthesis of pagoamide A diastereomer 1a

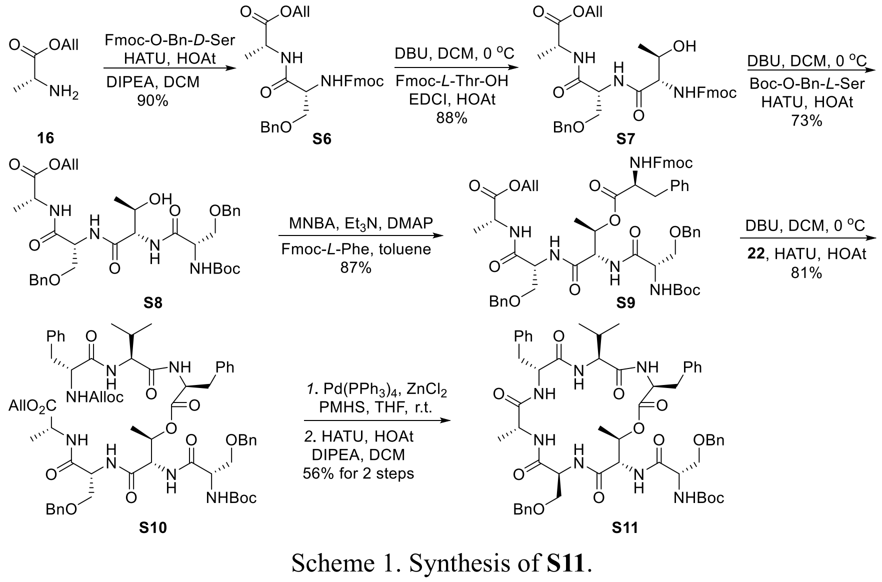

Scheme 1. Synthesis of **S11**.

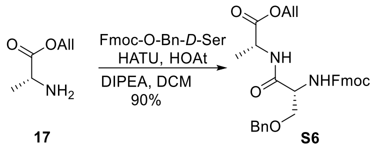

S6 was synthesized according to the procedures for the synthesis of **18** from **16** (5.1 g, 39.6 mmol, 2.0 eq.) and Fmoc-O-Bn-D-Ser (8.0 g, 19.8 mmol, 1.0 eq.). Purification of the crude product was performed by flash chromatography on silica gel (Hexanes/EtOAc = 4/1) to afford **S6** (9.4 g, 90%) as a white solid.
TLC: R_f = 0.5 (Hexanes/EtOAc = 3/1), UV & PMA stain.
$[\alpha]_D^{24}$ = -9.8 (*c* 1.7, $CHCl_3$).
^{1}H NMR (400 MHz, $CDCl_3$) δ 7.77 (d, *J* = 7.5 Hz, 2H), 7.61 (dd, *J* = 7.7, 3.1 Hz, 2H), 7.41 (t, *J* = 7.5 Hz, 2H), 7.38 – 7.32 (m, 5H), 7.32 – 7.28 (m, 2H), 7.24 (s, 1H), 5.98 – 5.83 (m, 2H), 5.33 (d, *J* = 17.2 Hz, 1H), 5.26 (d, *J* = 10.5 Hz, 1H), 4.71 – 4.60 (m, 3H), 4.59 (s, 2H), 4.50 – 4.38 (m, 3H), 4.23 (t, *J* = 7.1 Hz, 1H), 3.91 (dd, *J* = 9.4, 4.4 Hz, 1H), 3.62 (t, *J* = 8.5 Hz, 1H), 1.43 (d, *J* = 7.2 Hz, 3H).
^{13}C NMR (100 MHz, $CDCl_3$) δ 172.1, 169.6, 156.1, 143.8, 143.7, 141.3, 137.4, 131.5, 128.5, 127.9, 127.8, 127.7, 127.1, 125.1, 120.0, 118.7, 73.5, 69.9, 67.2, 65.9, 54.0, 48.4, 47.1, 18.2.
HRMS (ESI) calculated for $C_{31}H_{32}N_2O_6Na^+$ $[M+Na]^+$ 551.2153, found 551.2156.

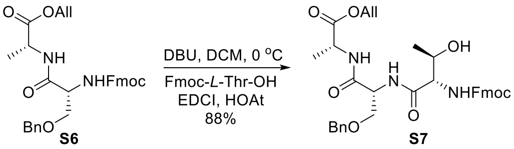

S7 was synthesized according to the procedures for the synthesis of **19** from **S6** (3.6 g, 7.0 mmol, 1.0 eq.) and Fmoc-L-Thr (2.4 g, 13.9 mmol, 1.0 eq.). Purification of the crude product was performed by flash chromatography on silica gel (MeOH/DCM = 1/60) to afford **S7** (3.2 g, 88%) as a white solid.
TLC: R_f = 0.7 (MeOH/DCM = 1/20), UV & PMA stain.
$[\alpha]_D^{27}$ = -0.1 (*c* 1.0, DMSO).
^{1}H NMR (500 MHz, $CDCl_3$) δ 7.76 (d, *J* = 7.5 Hz, 2H), 7.59 (t, *J* = 6.3 Hz, 2H), 7.47 – 7.36 (m, 2H), 7.32 – 7.29 (m, 4H), 7.28 – 7.25 (m, 3H), 7.18 (d, *J* = 7.7 Hz, 1H), 7.10 (d, *J* = 7.7 Hz, 1H), 5.87 (ddt, *J* = 16.5, 11.0, 5.8 Hz, 1H), 5.78 (d, *J* = 7.9 Hz, 1H), 5.30 (d, *J* = 17.2 Hz, 1H), 5.24 (d, *J* = 10.4 Hz, 1H), 4.66 – 4.59 (m, 1H), 4.62 – 4.55 (m, 3H), 4.56 – 4.49 (m, 3H), 4.44 (d, *J* = 6.5 Hz, 1H), 4.38 (dd, *J* = 10.6, 6.8 Hz, 1H), 4.22 (t, *J* = 7.0 Hz, 1H), 4.14 (d, *J* = 7.9 Hz, 1H), 3.95 (dd, *J* = 9.4, 3.5 Hz, 1H), 3.61 (dd, *J* = 9.2, 5.9 Hz, 1H), 3.55 (s, 1H), 1.38 (d, *J* = 7.2 Hz, 3H), 1.18 (d, *J* = 6.4 Hz, 3H).
^{13}C NMR (125 MHz, $CDCl_3$) δ 172.9, 171.6, 169.3, 157.0, 143.9, 143.7, 141.5, 137.3, 131.5, 128.6, 128.1, 127.9, 127.9, 127.2, 125.2, 125.1, 120.2, 119.0, 73.6, 69.1, 67.5, 67.3, 66.3, 60.0, 53.2, 48.5, 47.3, 19.1, 18.6.
HRMS (ESI) calculated for $C_{35}H_{39}N_3O_8Na+$ $[M+Na]^+$ 652.2629, found 652.2625.

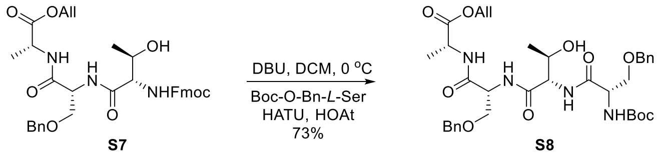

S8 was synthesized according to the procedures for the synthesis of **20** from **S7** (2.4 g, 3.8 mmol, 1.0 eq.) and Boc-O-Bn-L-Ser (592 mg, 5.4 mmol, 2.0 eq.). Purification of the crude product was performed by flash chromatography on silica gel (Hexanes/EtOAc = 20/1) to afford **S8** (1.9 g, 73%) as a white solid.
TLC: R_f = 0.3 (Hexanes/EtOAc = 1/1), UV & PMA stain.
$[\alpha]_D^{27}$ = -9.4 (*c* 2.7, $CHCl_3$).

^{1}H NMR (500 MHz, $CDCl_3$) δ 7.37 (d, *J* = 7.6 Hz, 1H), 7.37 – 7.31 (m, 2H), 7.34 – 7.29 (m, 4H), 7.32 – 7.26 (m, 4H), 7.29 – 7.25 (m, 1H), 7.20 (d, *J* = 7.7 Hz, 1H), 5.87 (ddt, *J* = 17.3, 10.4, 5.7 Hz, 1H), 5.47 (d, *J* = 5.5 Hz, 1H), 5.35 – 5.26 (m, 1H), 5.28 – 5.21 (m, 1H), 4.67 – 4.59 (m, 1H), 4.63 – 4.56 (m, 2H), 4.59 – 4.52 (m, 3H), 4.55 – 4.46 (m, 2H), 4.49 – 4.42 (m, 1H), 4.31 – 4.21 (m, 2H), 3.86 (dt, *J* = 9.5, 4.7 Hz, 2H), 3.75 – 3.61 (m, 2H), 2.01 (s, 1H), 1.40 (s, 9H), 1.34 (d, *J* = 7.2 Hz, 3H), 1.17 (d, *J* = 6.4 Hz, 3H).

^{13}C NMR (125 MHz, $CDCl_3$) δ 172.8, 171.8, 171.1, 169.70 156.2, 137.4, 137.3, 131.6, 128.6, 128.6, 128.1, 128.0, 128.0, 127.9, 118.9, 80.9, 73.6, 69.5, 69.1, 67.1, 66.1, 58.9, 55.2, 53.2, 48.4, 48.4, 28.4, 19.3, 18.4.

HRMS (ESI) calculated for $C_{35}H_{48}N_4O_{10}Na^+$ $[M+Na]^+$ 707.3263, found 707.3261.

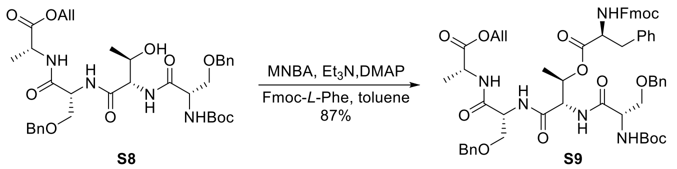

S9 was synthesized according to the procedures for the synthesis of **21** from **S8** (2.5 g, 3.6 mmol, 1.0 eq.) and Fmoc-L-Phe (2.8 g, 7.2 mmol, 2.0 eq.). Purification of the crude product was performed by flash chromatography on silica gel (Hexanes/EtOAc = 2/1) to afford **S9** (3.3 g, 87%) as a white solid.

TLC: R_f = 0.5 (Hexanes/EtOAc = 1/1), UV & PMA stain.

$[\alpha]_D^{26}$ = -0.6 (*c* 3.2, $CHCl_3$).

^{1}H NMR (300 MHz, $CDCl_3$) δ 7.75 (d, *J* = 7.5 Hz, 2H), 7.60 – 7.43 (m, 3H), 7.45 – 7.33 (m, 2H), 7.32 – 7.20 (m, 18H), 7.16 – 7.00 (m, 2H), 5.83 (ddt, *J* = 16.3, 10.9, 5.7 Hz, 1H), 5.56 – 5.40 (m, 2H), 5.37 – 5.00 (m, 2H), 4.65 – 4.34 (m, 12H), 4.18 (ddd, *J* = 21.0, 8.5, 5.1 Hz, 2H), 3.82 (td, *J* = 9.0, 4.4 Hz, 2H), 3.56 (ddd, *J* = 20.3, 9.3, 6.6 Hz, 2H), 3.05 (dd, *J* = 13.8, 6.2 Hz, 1H), 2.93 (dd, *J* = 13.7, 7.2 Hz, 1H), 1.40 (s, 9H), 1.32 (d, *J* = 7.2 Hz, 3H), 1.11 (d, *J* = 6.5 Hz, 3H).

^{13}C NMR (75 MHz, $CDCl_3$) δ 172.2, 171.1, 170.7, 169.5, 168.5, 155.9, 155.8, 143.9, 141.3, 137.4, 137.4, 135.9, 131.6, 129.4, 128.6, 128.6, 128.5, 128.0, 127.9, 127.9, 127.9, 127.8, 127.8, 127.2, 127.2, 125.3, 125.2, 120.0, 118.8, 80.6, 73.5, 73.5, 70.6, 69.8, 69.3, 67.1, 66.0, 56.8, 55.3, 54.4, 52.6, 48.5, 47.1, 37.9, 28.4, 18.1, 16.3.

HRMS (ESI) calculated for $C_{59}H_{67}N_5O_{13}Na^+$ $[M+Na]^+$ 1076.4628, found 1076.4630.

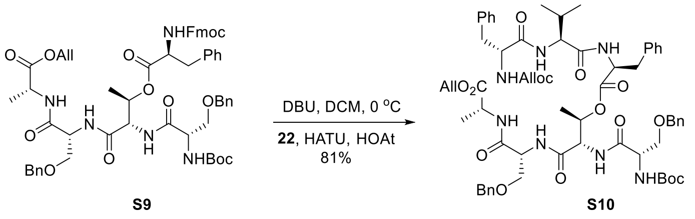

S10 was synthesized according to the procedures for the synthesis of **5** from **S9** (1.2 g, 1.1 mmol, 1.0 eq.) and **22** (440 mg, 1.3 mmol, 1.1 eq.). Purification of the crude product was performed by flash chromatography on silica gel (Hexanes/EtOAc = 2/3) to afford **S10** (1.0 g, 81%) as a white solid.

TLC: R_f = 0.6 (Hexanes/EtOAc = 2/3), UV & PMA stain.

$[\alpha]_D^{25}$ = +1.6 (*c* 1.2, MeOH).

^{1}H NMR (500 MHz, $CDCl_3$) δ 8.08 (d, *J* = 8.1 Hz, 1H), 7.52 (d, *J* = 7.4 Hz, 1H), 7.46 (d, *J* = 8.0 Hz, 1H), 7.34 – 7.31 (m, 1H), 7.30 – 7.29 (m, 3H), 7.29 – 7.26 (m, 6H), 7.25 – 7.20 (m, 6H), 7.20 – 7.15 (m, 3H), 7.14 – 7.07 (m, 1H), 6.68 (d, *J* = 7.2 Hz, 1H), 6.46 (s, 1H), 5.92 – 5.73 (m, 2H), 5.54 (d, *J* = 7.3 Hz,

1H), 5.43 (s, 1H), 5.33 – 5.26 (m, 1H), 5.25 – 5.19 (m, 2H), 5.17 – 5.10 (m, 1H), 4.83 – 4.68 (m, 2H), 4.63 – 4.49 (m, 11H), 4.35 (dd, *J* = 13.3, 5.8 Hz, 1H), 4.30 (dd, *J* = 14.7, 7.7 Hz, 1H), 4.06 (t, *J* = 6.8 Hz, 1H), 4.00 (d, *J* = 7.9 Hz, 1H), 3.70 (dd, *J* = 9.5, 5.5 Hz, 2H), 3.39 (t, *J* = 8.6 Hz, 1H), 3.23 (d, *J* = 14.0 Hz, 1H), 3.14 (dd, *J* = 13.7, 7.8 Hz, 1H), 3.00 (dd, *J* = 13.6, 7.2 Hz, 1H), 2.91 (dd, *J* = 14.0, 10.2 Hz, 1H), 2.07 – 1.95 (m, 1H), 1.43 (s, 9H), 1.33 (d, *J* = 7.2 Hz, 3H), 1.13 (d, *J* = 6.4 Hz, 3H), 0.60 (d, *J* = 6.8 Hz, 3H), 0.51 (d, *J* = 6.8 Hz, 3H).

^{13}C NMR (125 MHz, $CDCl_3$) δ 172.8, 172.1, 171.3, 171.0, 170.2, 168.4, 157.1, 155.5, 137.9, 137.5, 137.0, 136.3, 132.5, 131.7, 129.4, 129.2, 128.8, 128.6, 128.6, 128.5, 128.0, 127.9, 127.8, 127.7, 127.2, 126.9, 118.8, 118.3, 80.1, 73.4, 73.4, 70.7, 69.5, 66.3, 66.0, 59.5, 57.4, 56.3, 54.5, 54.3, 52.5, 48.5, 37.8, 36.7, 29.8, 29.3, 28.5, 19.2, 18.1, 17.3, 16.1.

HRMS (ESI) calculated for $C_{62}H_{79}N_7O_{15}Na^+$ $[M+Na]^+$ 1184.5526, found 1184.5520.

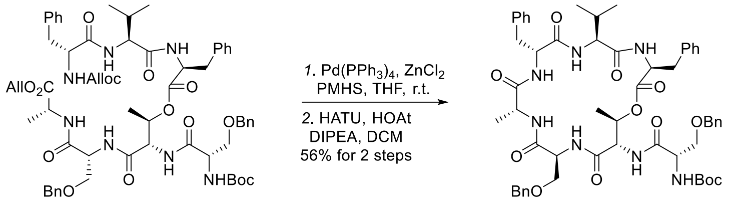

S10 **S11**

S11 was synthesized according to the procedures for the synthesis of **2** from **S10** (500 mg, 0.43 mmol, 1.0 eq.). Purification of the crude product was performed by flash chromatography on silica gel (Hexanes/EtOAc = 2/1) to afford **S11** (248 mg, 56% for 2 steps) as a white solid.

TLC: R_f = 0.5 (Hexanes/EtOAc = 1/1), UV & PMA stain.

$[\alpha]_D^{26}$ = +19.6 (*c* 2.8, $CHCl_3$).

^{1}H NMR (400 MHz, $CDCl_3$) δ 7.71 – 7.64 (m, 1H), 7.58 (d, *J* = 9.1 Hz, 1H), 7.56 – 7.52 (m, 1H), 7.52 – 7.46 (m, 1H), 7.38 – 7.30 (m, 3H), 7.29 – 7.19 (m, 12H), 7.20 – 7.09 (m, 5H), 6.86 (d, *J* = 8.8 Hz, 1H), 6.09 (s, 1H), 5.56 (s, 2H), 5.12 – 4.93 (m, 2H), 4.65 (dt, *J* = 9.6, 4.9 Hz, 1H), 4.59 (d, *J* = 11.4 Hz, 1H), 4.57 – 4.44 (m, 4H), 4.35 – 4.25 (m, 1H), 4.14 (t, *J* = 9.6 Hz, 1H), 4.11 – 4.00 (m, 2H), 3.88 (dd, *J* = 9.9, 5.6 Hz, 1H), 3.80 (t, *J* = 7.9 Hz, 1H), 3.68 (dd, *J* = 9.3, 4.1 Hz, 1H), 3.42 – 3.18 (m, 3H), 3.02 (dd, *J* = 13.9, 10.9 Hz, 1H), 2.20 – 2.12 (m, 1H), 1.35 (s, 9H), 1.12 (d, *J* = 6.3 Hz, 3H), 1.02 (d, *J* = 7.2 Hz, 3H), 0.76 (d, *J* = 6.9 Hz, 3H), 0.35 (d, *J* = 6.9 Hz, 3H).

^{13}C NMR (100 MHz, $CDCl_3$) δ 173.4, 172.9, 171.8, 171.6, 170.9, 170.5, 169.7, 155.6, 137.8, 137.4, 136.6, 132.3, 132.2, 129.4, 129.3, 128.9, 128.6, 128.6, 128.4, 128.1, 127.9, 127.8, 126.8, 126.6, 80.1, 74.0, 73.4, 71.5, 70.2, 68.5, 58.7, 55.4, 55.1, 53.2, 51.1, 36.6, 35.1, 32.0, 29.8, 29.7, 28.4, 19.5, 16.9, 16.3, 16.2.

HRMS (ESI) calculated for $C_{55}H_{70}N_7O_{12}^+$ $[M+H]^+$ 1020.5077, found 1020.5080.

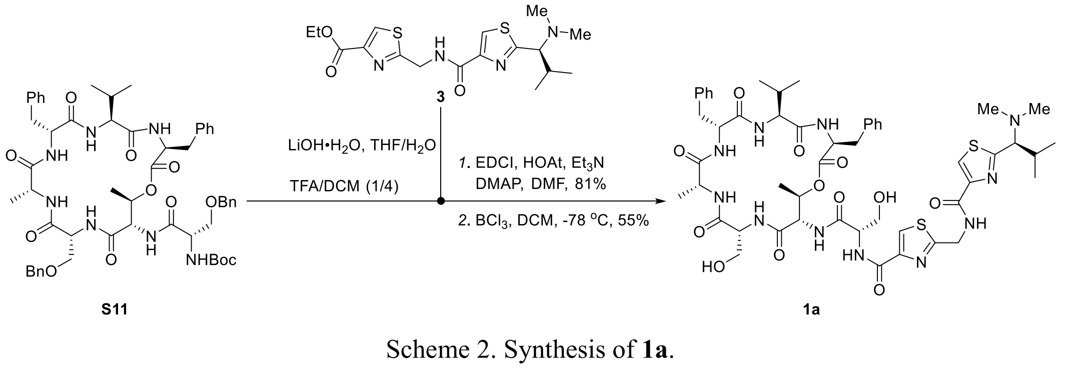

S11 **1a**

Scheme 2. Synthesis of **1a**.

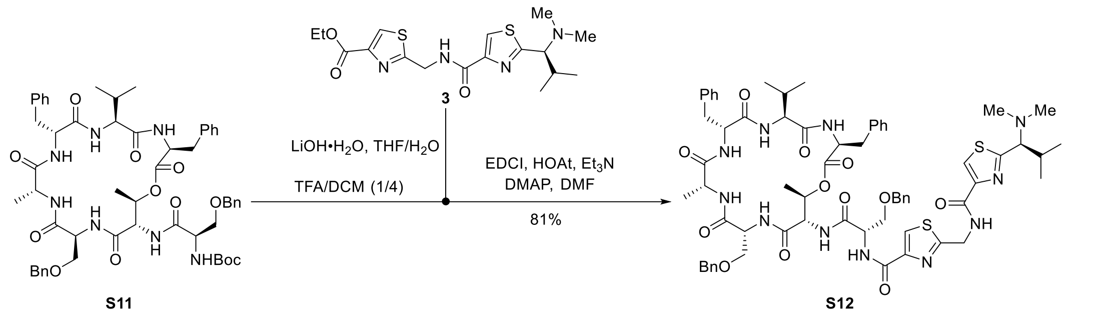

S12 was synthesized according to the procedures for the synthesis of **S4** from **S11** (90 mg, 0.09 mmol, 1.0 eq.) and **3** (142.5 mg, 0.36 mmol, 4.0 eq.). Purification of the crude product was performed by flash chromatography on silica gel (MeOH/DCM = 1/40-1/20) to afford **S12** (93 mg, 81%) as a white solid.

TLC: R_f = 0.4 (MeOH/DCM = 1/10), UV & PMA stain.

$[\alpha]_D^{27}$ = +20.2 (*c* 0.45, $CHCl_3$).

^{1}H NMR (300 MHz, $CDCl_3$) δ 8.24 – 8.10 (m, 3H), 8.03 (s, 1H), 7.93 (d, *J* = 8.8 Hz, 1H), 7.52 (t, *J* = 7.9 Hz, 2H), 7.42 (d, *J* = 4.0 Hz, 1H), 7.37 – 7.29 (m, 3H), 7.26 (s, 1H), 7.25 – 7.23 (m, 2H), 7.22 (s, 4H), 7.20 (s, 5H), 7.17 – 7.12 (m, 4H), 7.12 – 7.09 (m, 1H), 6.99 (d, *J* = 8.4 Hz, 1H), 6.83 (d, *J* = 9.0 Hz, 1H), 5.68 – 5.53 (m, 1H), 5.11 – 5.03 (m, 2H), 5.02 – 4.97 (m,1H), 4.99 – 4.86 (m, 01H), 4.90 – 4.76 (m, 1H), 4.58 (d, *J* = 11.3 Hz, 2H), 4.53 (s, 2H), 4.49 (d, *J* = 11.4 Hz, 1H), 4.19 – 4.16 (m, 1H), 4.15 – 4.12 (m, 1H), 4.12 – 4.03 (m, 3H), 3.92 (dd, *J* = 9.8, 4.8 Hz, 1H), 3.71 – 3.58 (m, 1H), 3.43 (d, *J* = 9.1 Hz, 1H), 3.31 (dd, *J* = 13.7, 4.8 Hz, 1H), 3.26 – 3.19 (m, 1H), 3.13 (dd, *J* = 14.2, 5.5 Hz, 1H), 2.98 (dd, *J* = 13.9, 11.1 Hz, 1H), 2.21 (s, 6H), 2.17 – 2.13 (m, 2H), 1.18 (d, *J* = 6.2 Hz, 3H), 1.07 (d, *J* = 7.2 Hz, 3H), 1.02 (d, *J* = 6.6 Hz, 3H), 0.80 (d, *J* = 6.6 Hz, 3H), 0.73 (d, *J* = 6.9 Hz, 3H), 0.30 (d, *J* = 6.9 Hz, 3H).

^{13}C NMR (75 MHz, $CDCl_3$) δ 173.2, 172.3, 171.7, 171.1, 170.7, 170.5, 169.7, 169.4, 168.2, 161.7, 160.9, 149.4, 148.5, 137.9, 137.5, 137.4, 136.5, 129.4, 129.2, 128.9, 128.7, 128.6, 128.5, 128.4, 128.1, 127.7, 127.6, 126.7, 126.6, 124.6, 123.8, 74.0, 73.3, 73.1, 71.2, 69.9, 68.4, 58.6, 55.4, 53.6, 53.3, 53.2, 53.2, 51.3, 41.9, 41.9, 41.0, 36.6, 35.0, 30.2, 29.5, 20.3, 19.5, 19.4, 17.0, 16.5, 16.0.

HRMS (ESI) calculated for $C_{65}H_{80}N_{11}O_{12}S_2Na^+$ $[M+Na]^+$ 1270.5424, found 1270.5420.

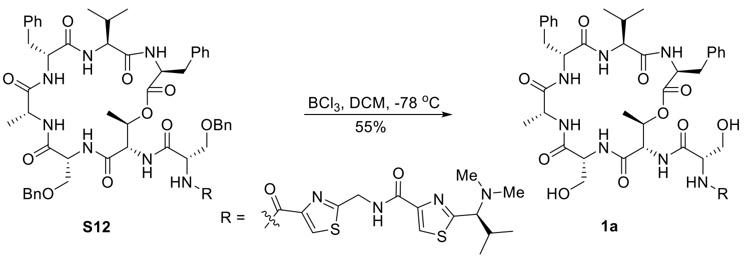

1a was synthesized according to the procedures for the synthesis of pagoamide A (**1**) from **S12** (32 mg, 0.025 mmol, 1.0 eq.). Purification of the crude product was performed by flash chromatography on silica gel (MeOH/DCM = 1/40-1/20) to afford **1a** (15 mg, 55%) as a white solid.

TLC: R_f = 0.2 (MeOH/DCM = 1/10), UV & PMA stain.

$[\alpha]_D^{27}$ = +22.0 (*c* 0.1, MeOH).

^{1}H NMR (500 MHz, DMSO-d_6) δ 9.28 (t, *J* = 6.2 Hz, 1H), 8.94 (s, 1H), 8.49 (s, 1H), 8.30 (s, 1H), 8.18 (s, 1H), 8.14 (d, *J* = 7.9 Hz, 1H), 8.03 (s, 1H), 7.97 (s, 1H), 7.33 – 7.23 (m, 2H), 7.26 – 7.17 (m, 7H), 7.20 – 7.13 (m, 2H), 6.93 (d, *J* = 9.0 Hz, 1H), 5.84 – 5.67 (m, 1H), 5.41 (t, *J* = 7.8 Hz, 1H), 5.12 (d, *J* = 10.8 Hz, 1H), 5.11 (d, *J* = 12.1 Hz, 1H), 5.01 (d, *J* = 10.4 Hz, 1H), 4.84 – 4.77 (m, 1H), 4.80 – 4.73 (m, 2H), 4.59 (d, *J* = 7.1 Hz, 1H), 4.44 (dd, *J* = 7.8, 4.1 Hz, 1H), 4.39 – 4.31 (m, 1H), 4.28 – 4.19 (m, 1H),

4.22 – 4.12 (m, 1H), 3.73 (dt, *J* = 10.5, 5.1 Hz, 1H), 3.65 (dt, *J* = 10.7, 5.0 Hz, 1H), 3.53 – 3.47 (m, 2H), 3.16 (dd, *J* = 14.6, 8.1 Hz, 1H), 3.08 (dd, *J* = 13.5, 6.7 Hz, 1H), 2.91 – 2.80 (m, 2H), 2.32 – 2.21 (m, 1H), 2.16 (s, 6H), 1.93 – 1.82 (m, 1H), 1.02 (d, *J* = 6.7 Hz, 3H), 1.00 (d, *J* = 6.5 Hz, 3H), 0.79 (d, *J* = 6.6 Hz, 3H), 0.75 (d, *J* = 6.5 Hz, 3H), 0.72 (d, *J* = 6.6 Hz, 3H), 0.69 (d, *J* = 6.5 Hz, 3H).

^{13}C NMR (125 MHz, DMSO-d_6) δ 174.8, 171.1, 170.4, 170.0, 169.0, 168.9, 168.7, 168.3, 166.9, 161.1, 159.9, 148.7, 148.4, 137.6, 135.9, 128.9, 128.7, 128.2, 128.0, 126.7, 126.1, 124.5, 124.3, 71.9, 67.3, 61.8, 60.8, 55.8, 55.6, 54.5, 54.5, 45.9, 45.2, 41.2, 40.8, 35.6, 32.9, 31.6, 29.4, 20.0, 19.3, 18.8, 17.7, 17.6, 17.0.

HRMS (ESI) calculated for $C_{51}H_{67}N_{11}O_{12}S_2Na^+$ $[M+Na]^+$ 1112.4304, found 1112.4308.

3. Comparison of NMR Spectra of Natural and Synthetic Pagoamide A

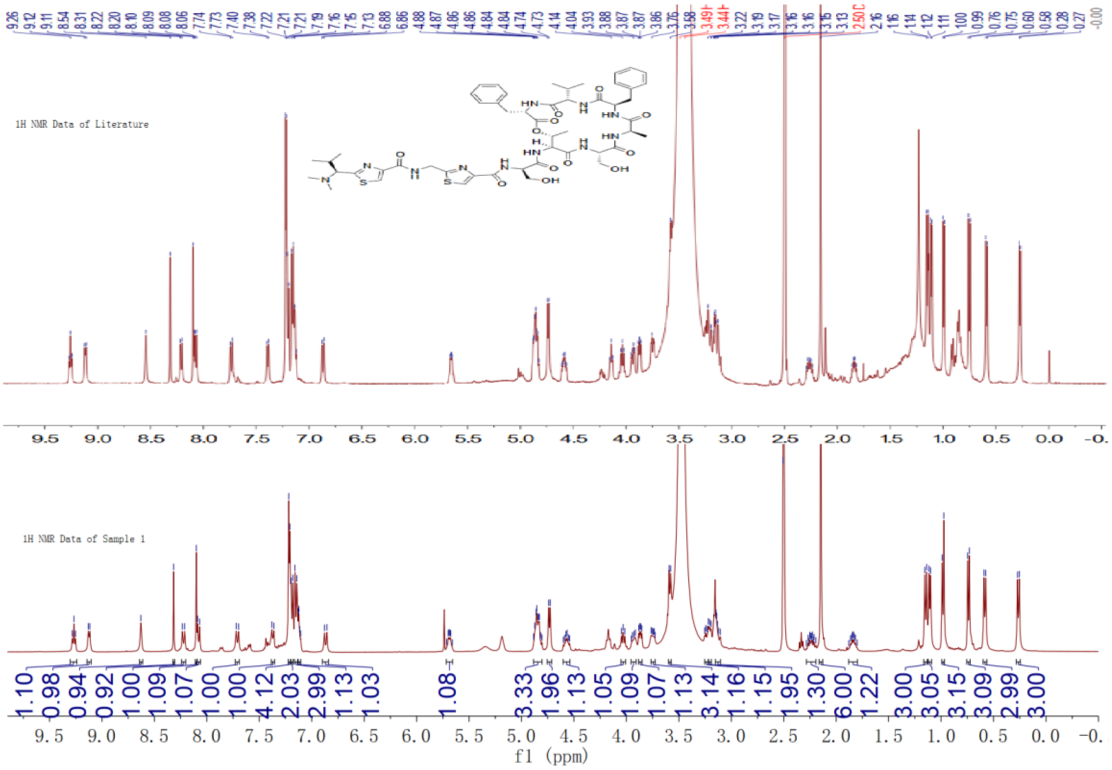

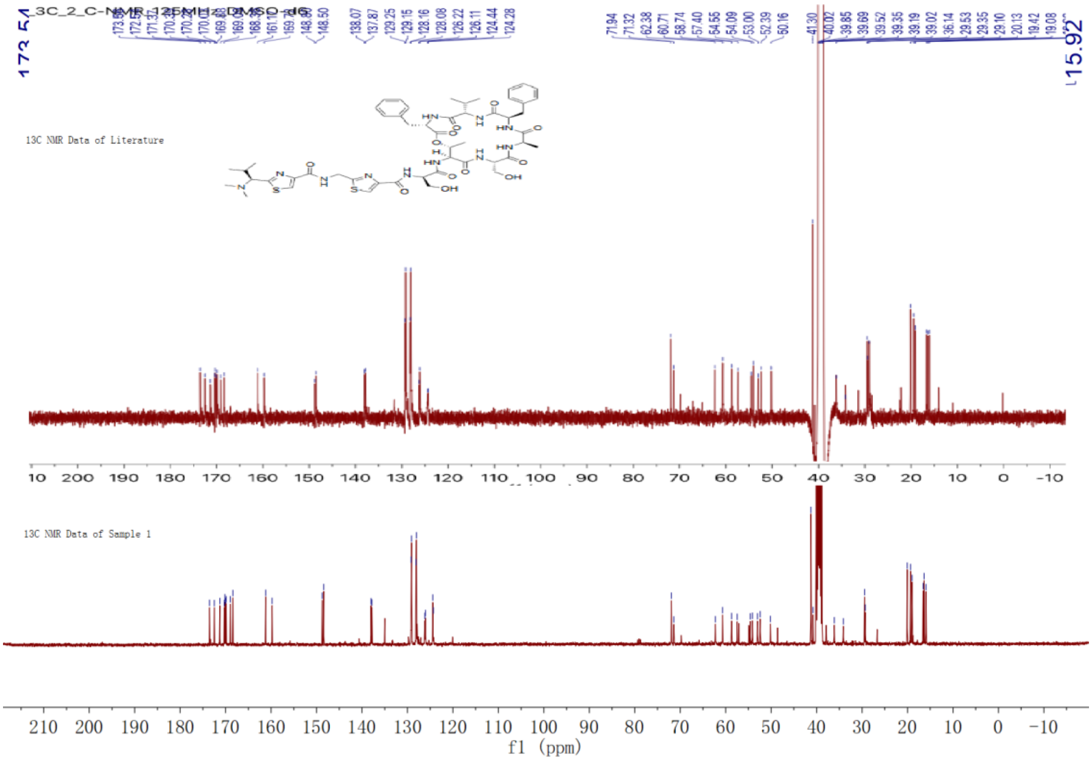

4. NMR spectra

^{1}H NMR Spectrum of **7** (300 MHz, DMSO-d_6)

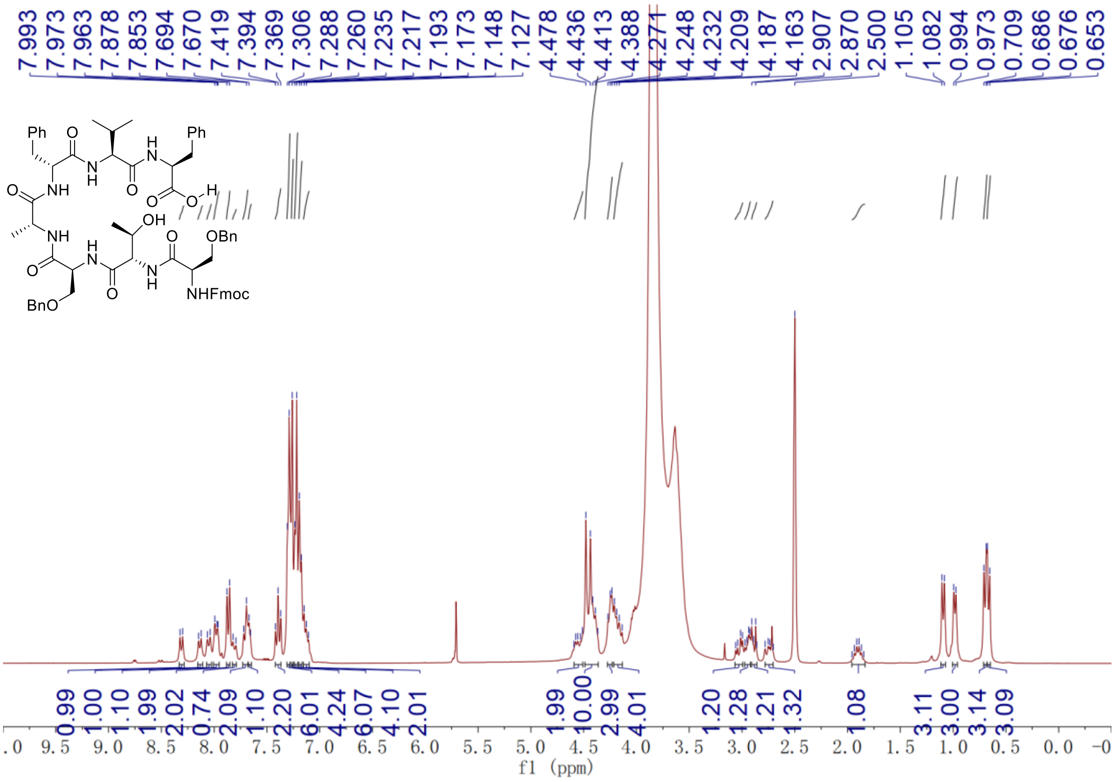

^{13}C NMR Spectrum of **7** (75 MHz, DMSO-d_6)

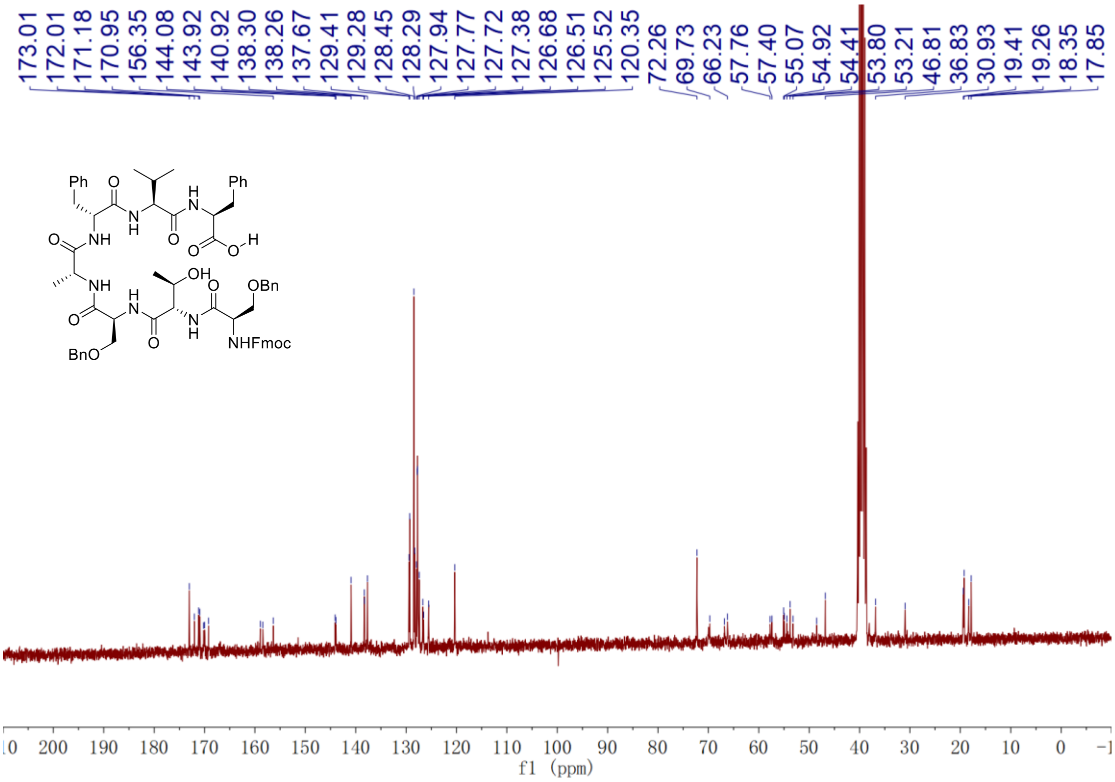

^{1}H NMR Spectrum of **8** (400 MHz, $CDCl_3$)

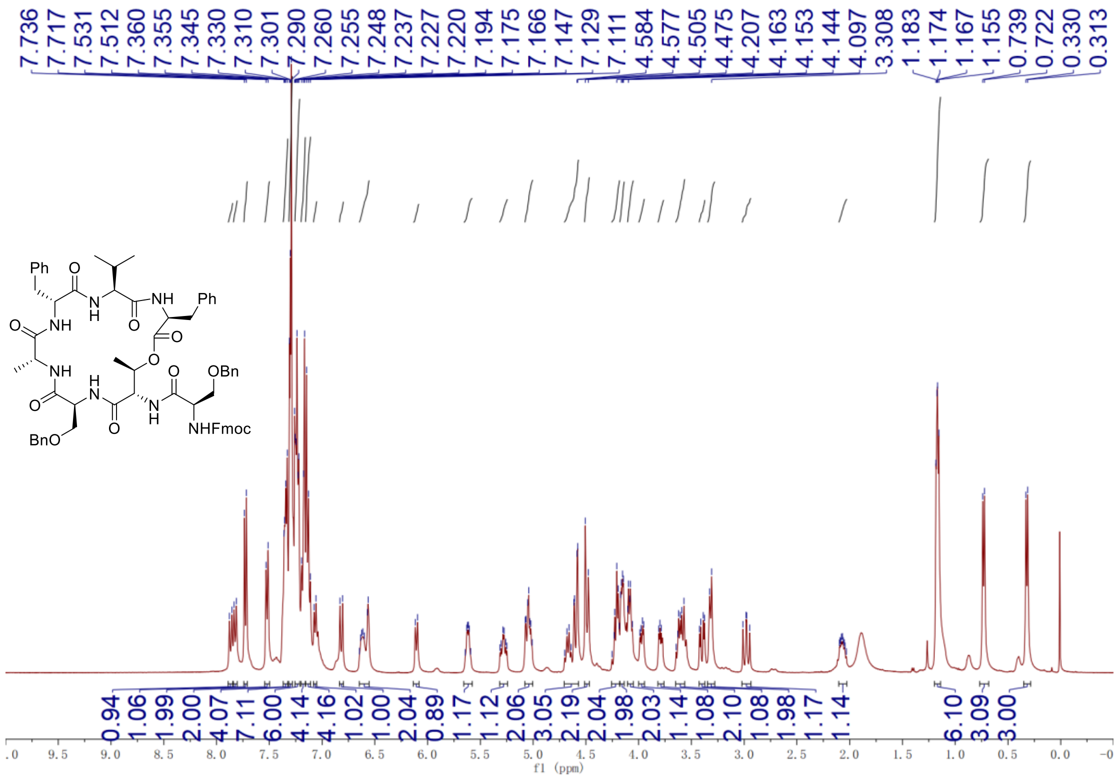

^{13}C NMR Spectrum of **8** (100 MHz, $CDCl_3$)

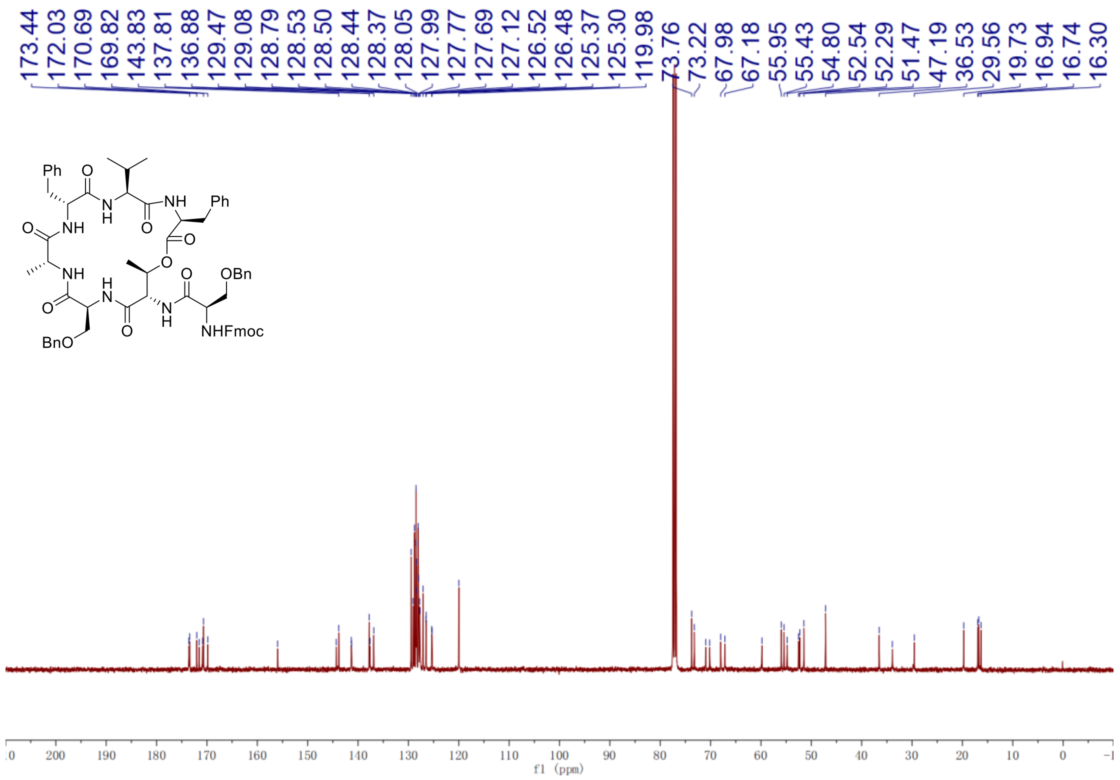

^{1}H NMR Spectrum of **11** (400 MHz, $CDCl_3$)

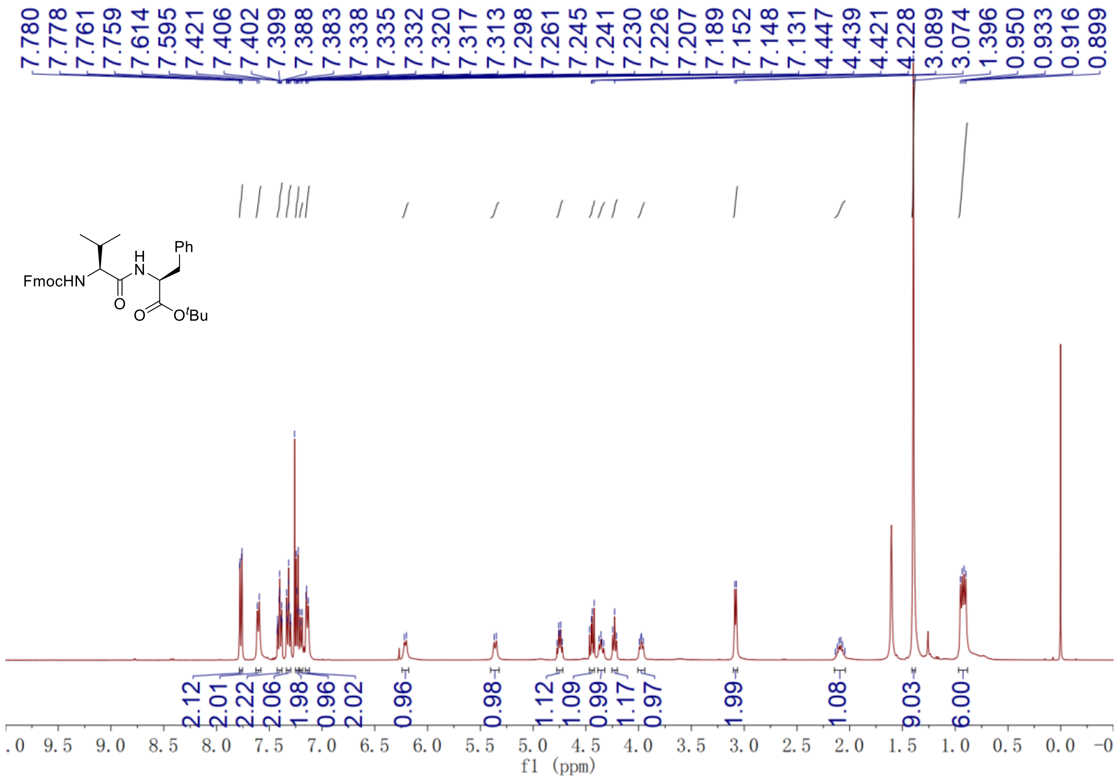

^{13}C NMR Spectrum of **11** (100 MHz, $CDCl_3$)

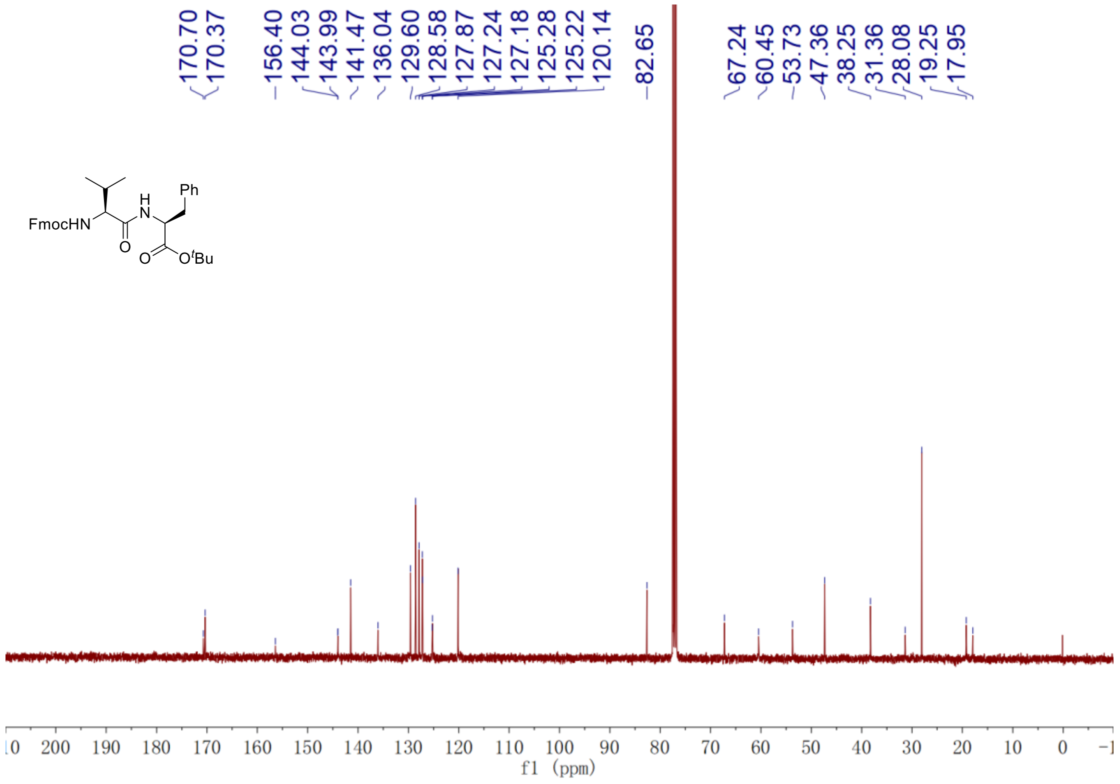

^{1}H NMR Spectrum of **12** (500 MHz, $CDCl_3$)

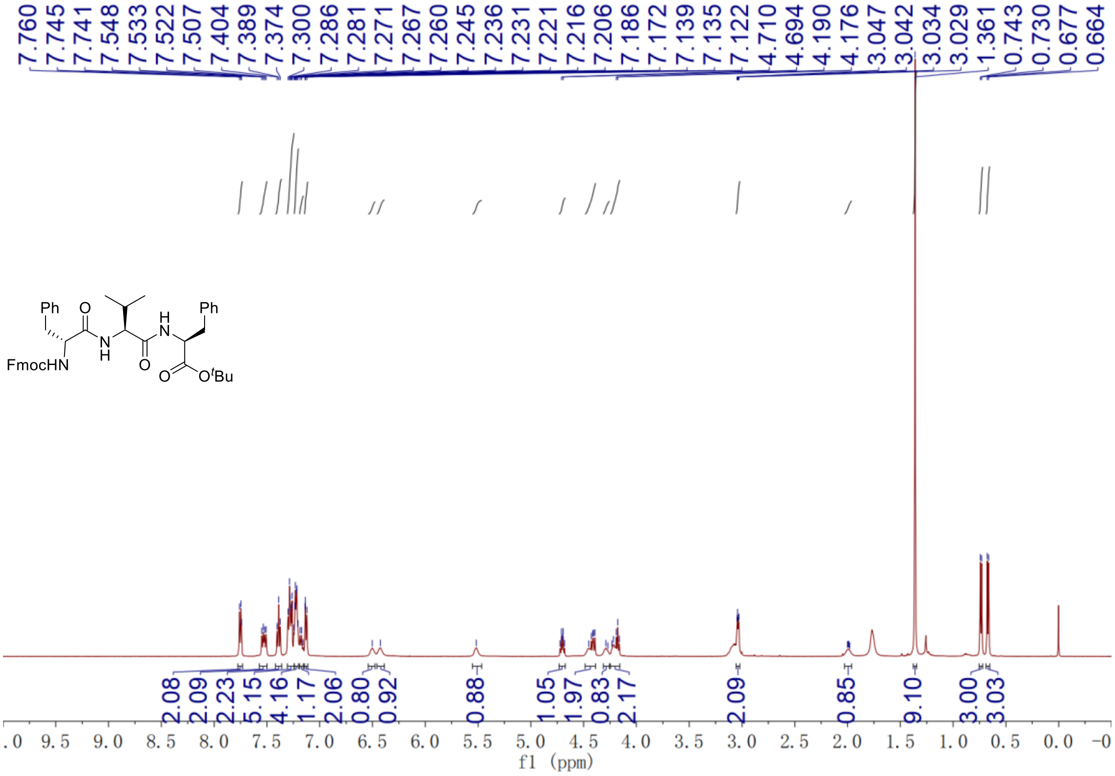

^{13}C NMR Spectrum of **12** (125 MHz, $CDCl_3$)

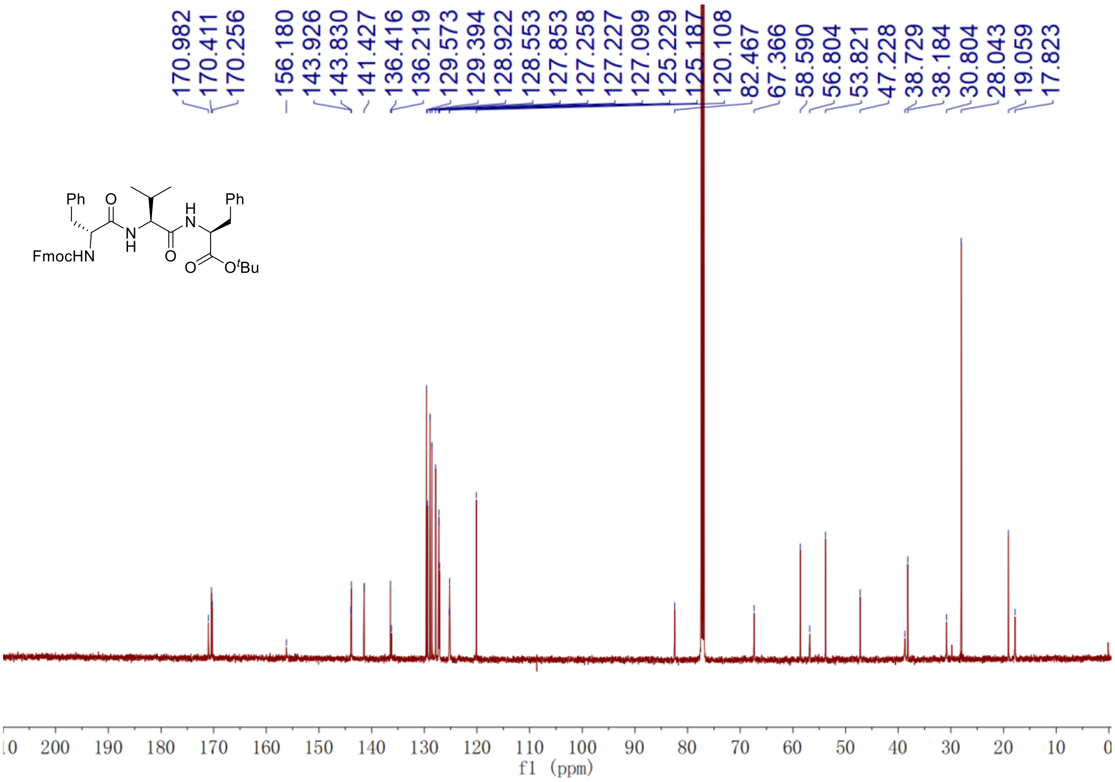

^{1}H NMR Spectrum of **13** (500 MHz, $CDCl_3$)

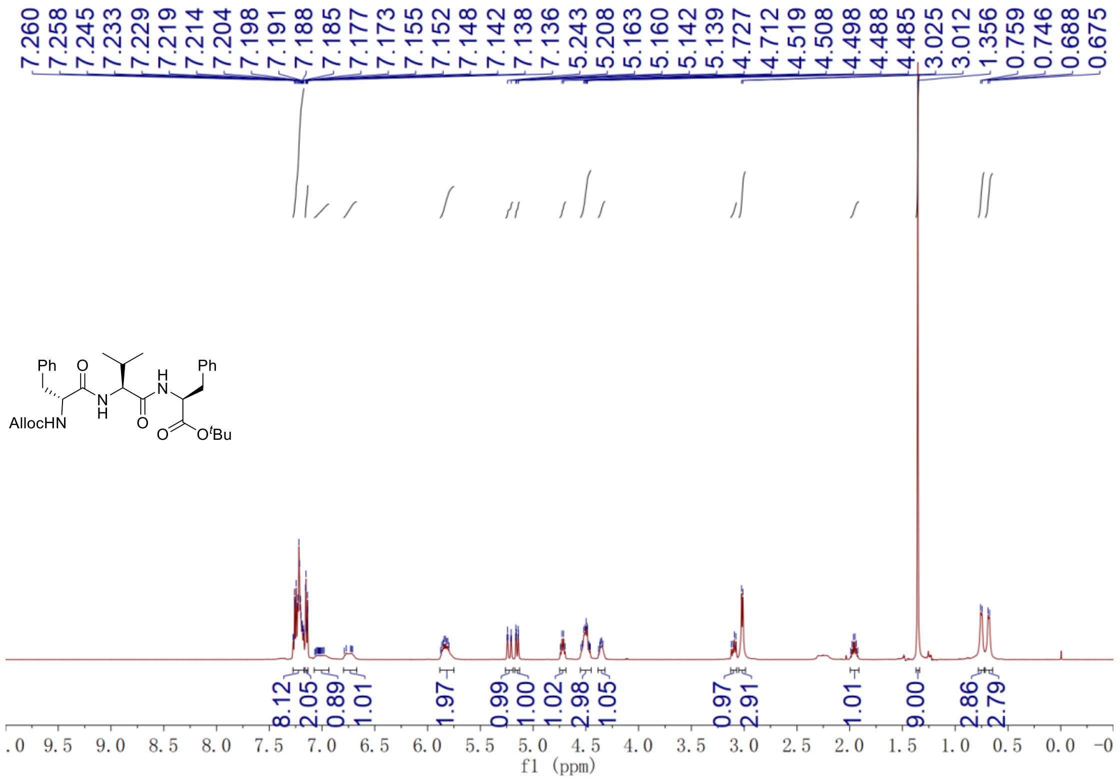

^{13}C NMR Spectrum of **13** (125 MHz, $CDCl_3$)

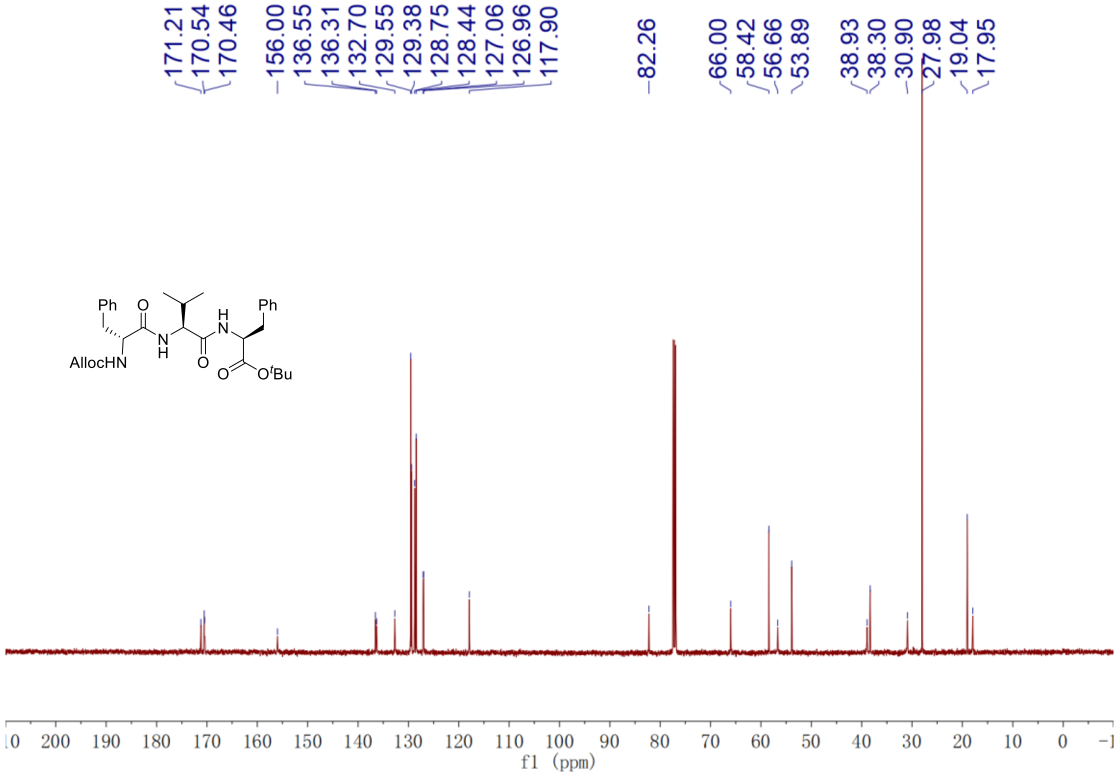

^{1}H NMR Spectrum of **14** (400 MHz, DMSO-d_6)

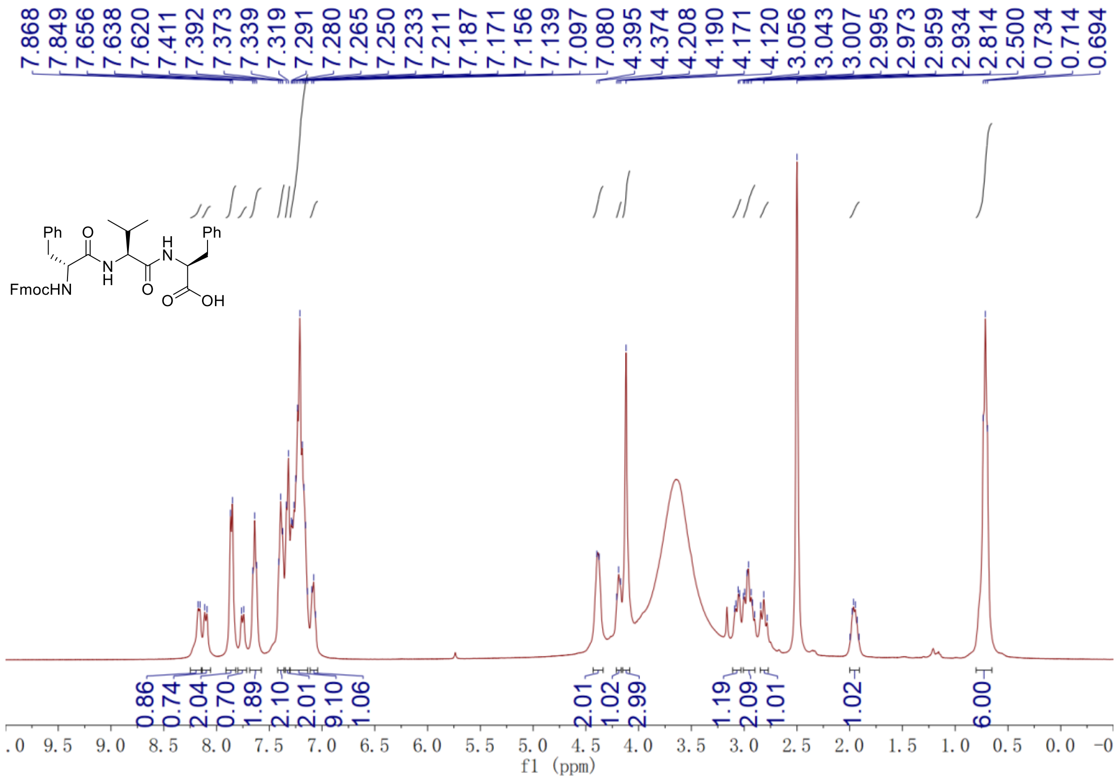

^{13}C NMR Spectrum of **14** (100 MHz, DMSO-d_6)

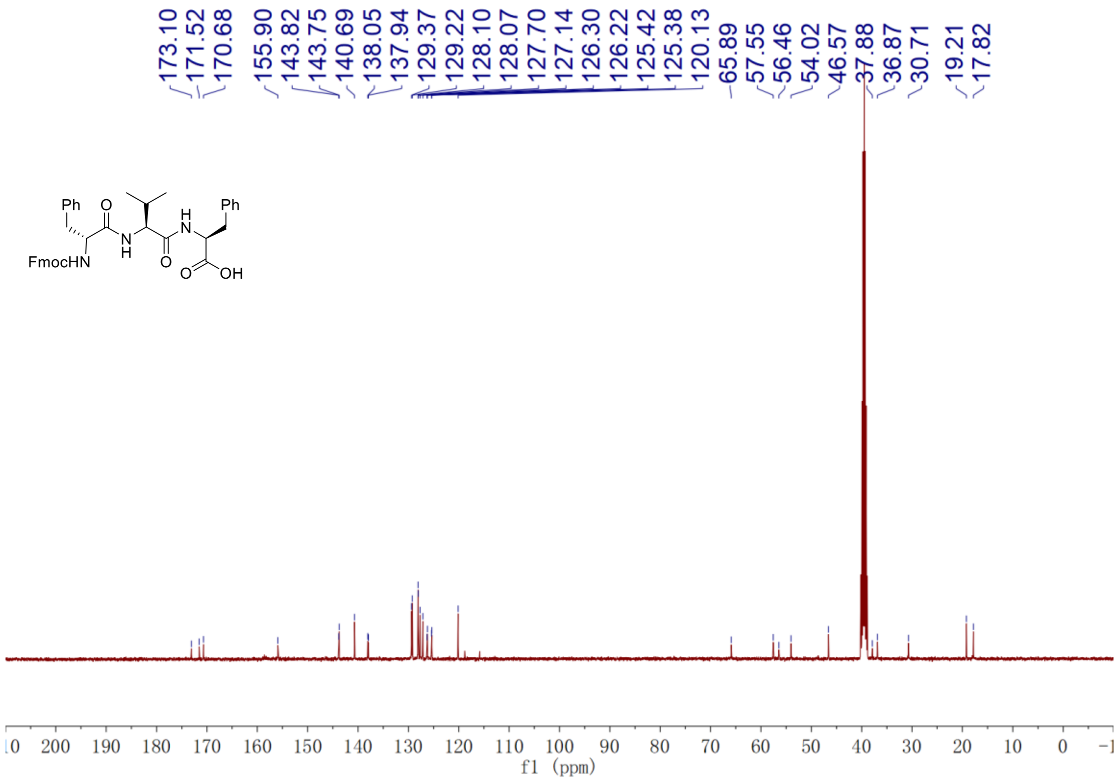

^{1}H NMR Spectrum of **15** (400 MHz, DMSO-d_6)

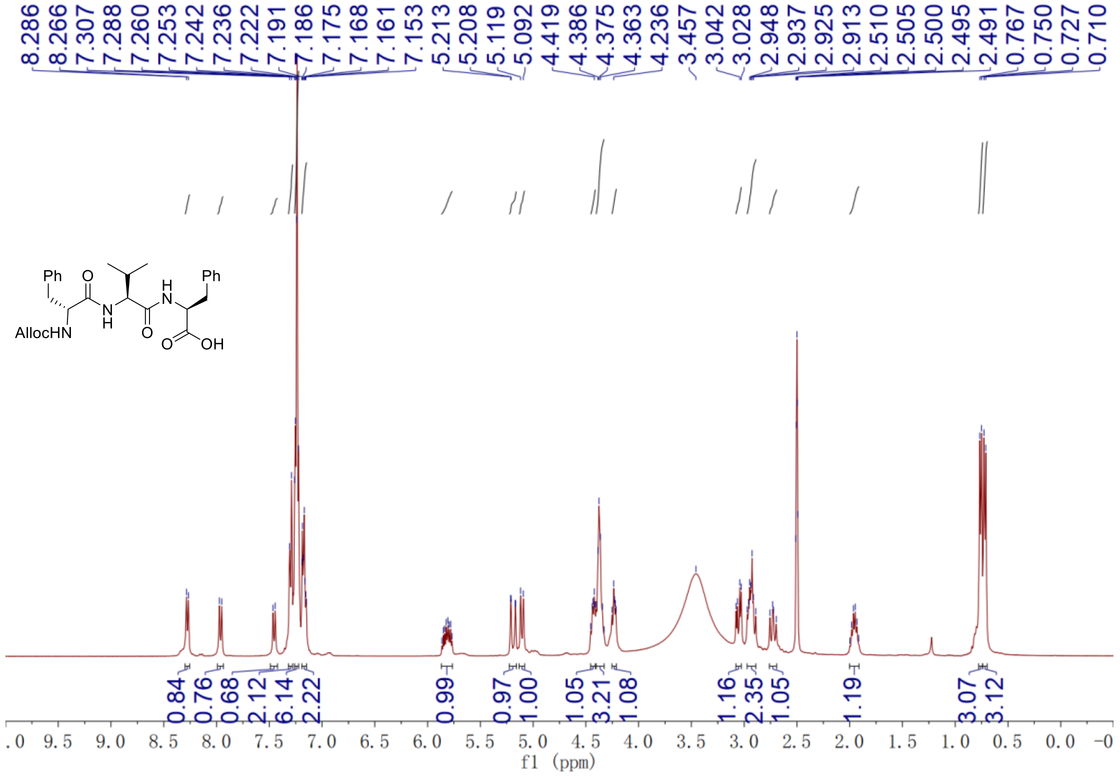

^{13}C NMR Spectrum of **15** (100 MHz, DMSO-d_6)

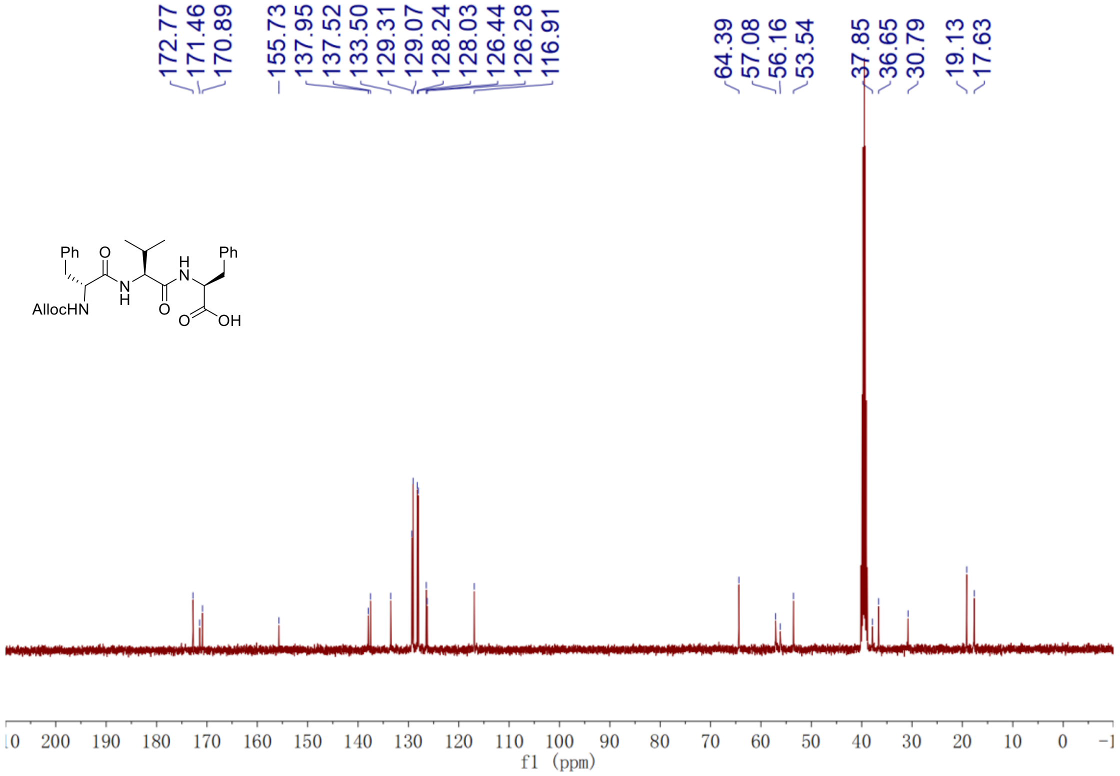

^{1}H NMR Spectrum of **18** (500 MHz, $CDCl_3$)

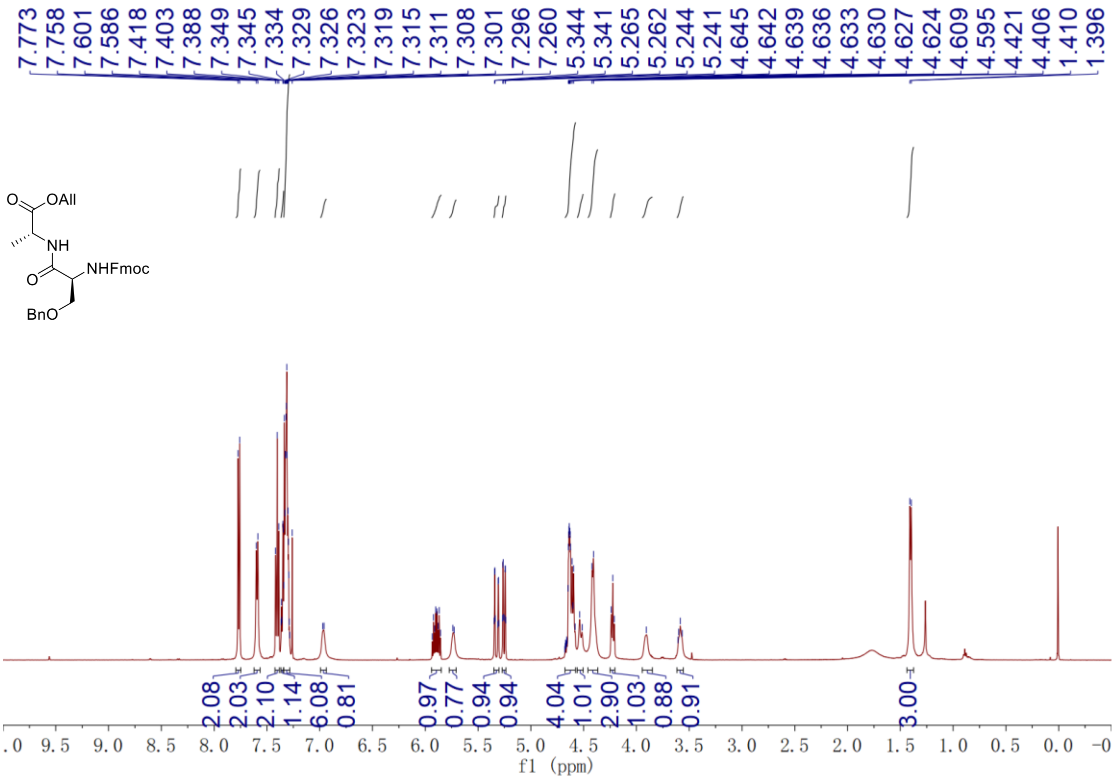

^{13}C NMR Spectrum of **18** (125 MHz, $CDCl_3$)

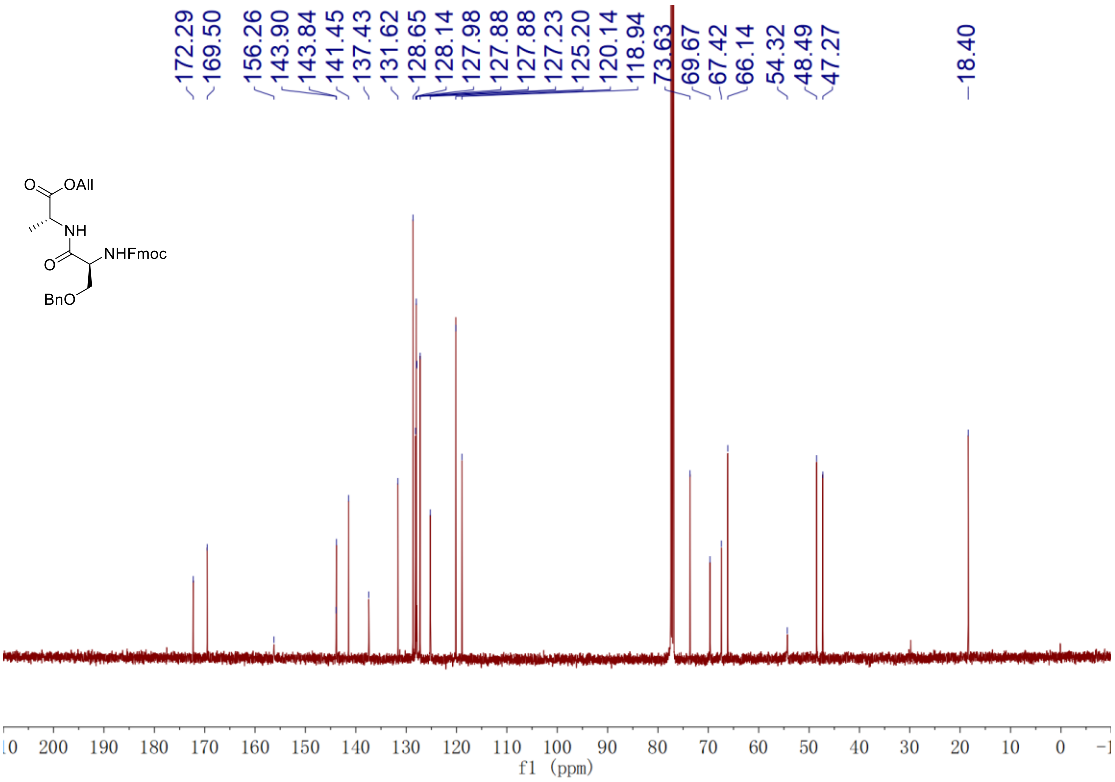

^{1}H NMR Spectrum of **19** (500 MHz, $CDCl_3$)

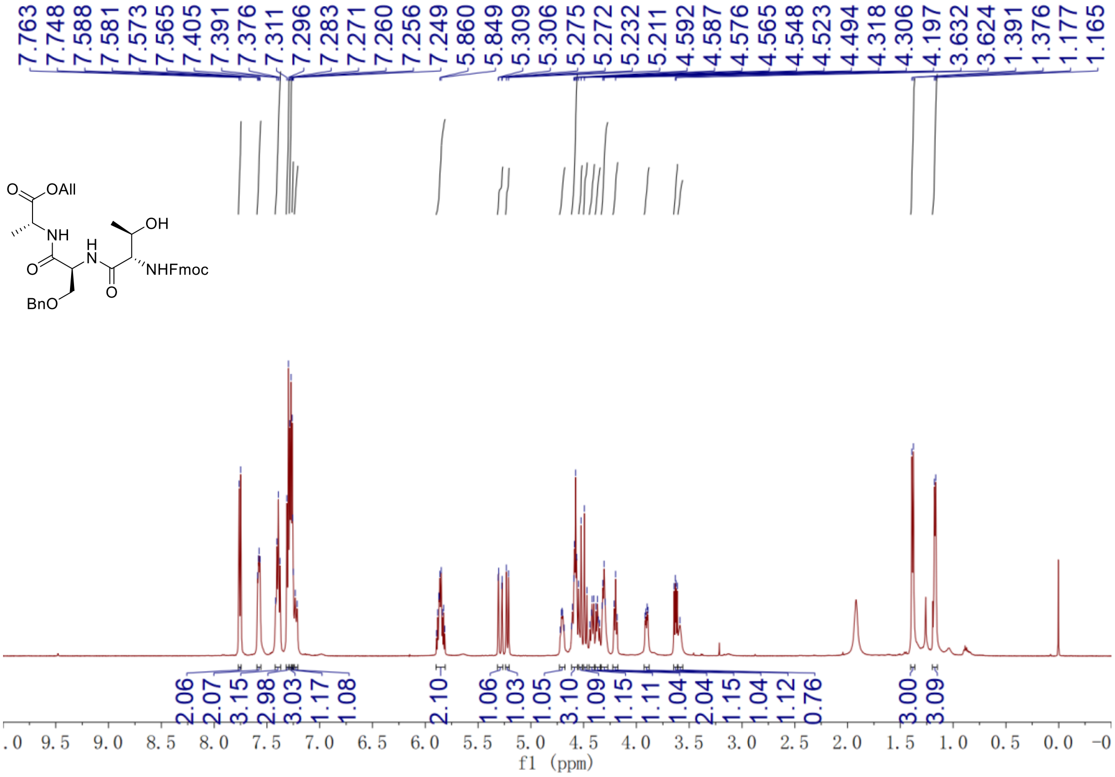

^{13}C NMR Spectrum of **19** (125 MHz, $CDCl_3$)

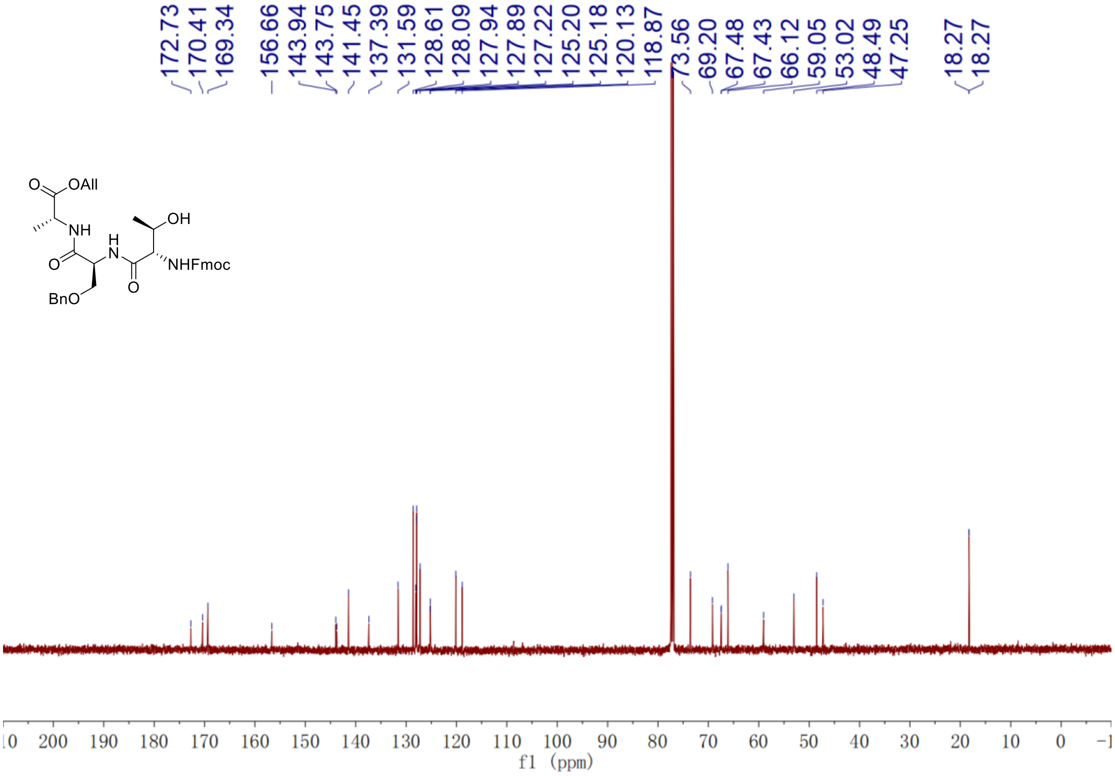

^{1}H NMR Spectrum of **20** (500 MHz, $CDCl_3$)

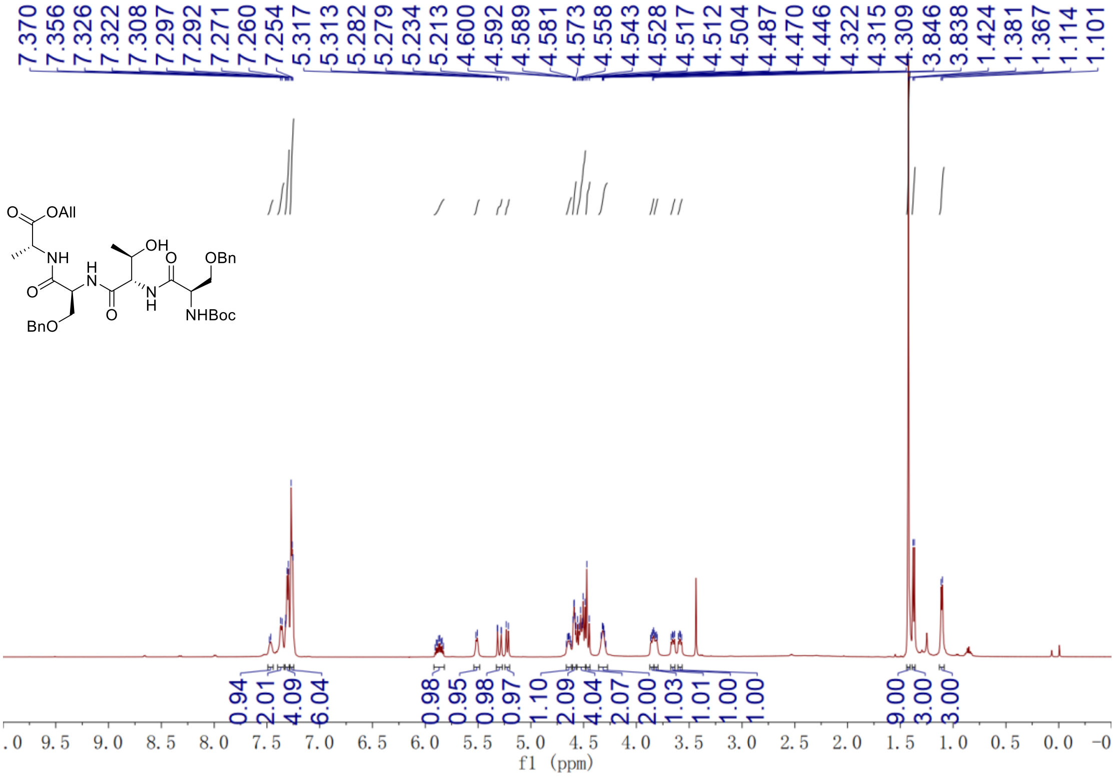

^{13}C NMR Spectrum of **20** (125 MHz, $CDCl_3$)

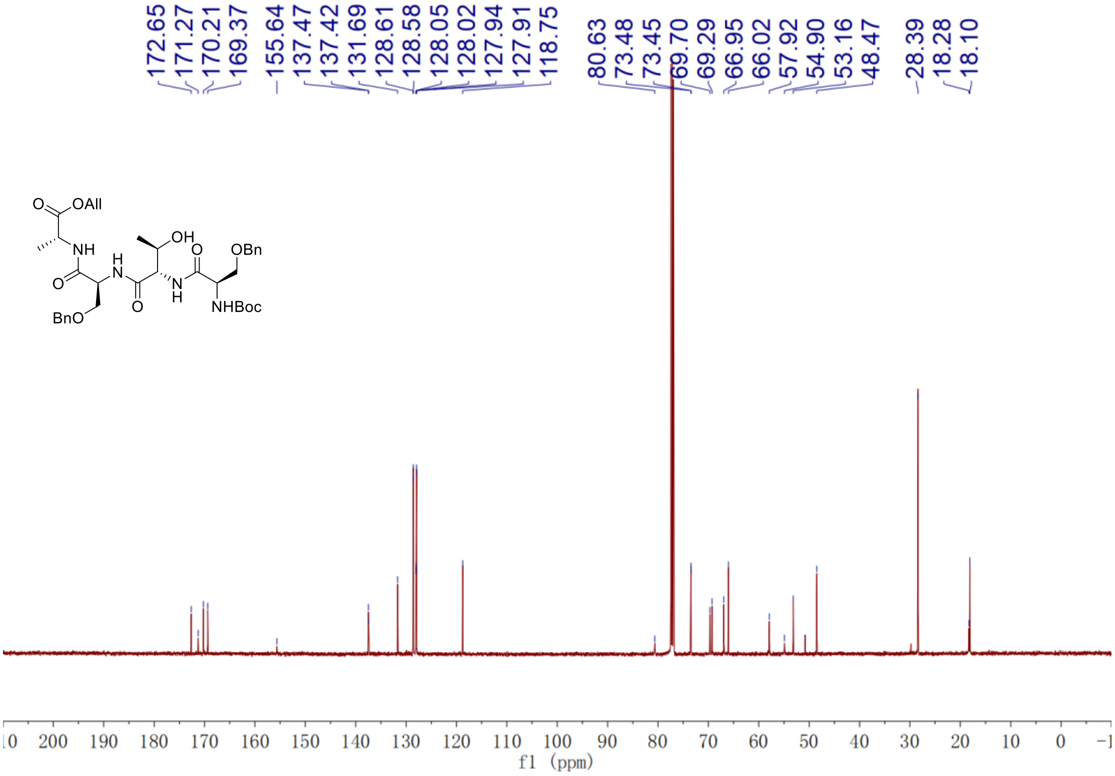

^{1}H NMR Spectrum of **21** (400 MHz, $CDCl_3$)

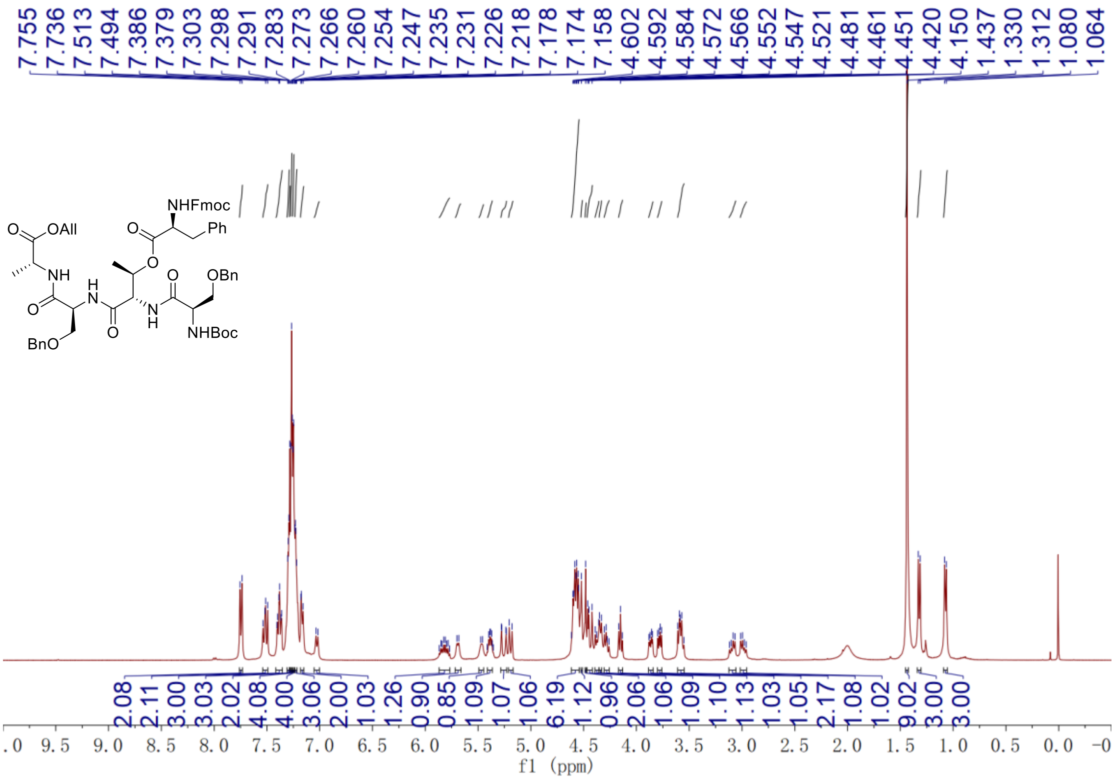

^{13}C NMR Spectrum of **21** (100 MHz, $CDCl_3$)

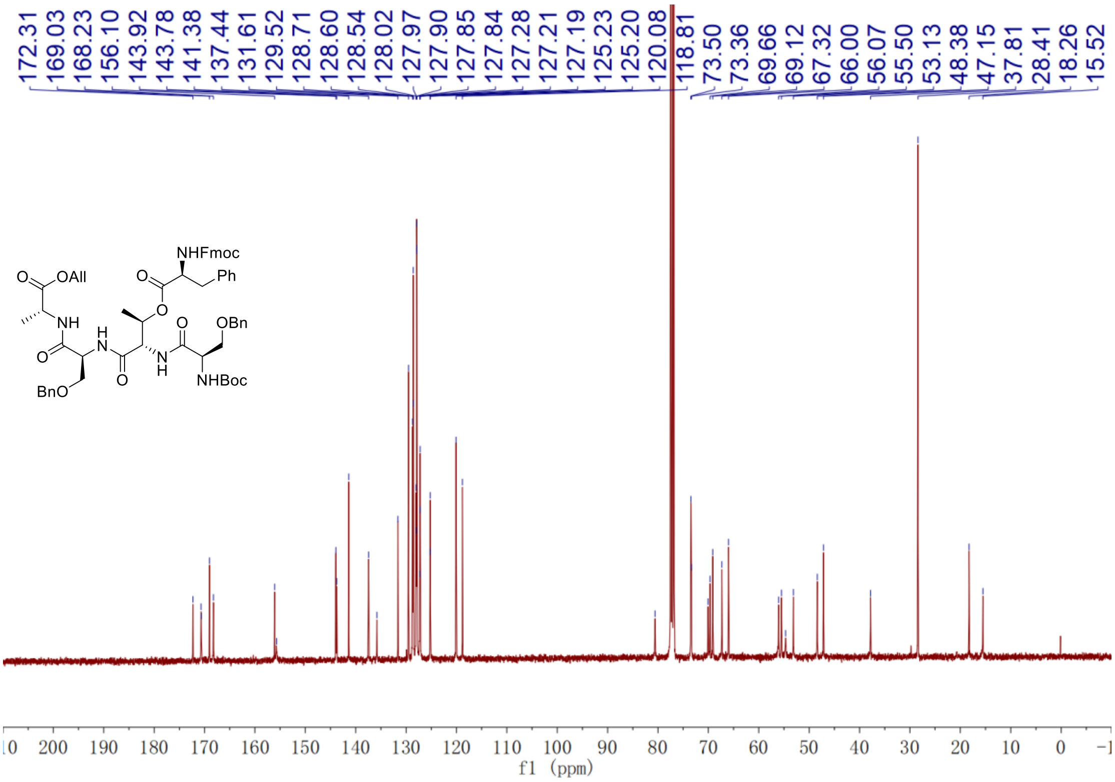

^{1}H NMR Spectrum of **S1** (300 MHz, $CDCl_3$)

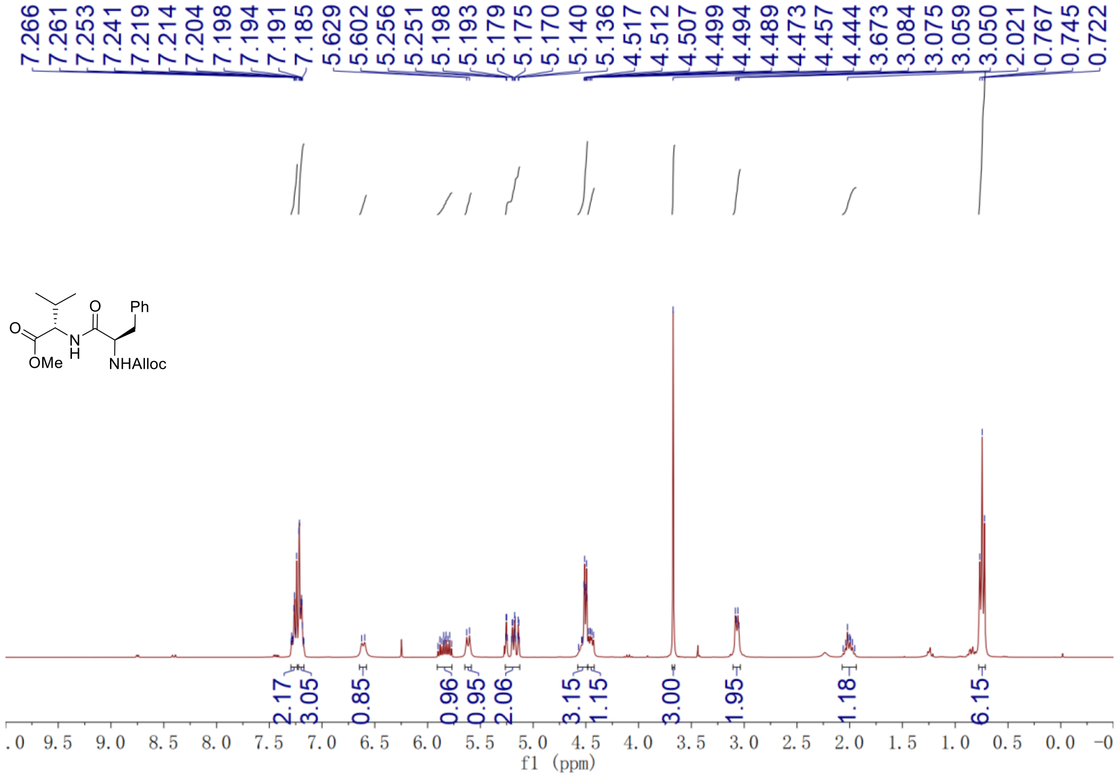

^{13}C NMR Spectrum of **S1** (75 MHz, $CDCl_3$)

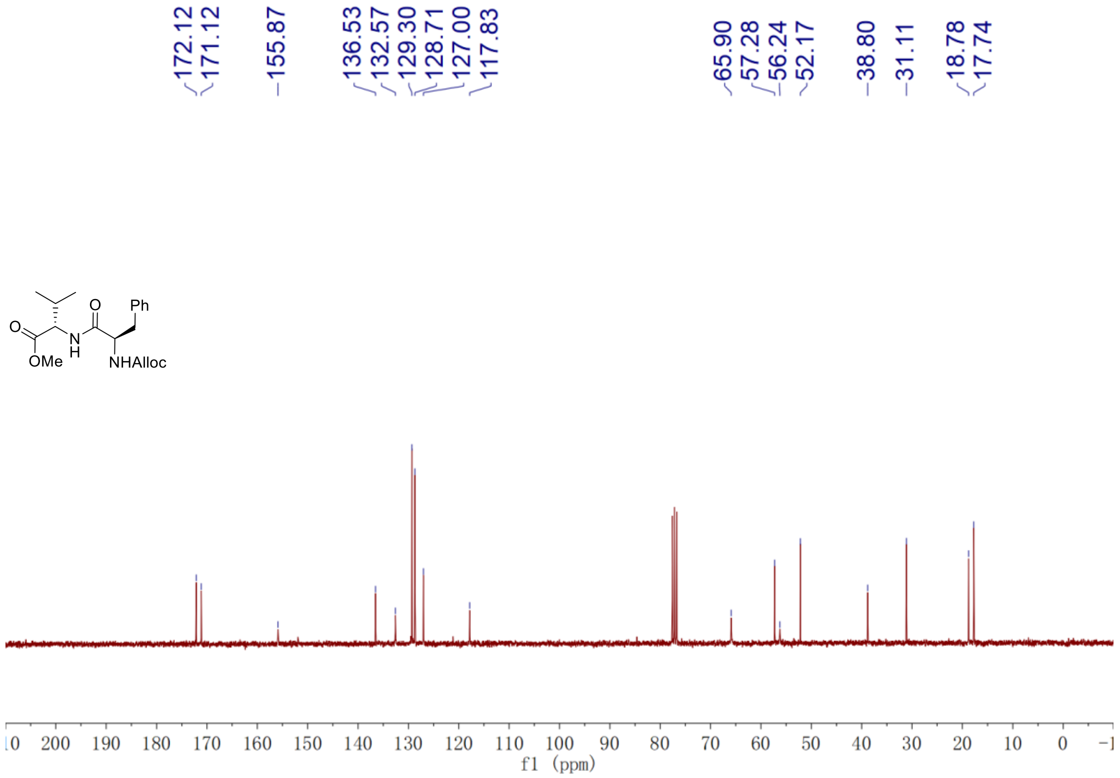

^{1}H NMR Spectrum of **22** (500 MHz, $CDCl_3$)

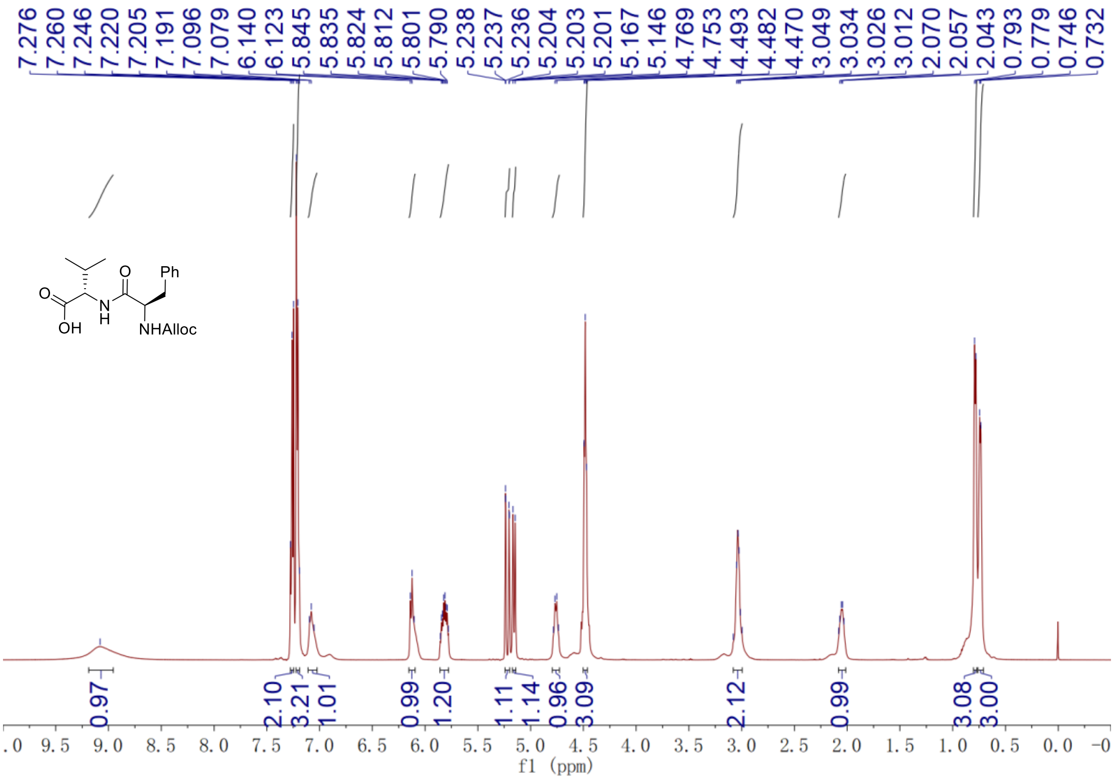

^{13}C NMR Spectrum of **22** (125 MHz, $CDCl_3$)

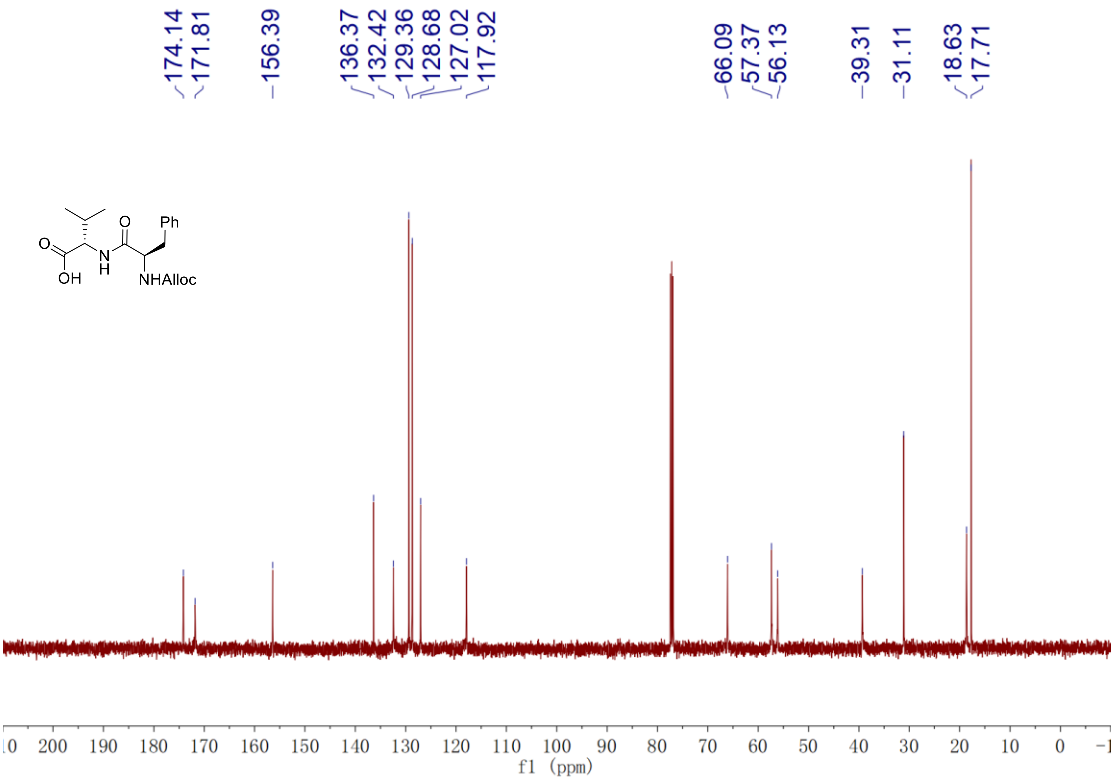

^{1}H NMR Spectrum of **5** (400 MHz, $CDCl_3$)

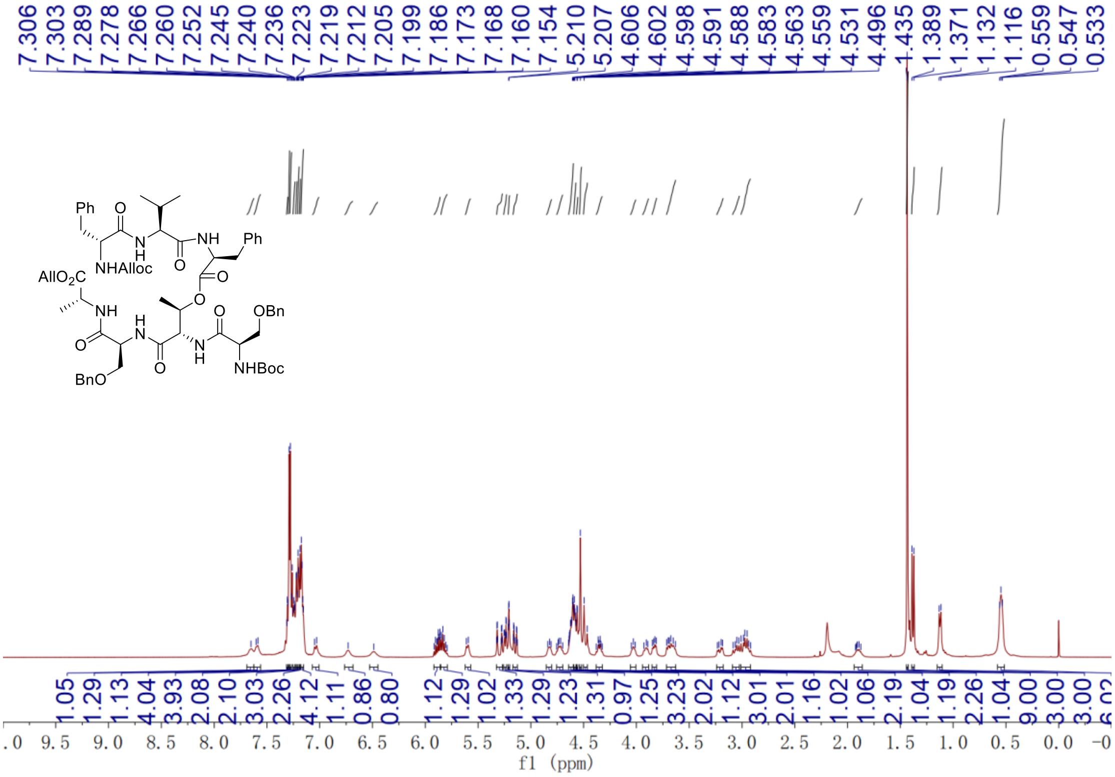

^{13}C NMR Spectrum of **5** (100 MHz, $CDCl_3$)

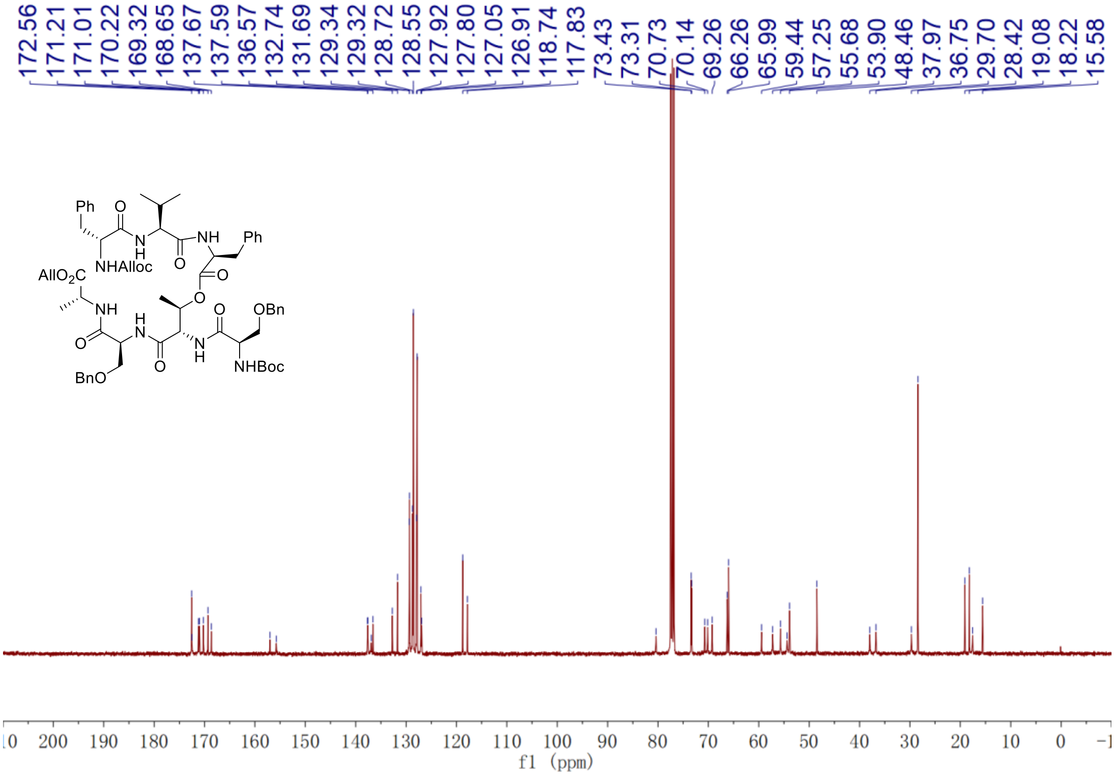

^{1}H NMR Spectrum of **S2** (300 MHz, $CDCl_3$)

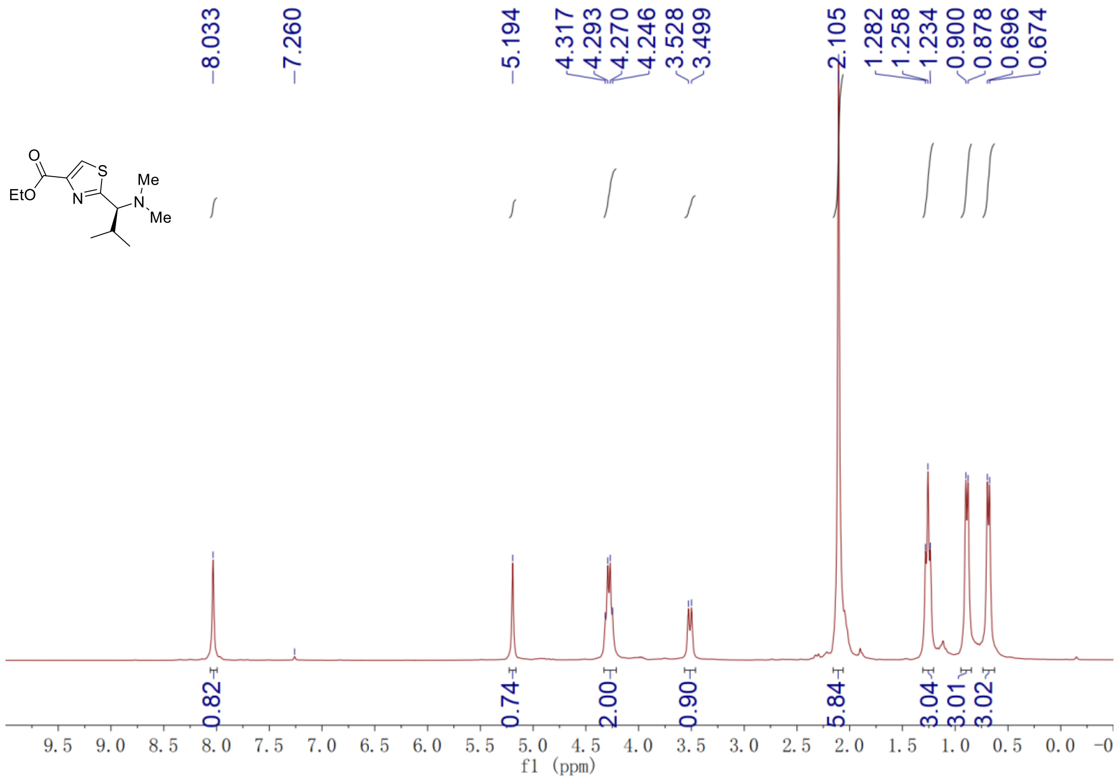

^{13}C NMR Spectrum of **S2** (75 MHz, $CDCl_3$)

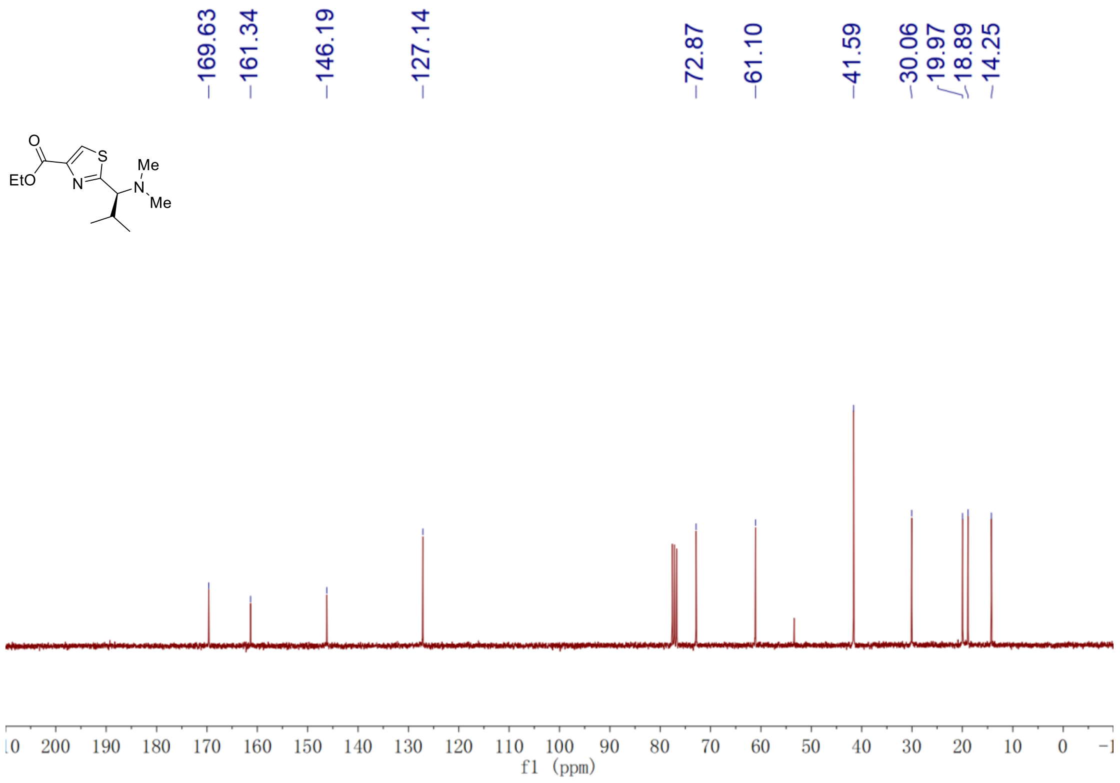

^{1}H NMR Spectrum of **3** (400 MHz, $CDCl_3$)

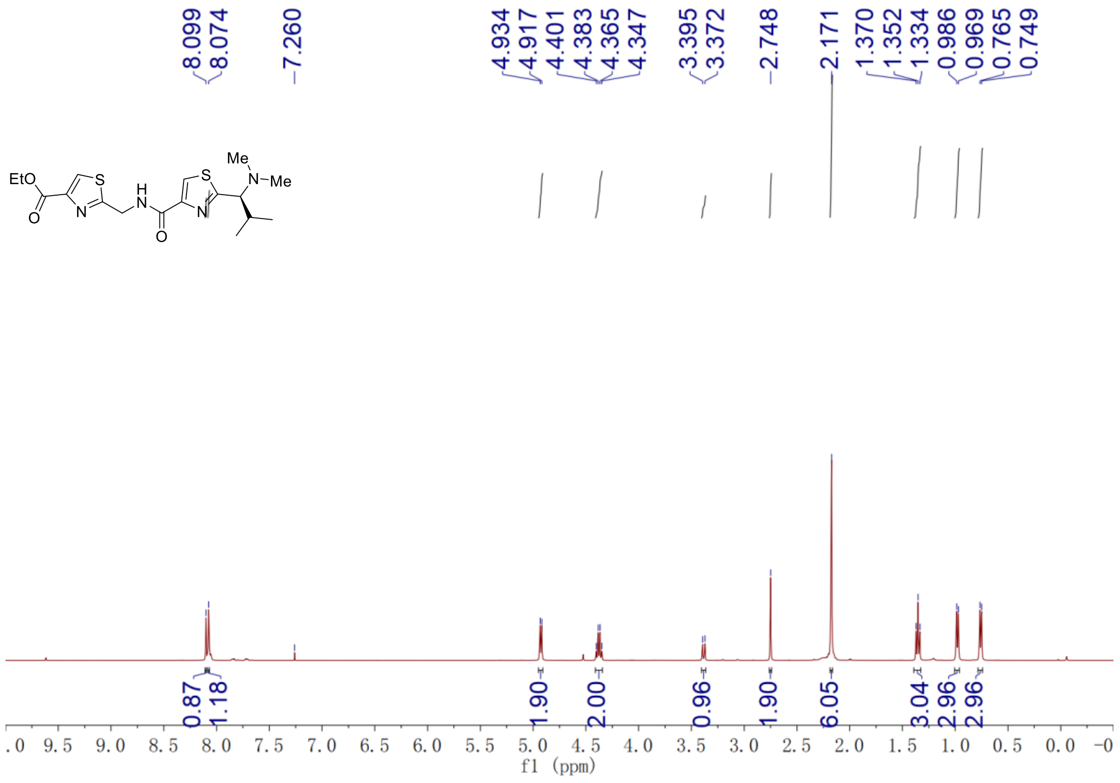

^{13}C NMR Spectrum of **3** (100 MHz, $CDCl_3$)

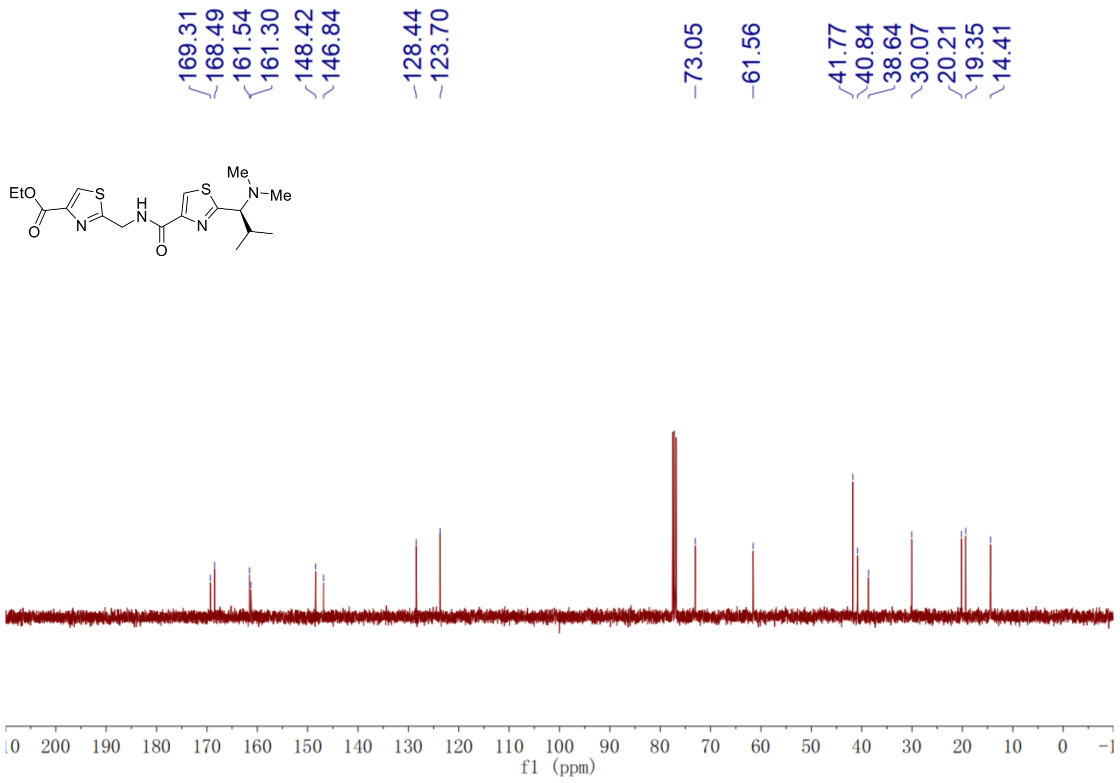

^{1}H NMR Spectrum of **2** (500 MHz, $CDCl_3$)

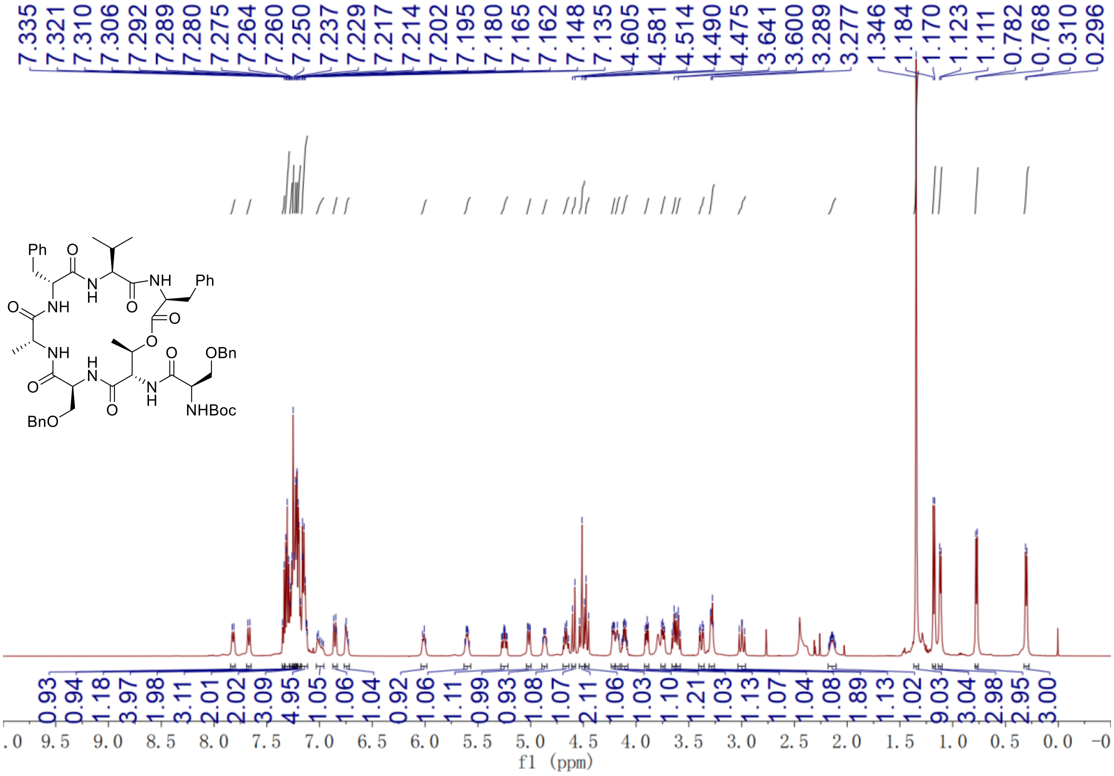

^{13}C NMR Spectrum of **2** (125 MHz, $CDCl_3$)

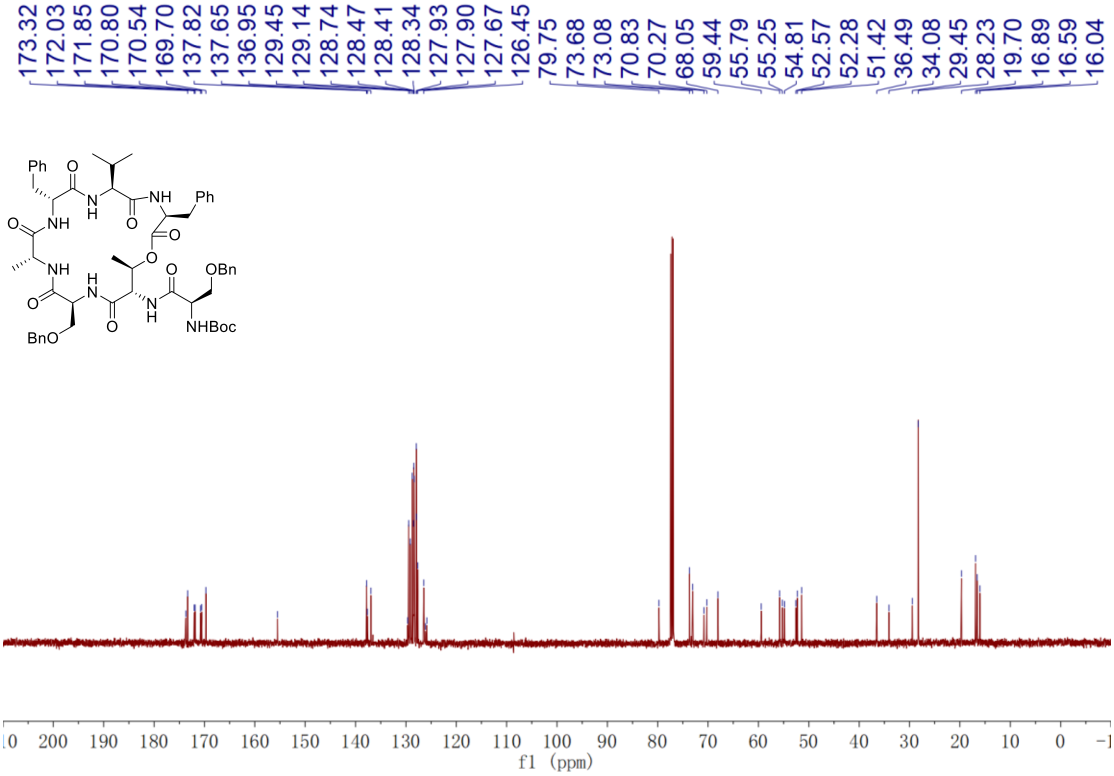

^{1}H NMR Spectrum of **S4** (500 MHz, $CDCl_3$)

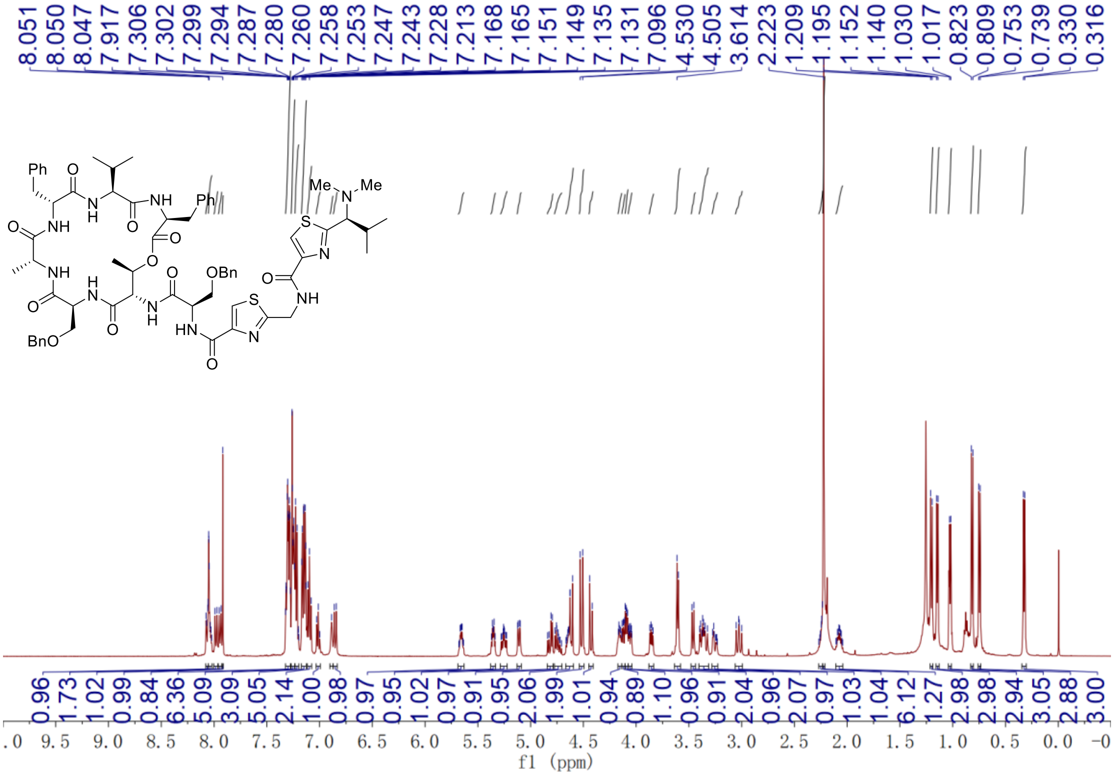

^{13}C NMR Spectrum of **S4** (125 MHz, $CDCl_3$)

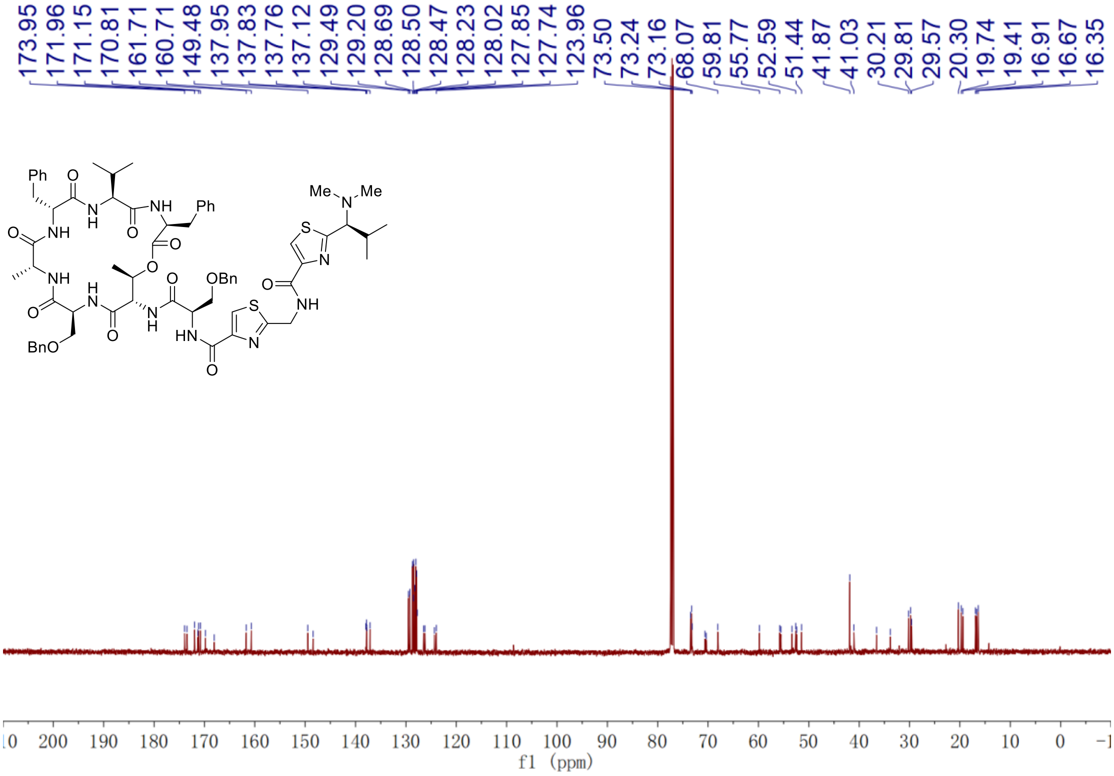

^{1}H NMR Spectrum of pagoamide A **(1)** (400 MHz, DMSO-d_6)

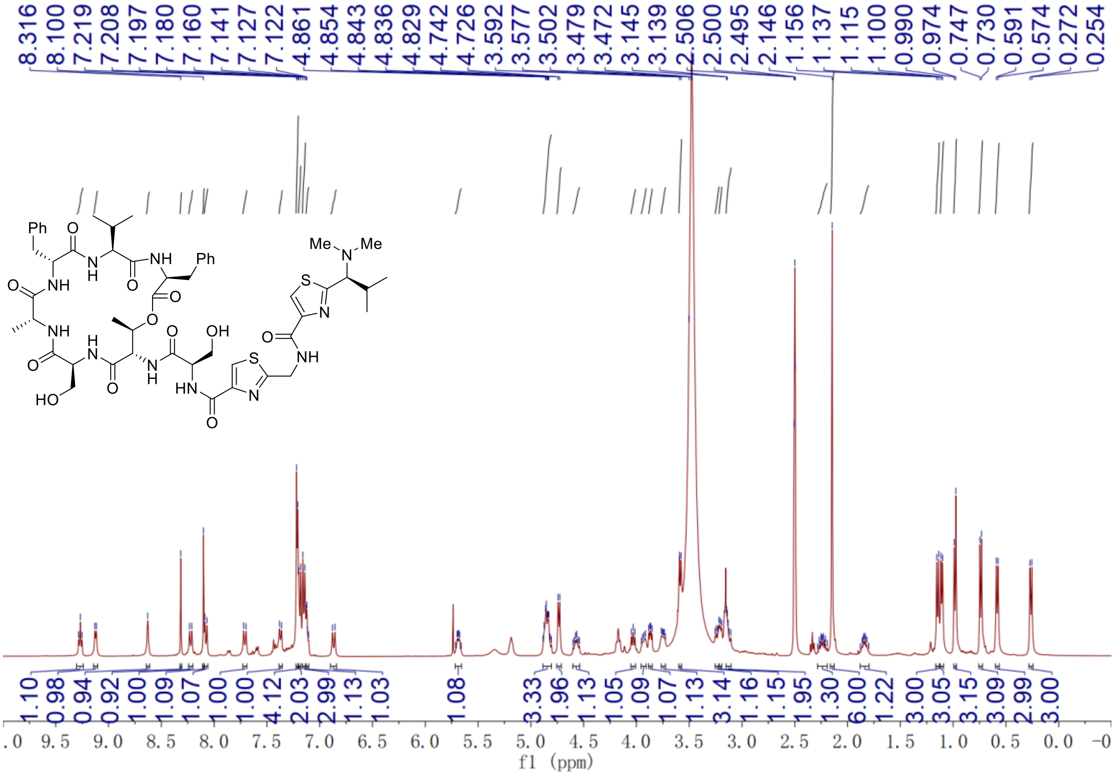

^{13}C NMR Spectrum of pagoamide A **(1)** (100 MHz, DMSO-d_6)

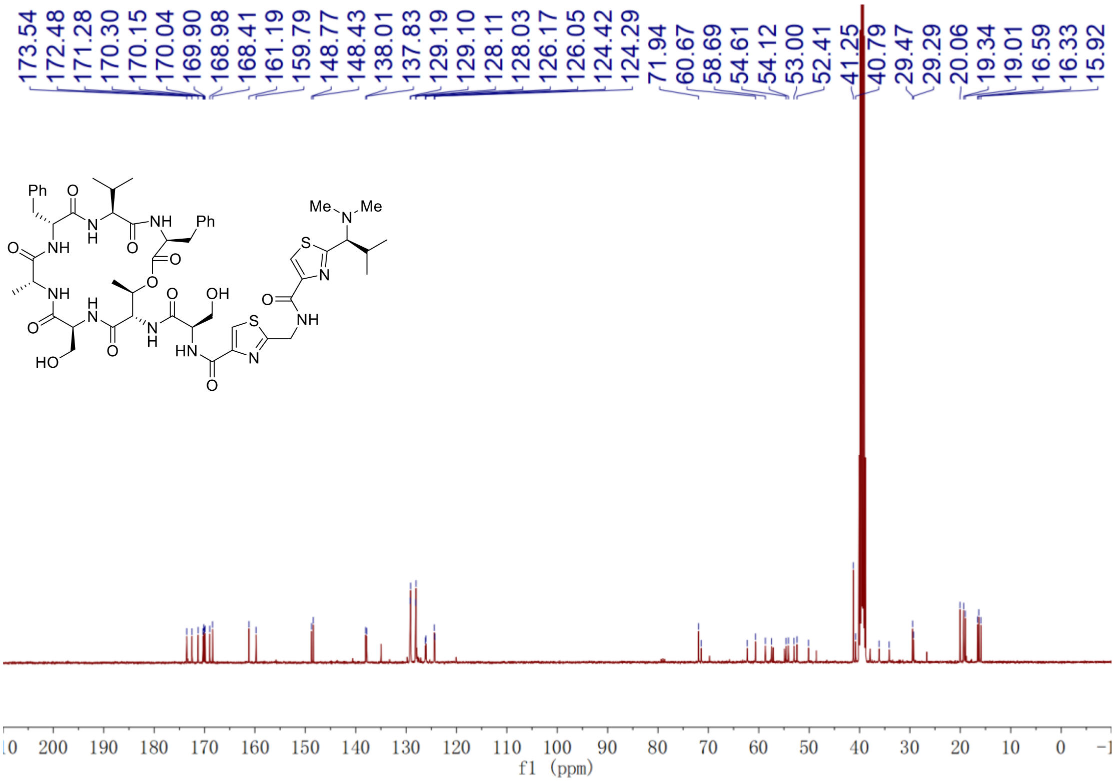

^{1}H NMR Spectrum of **S6** (400 MHz, $CDCl_3$)

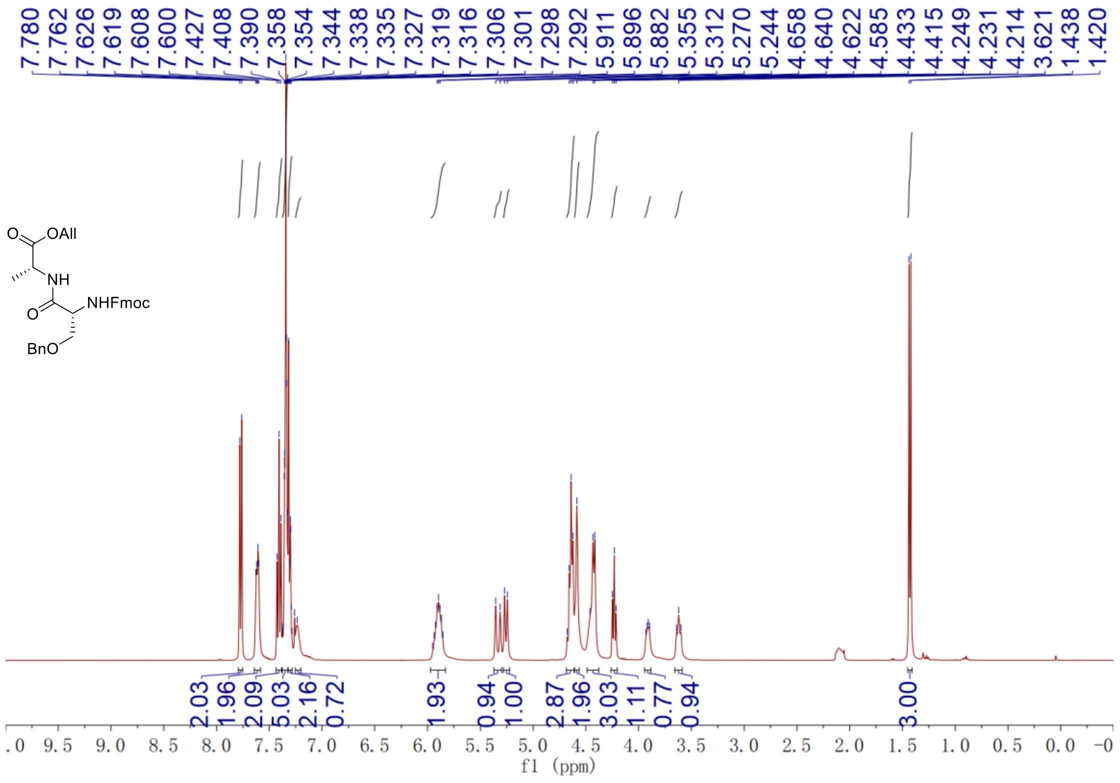

^{13}C NMR Spectrum of **S6** (100 MHz, $CDCl_3$)

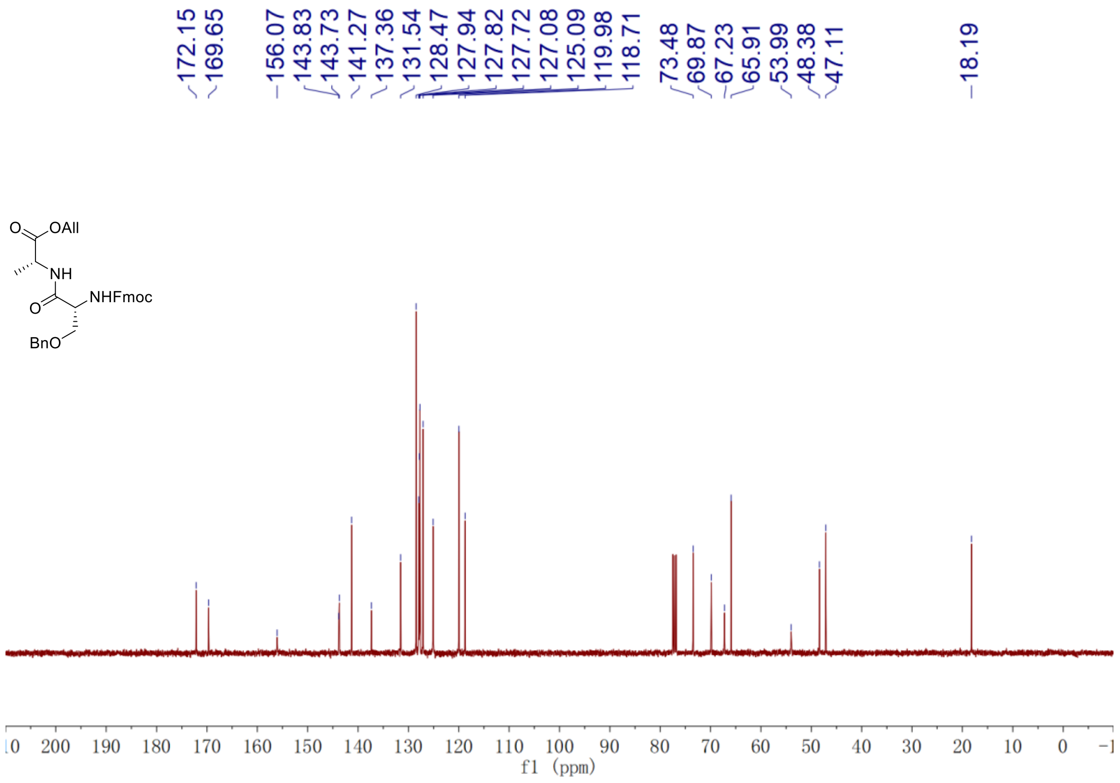

^{1}H NMR Spectrum of **S7** (500 MHz, $CDCl_3$)

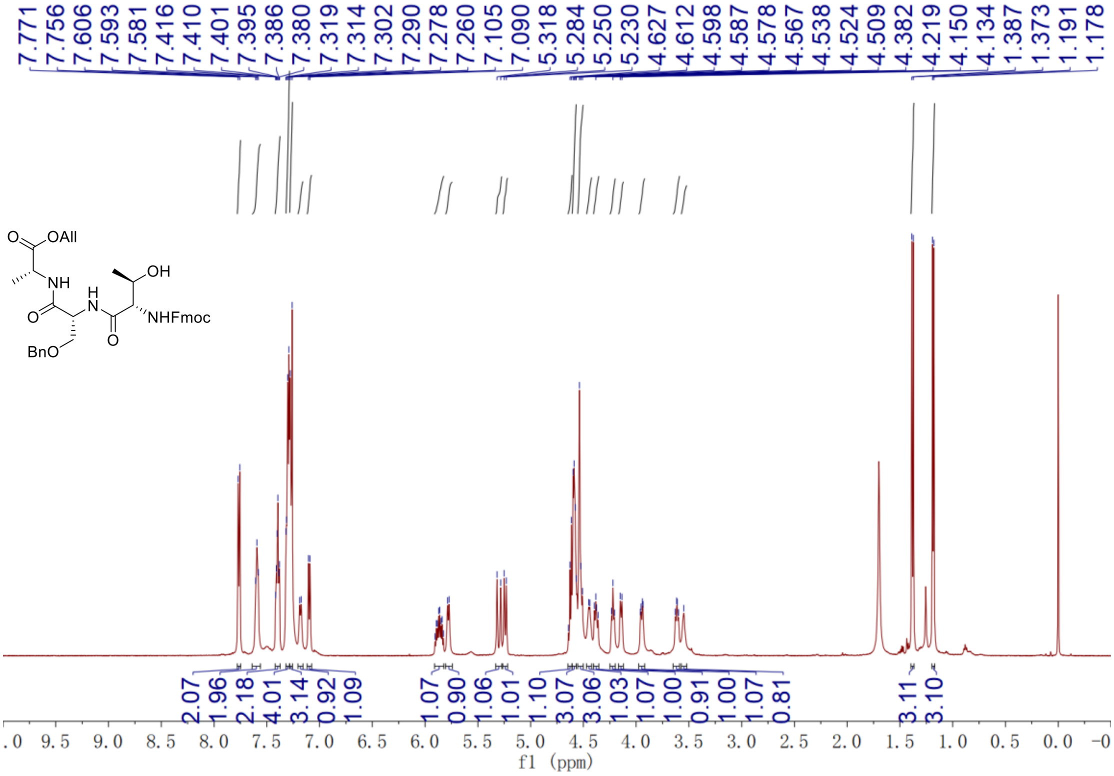

^{13}C NMR Spectrum of **S7** (125 MHz, $CDCl_3$)

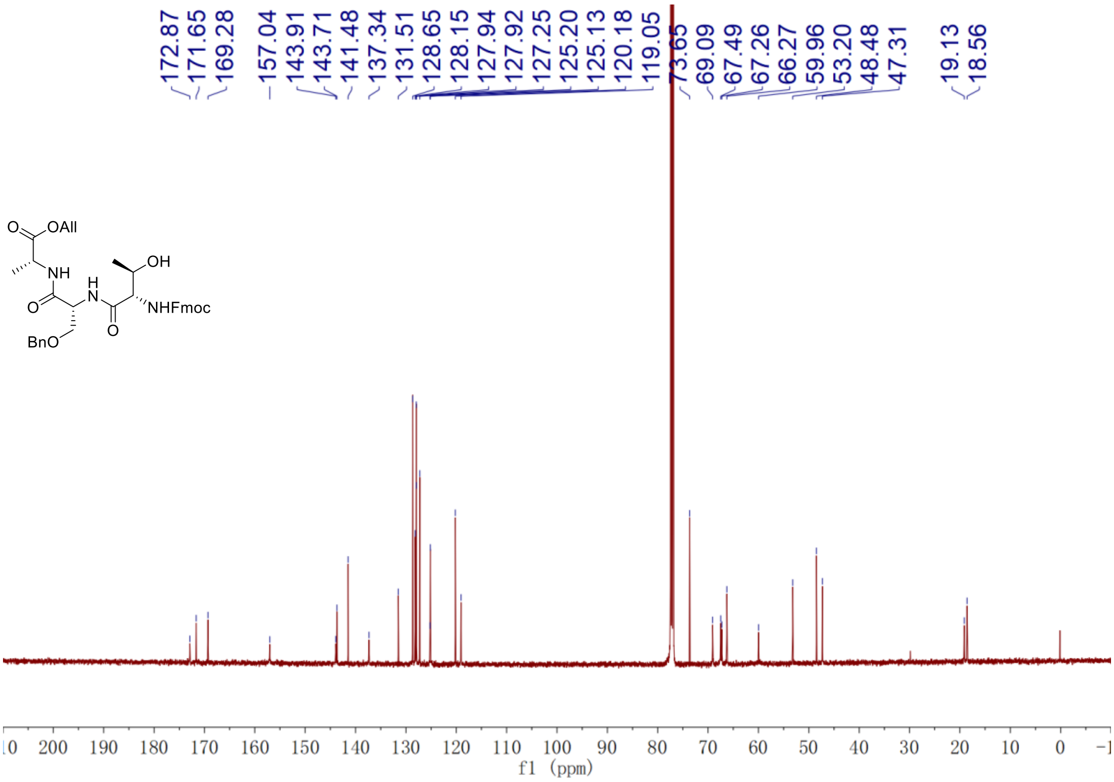

^{1}H NMR Spectrum of **S8** (500 MHz, $CDCl_3$)

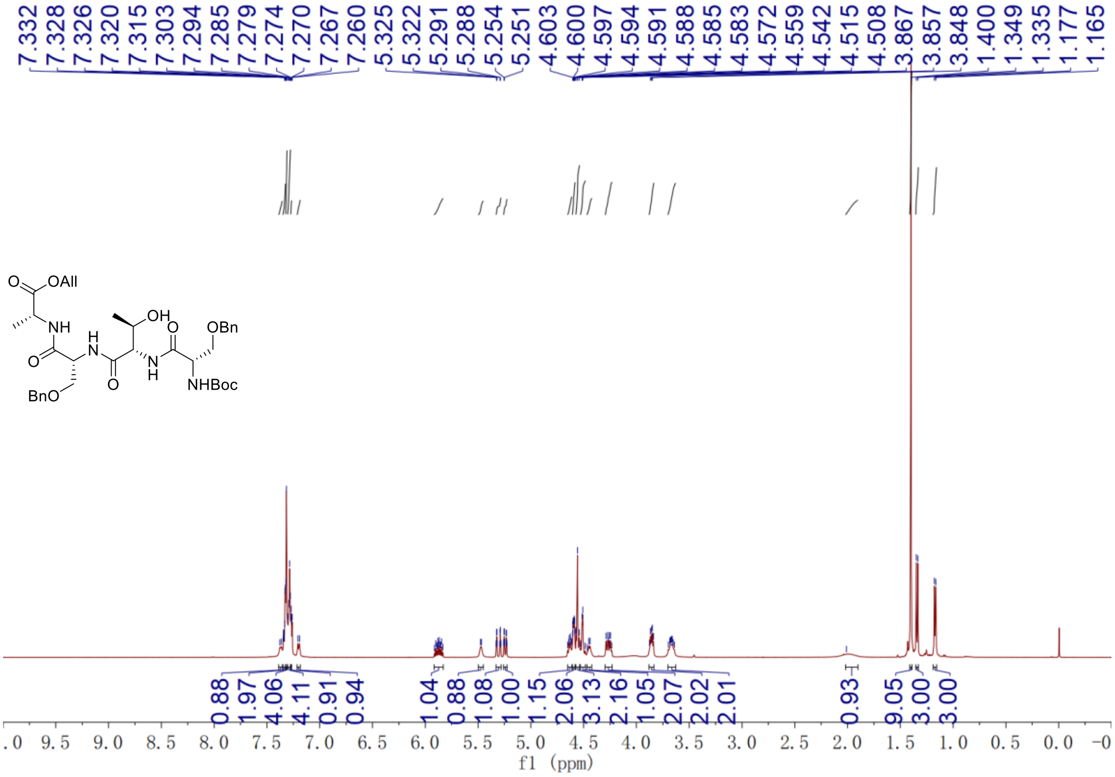

^{13}C NMR Spectrum of **S8** (125 MHz, $CDCl_3$)

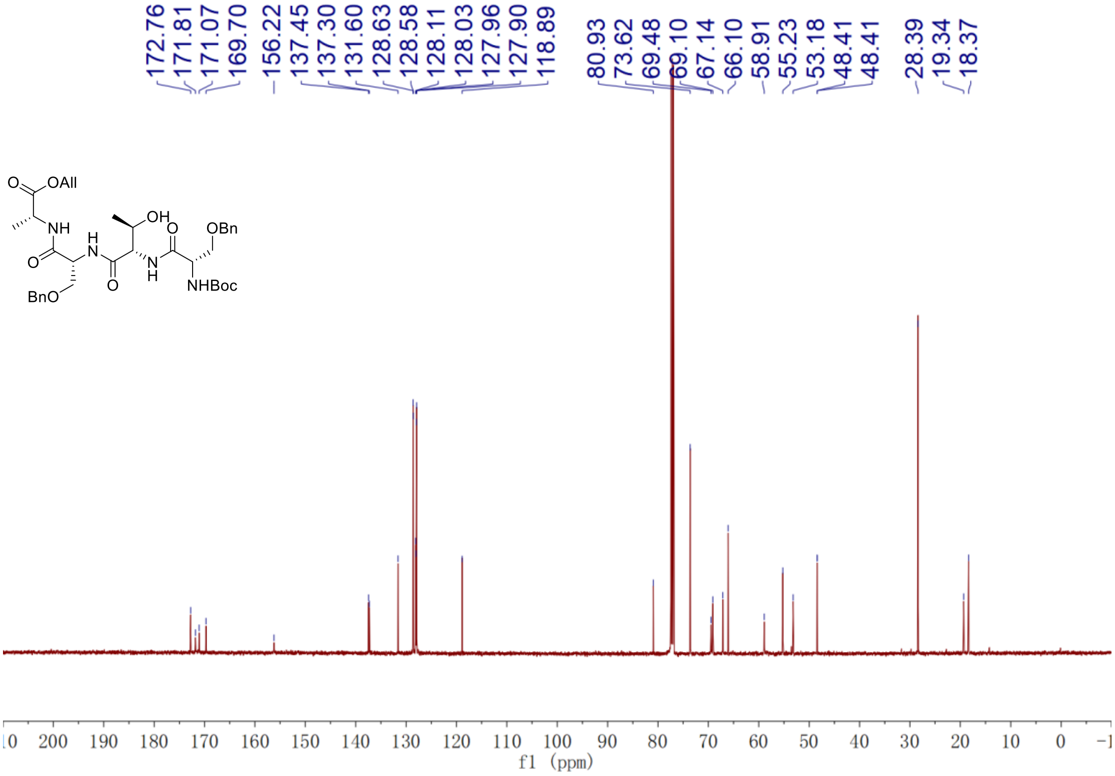

^{1}H NMR Spectrum of **S9** (300 MHz, $CDCl_3$)

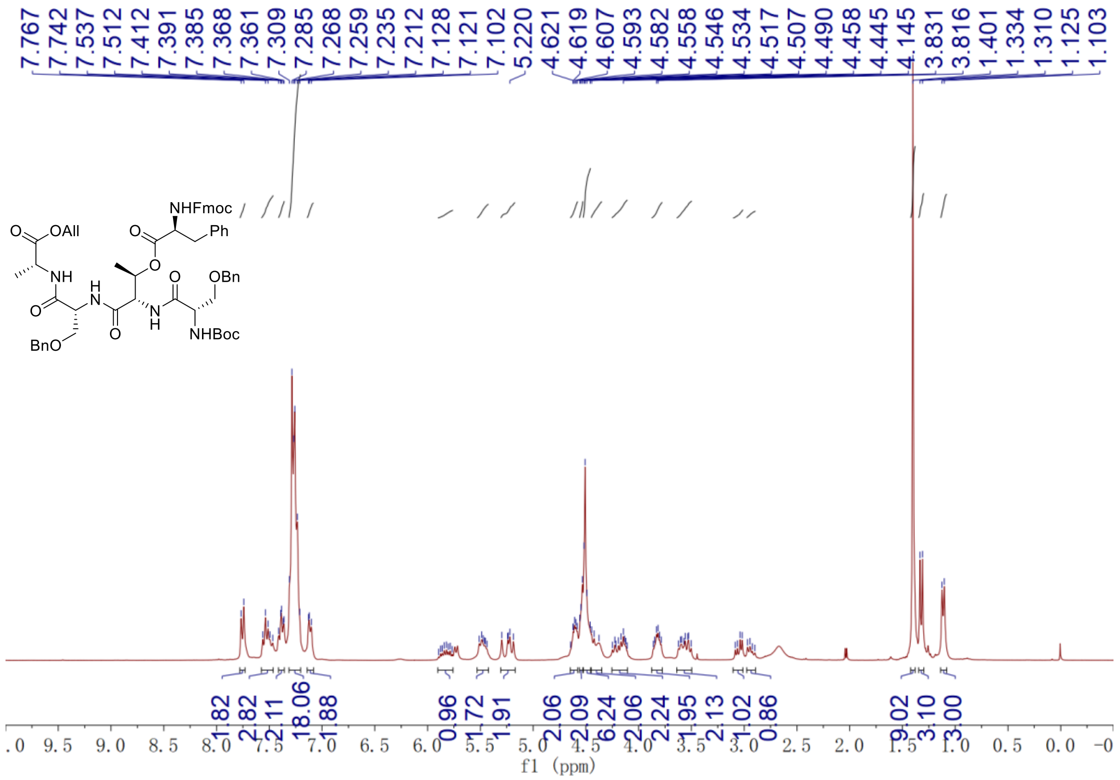

^{13}C NMR Spectrum of **S9** (75 MHz, $CDCl_3$)

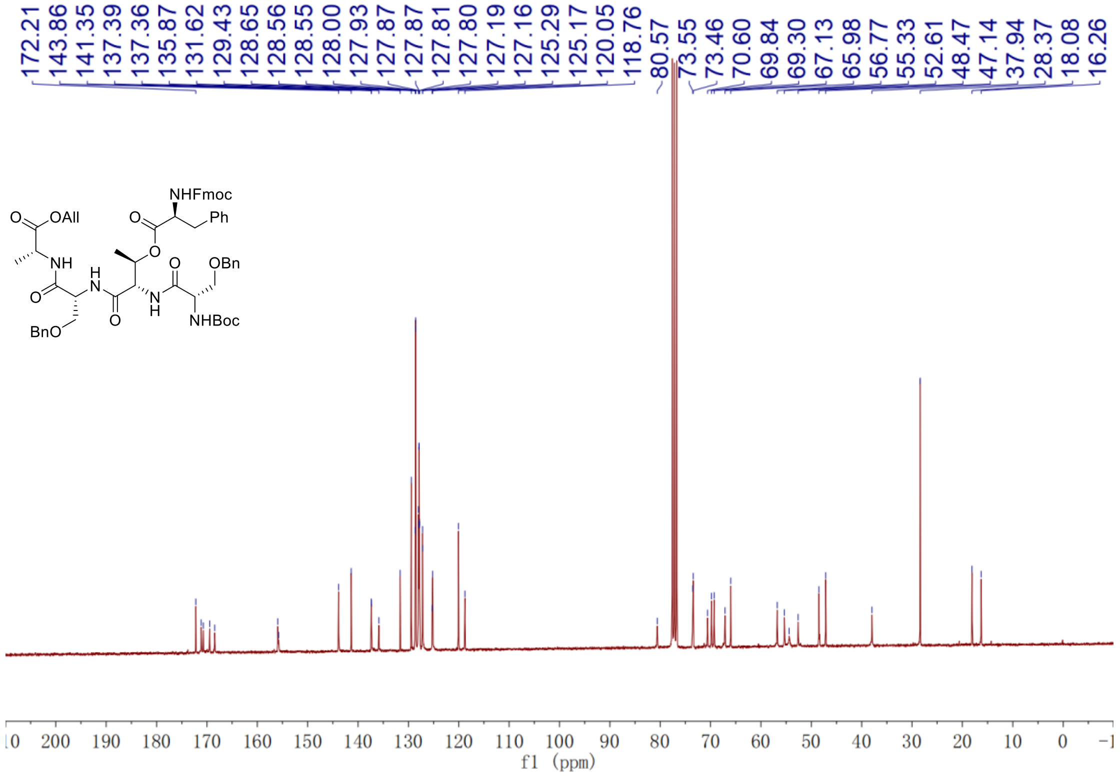

^{1}H NMR Spectrum of **S10** (500 MHz, $CDCl_3$)

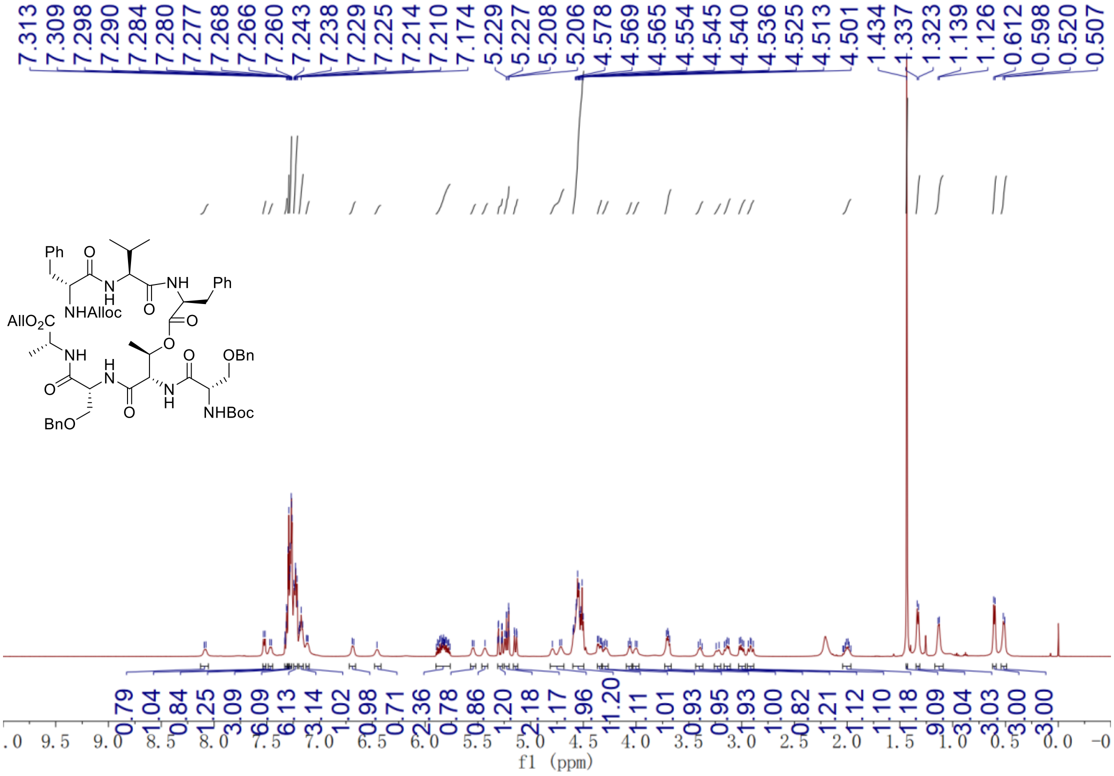

^{13}C NMR Spectrum of **S10** (125 MHz, $CDCl_3$)

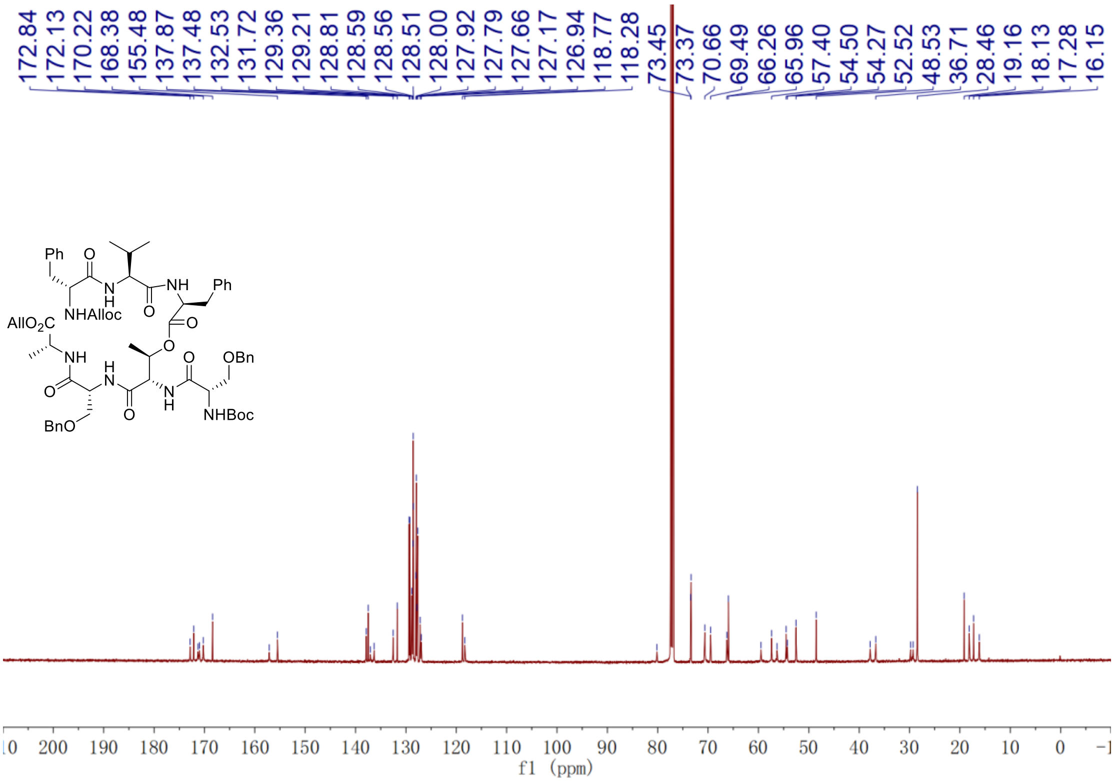

^{1}H NMR Spectrum of **S11** (400 MHz, $CDCl_3$)

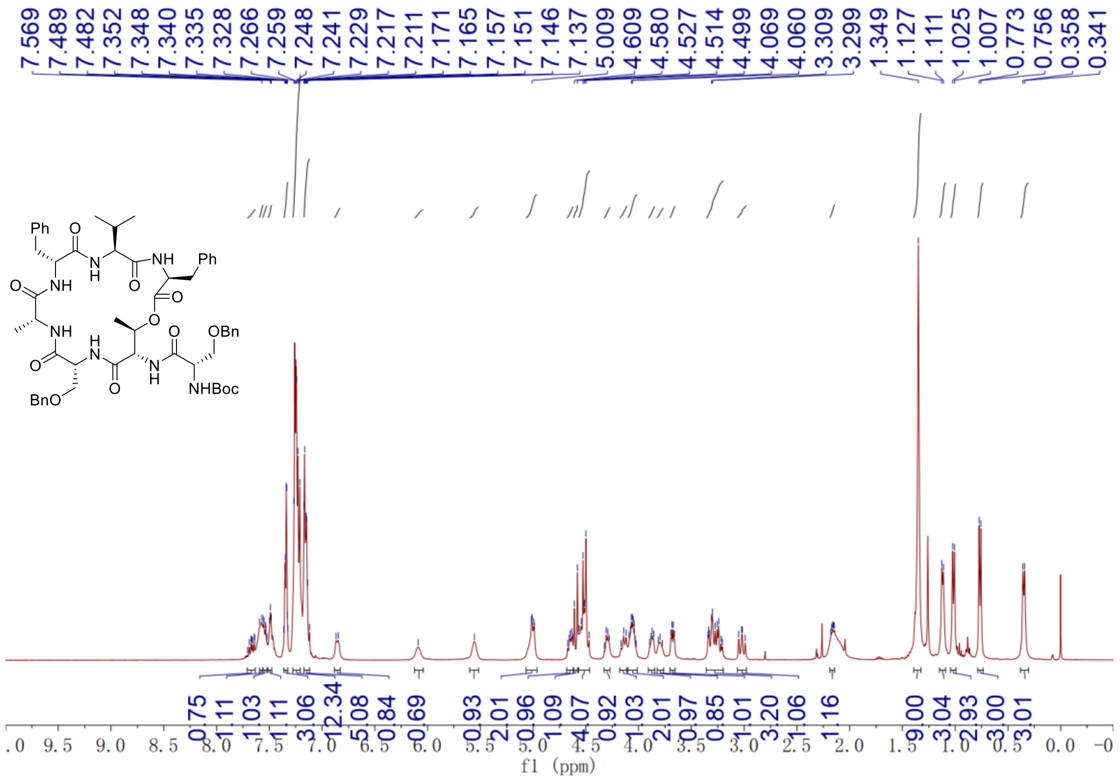

^{13}C NMR Spectrum of **S11** (100 MHz, $CDCl_3$)

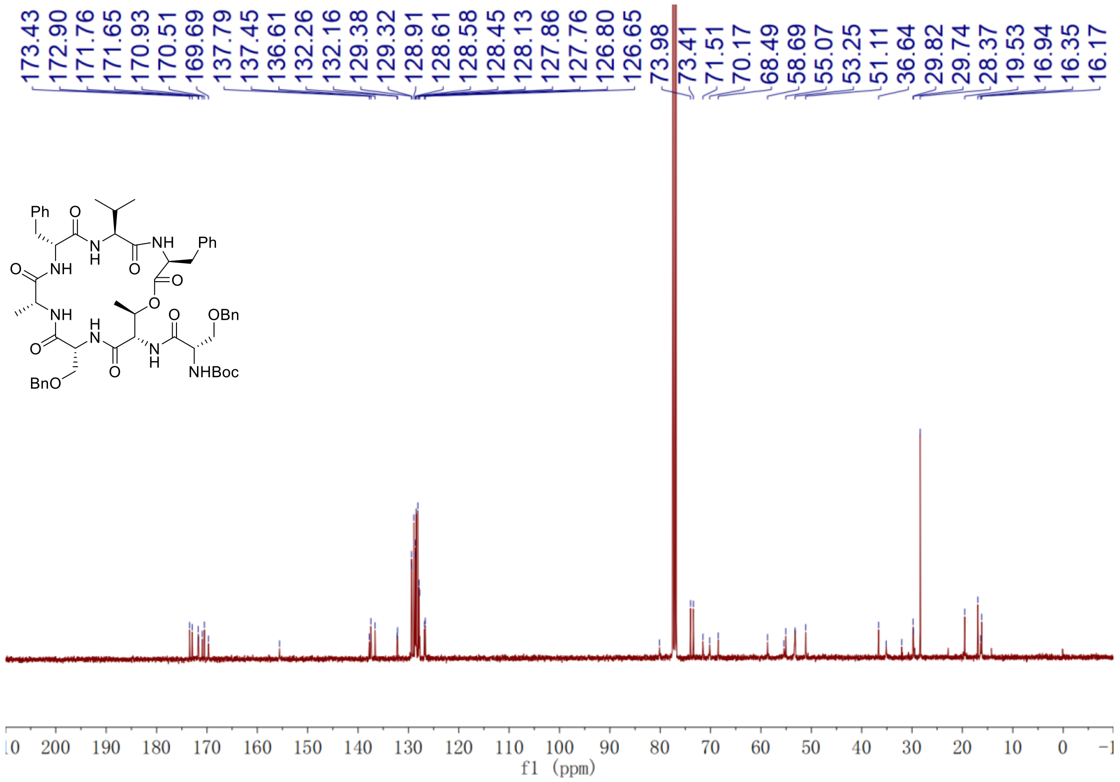

^{1}H NMR Spectrum of **S12** (300 MHz, $CDCl_3$)

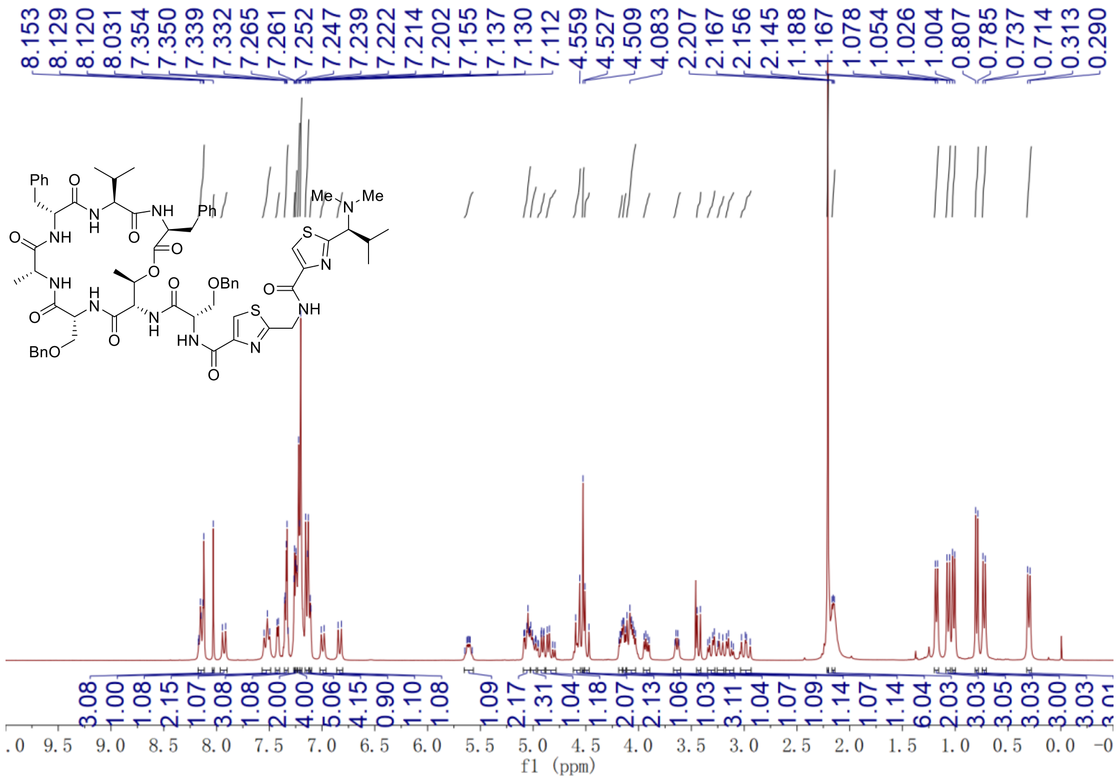

^{13}C NMR Spectrum of **S12** (75 MHz, $CDCl_3$)

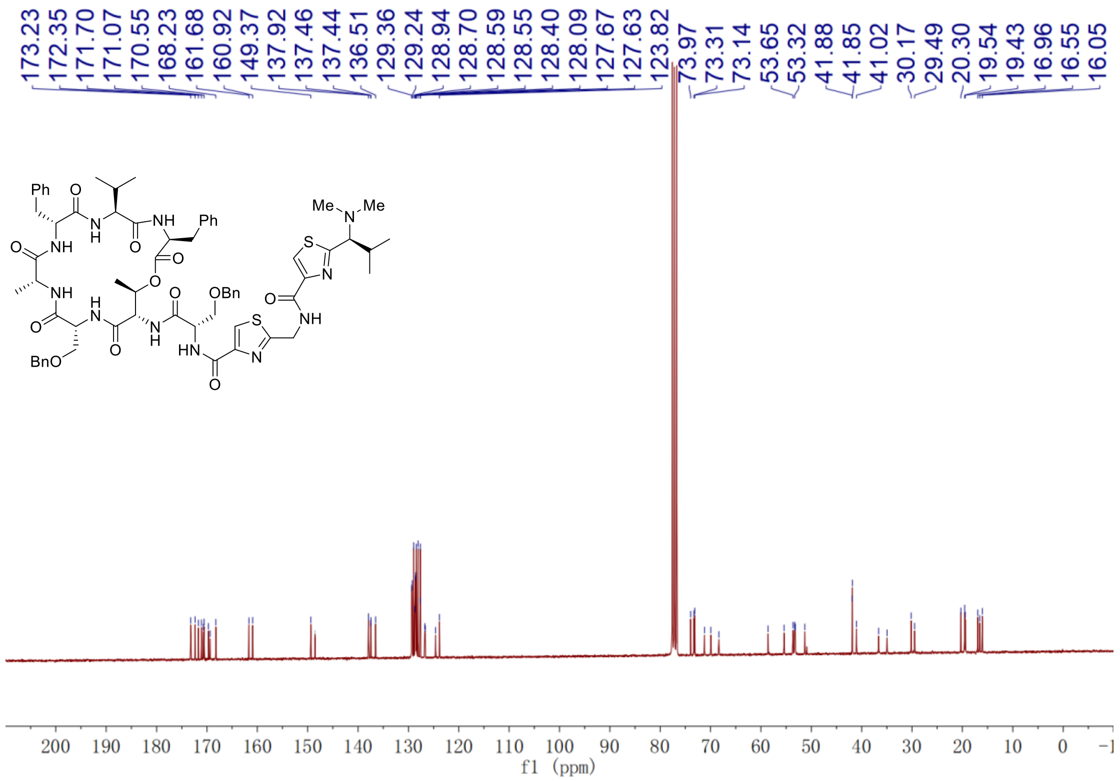

^{1}H NMR Spectrum of **1a** (500 MHz, DMSO-d_6)

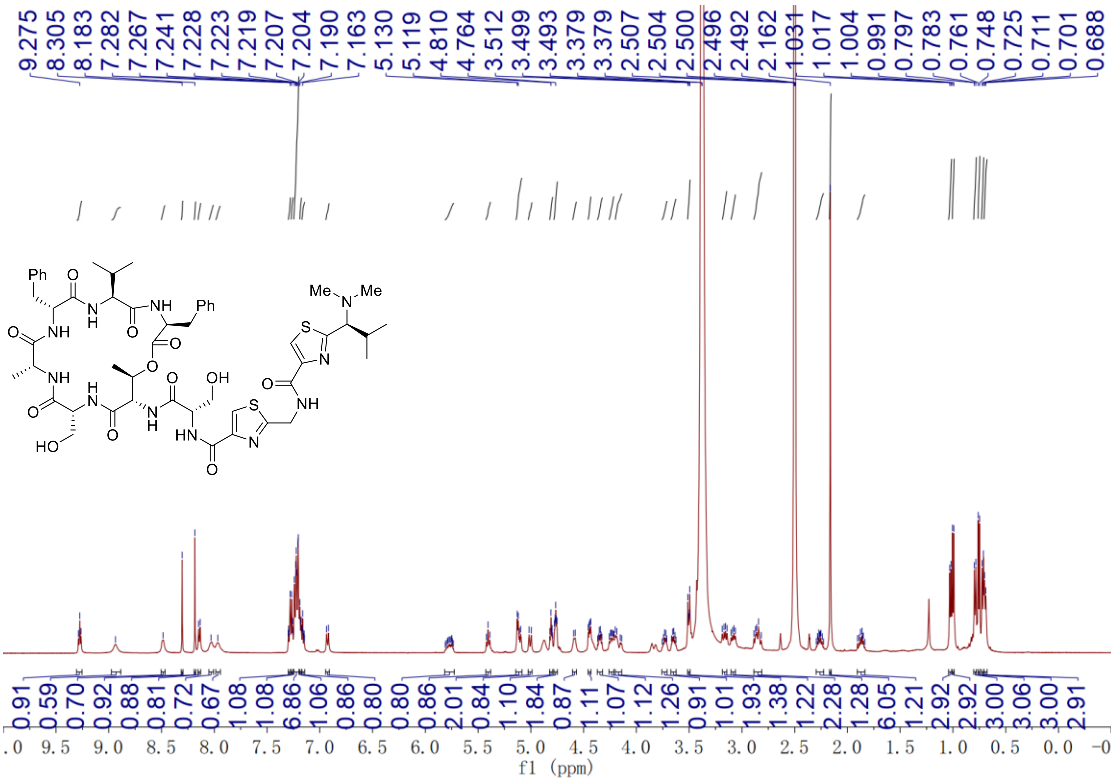

^{13}C NMR Spectrum of **1a** (125 MHz, DMSO-d_6)

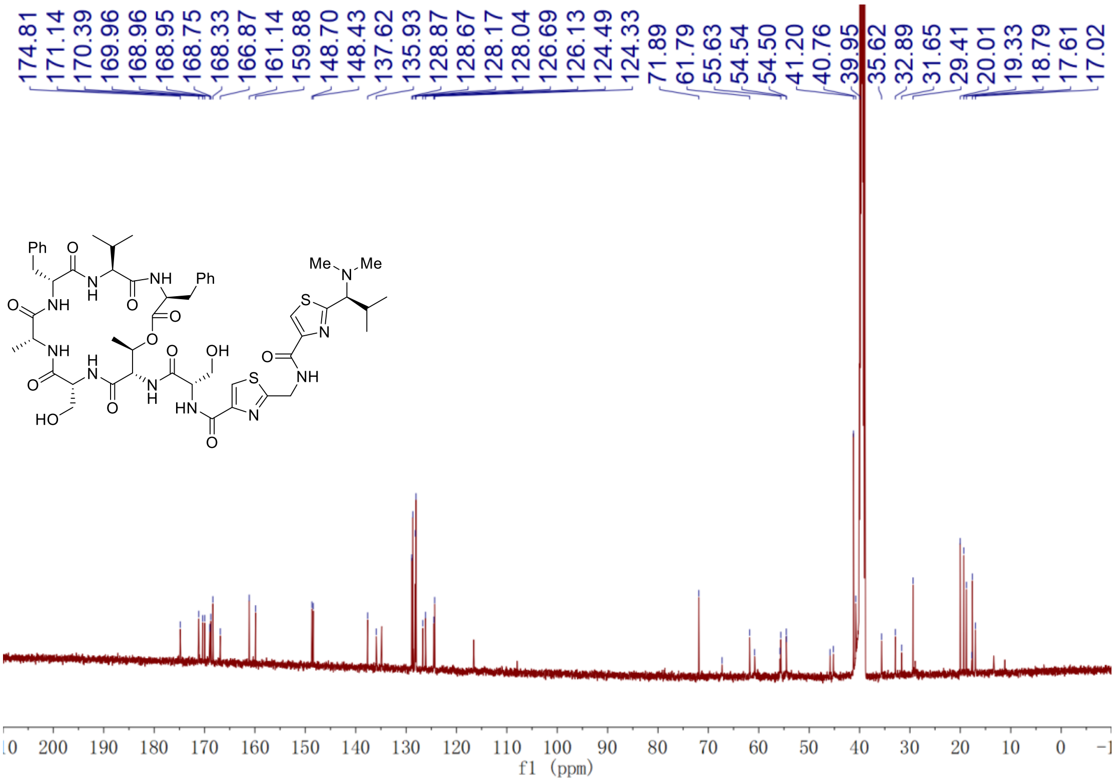

Supplement: Supplementary file 1 [file molecules-26-04224-s001.zip › molecules-1273165-supplementary.pdf]
